# Six-Membered Thiolate–Thione Chelate Complexes of Group 10 Elements—Syntheses, Crystal Structures, and NMR Studies

**DOI:** 10.1021/acsomega.5c06668

**Published:** 2025-10-15

**Authors:** Hennes Günther, Stephan Hohloch, Frank Tambornino

**Affiliations:** † Philipps University Marburg, Department of Chemistry, Hans-Meerwein-Str. 4, 35043 Marburg, Germany; ‡ University of Innsbruck, Department of General, Inorganic and Theoretical Chemistry, Innrain 80-82, A-6020 Innsbruck, Austria

## Abstract

Imido-*C*,*C’*-dithiodicarbonic-*O*,*O*’-dialkyl esters (alkyl: **1** = Me, **2** = Et, **3** = ^*i*^Pr) were synthesized by facile synthetic protocols. They form air-stable complexes with divalent group 10 ions (Ni­[**1**]_2_, Ni­[**2**]_2_, Ni­[**3**]_2_, Pd­[**1**]_2_, Pd­[**2**]_2_, Pd­[**3**]_2_, Pt­[**1**]_2_, Pt­[**2**]_2_, and Pt­[**3**]_2_). In their crystal structures, the metal center is coordinated by four sulfur atoms of two anionic ligands in a slightly distorted square planar fashion. The backbones of all complexes show distortion depending on the bulkiness of the ligand. A comparison of bond lengths in free ligands and their respective complexes reveals CS bond length extension and CN bond length shortening due to electron delocalization among the S–C–N–C–S moiety. The ^13^C NMR spectra show a dominating influence of the metal center, while the effect of the different alkyl chains is negligible.

## Graphical abstract

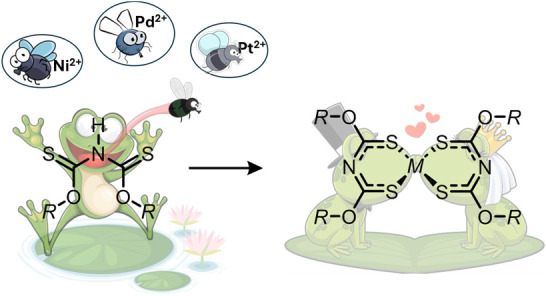

## Introduction

Sulfur ligands are classified as soft Lewis bases and are widely known for their role as ligands for soft metal centers. Prominent examples of sulfur-containing ligands are thioethers, especially thia-crown ethers, thiols, and thiocarbonyl compounds.^1–8^ The latter play a special role because of their comparatively unstable π bond.^9^ Therefore, compounds containing C=S moieties without any steric stabilization tend to oligo- or polymerize; a prominent example is the highly sensitive thioacetone.^10^ However, if a π-donating group is attached to the C=S moiety, stabilization is observed by donation of a lone pair into the π* of the C=S bond. This is, for example, observed in thiourea. In the solid state, this stabilization manifests by lengthening of the C=S and shortening of the C–N bond.^11–13^

Previously, we reported the synthesis and reactivity of the chelating sulfur ligand imido-*C*,*C’*-dithiodicarbonic-*O*,*O*’-diethyl ester and its nickel complex.^14^ The methyl, ethyl, and isopropyl analogues of this ligand were first described in 1980 and synthesized by reacting thiocarbonyl dithiocyanate with the respective alcohol.^15^ Complexation of Ni^2+^ utilizing imido-*C*,*C’*-dithiodicarbonic-*O*,*O*’-diethyl ester in ethanol suspension in the presence of pyridine yields Ni­[C_2_H_5_OC­(S)­NC­(S)­OC_2_H_5_]_2_ (Ni[2]_2_).^14^ In its single crystal structure, the nickel center is coordinated in a square planar fashion by two [2]^−^ units forming two hexacycles with the Ni^2+^ cation. A tilted backbone of the ligand in the complex is observed, and similar tilting is present in dithiobiuret complexes of group 10 elements.^12,13^

Related transition metal complexes with –NH_2_, –NR_2_ (R = Me, Et), and one-sided substituted PR_2_ (R = Ph) instead of alkoxy moieties are reported in the literature.^16–20^ Interestingly, nickel complexes bearing the NR_2_ (R = Me, Et) moiety can be utilized for aerosol-assisted chemical vapor deposition of nickel sulfides.^18^

In this contribution, we revisit the synthesis of ligands **1** and **3** and, building upon our previous contribution, present the synthetic protocol of new group 10 complexes alongside two alkali metal salts of imido-*C*,*C’*-dithiodicarbonic-*O*,*O*’-dialkyl ester (R = Me, Et, ^*i*^Pr, Figure 1). We discuss their syntheses, spectroscopic signatures, and structures based on single crystal X-ray diffraction.

**Figure 1:**
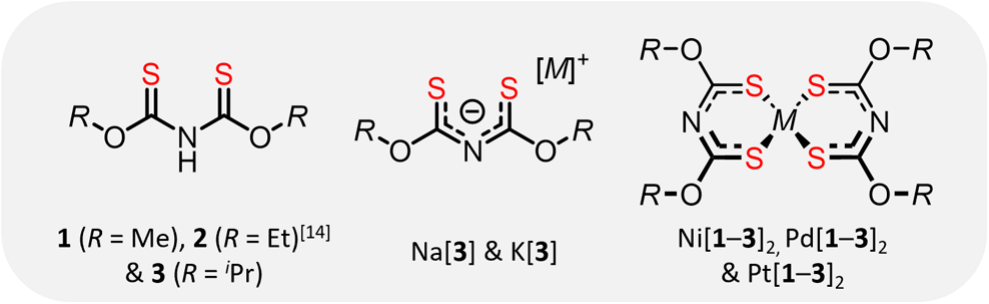
Lewis structures of synthesized ligands and their alkali and group 10 transition metal complexes.

## Results and Discussion

### Syntheses and Crystal Structures of **1** and **3**

Thiocarbonyl dithiocyanate (TCDT) is a highly air-sensitive orange solid that decomposes at temperatures above −20 °C. The thiocarbonyl and thiocyanate carbon atoms are electrophilic centers and react rapidly with nucleophiles like alcohols. In our previous study, we presented the reaction of TCDT with ethanol and examined the obtained ligand **2** with respect to its NMR signatures, single crystal X-ray structure, and ability to act as a ligand toward Ni^2+^.^14^ Generalizing the reaction of TCDT with other nucleophiles, we propose a three-stage mechanism (Scheme 1). First, the reaction of TCDT with one equivalent of alcohol leads to the formation of an alkoxythiocarbonyl thiocyanate. This intermediate isomerizes likely by an intermolecular process to alkoxythiocarbonyl isothiocyanate, which ultimately reacts with a second equivalent of alcohol to afford the ligand.

**Scheme 1:**
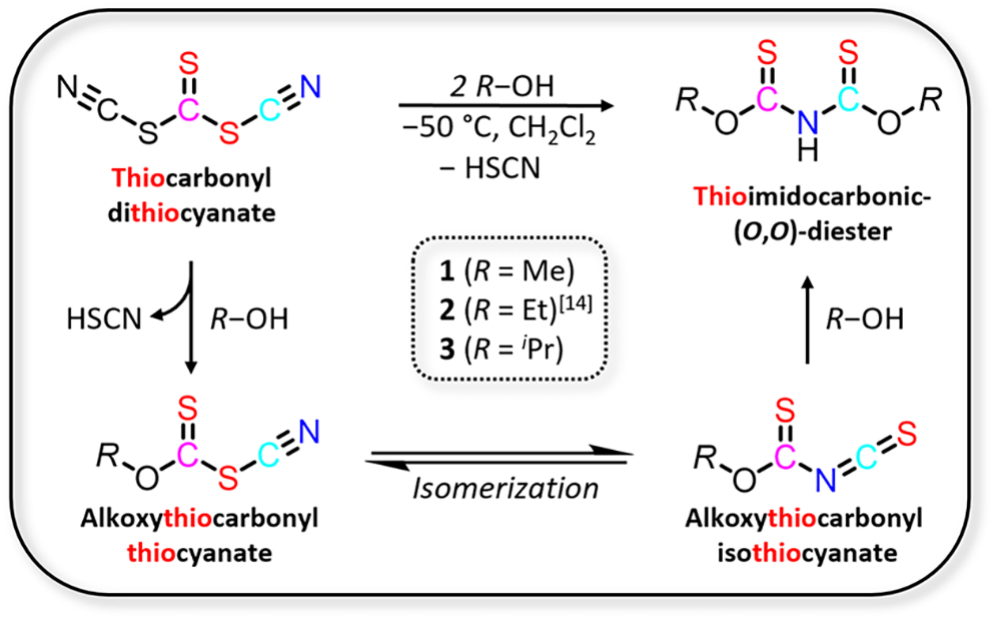
Proposed Mechanism for the Synthesis of 1–3 from Thiocarbonyl Dithiocyanate

We have tested the reactivity of TCDT toward a series of alcohols (*R*–OH, R = Me, ^*i*^Pr, ^*t*^Bu, Ph, and Bn). Interestingly, only the reaction of methanol and *iso*-propanol leads to the formation of the respective imido-*C*,*C’*-dithiodicarbonic-*O*,*O*’-dialkyl esters (**1** and **3**, in −50 °C dichloromethane suspension). In the case of benzyl alcohol, phenol, and *tert*-butanol, the reaction mixture turned brown or/and red (see section 2.13 in the SI). Because of the insoluble nature of the obtained residue, we assume that one of the decomposition products is pararhodane, which indicates decomposition of sulfur-containing species. Apparently, aromatic alcohols and alcohols with acidic β-H atom are not suitable starting materials for imido-*C*,*C’*-dithiodicarbonic-*O*,*O*’-di ester. For the reaction of TCDT with simple aliphatic alcohols (*R*–OH with R = Me, Et, and ^*i*^Pr), two observations can be deduced. (a) With increasing length of the aliphatic chain of the alcohol, the yields increase (**1**, 21%; **2**, 55%; **3** 66%). (b) With increasing size of the aliphatic chain, the color of the bulk material intensifies (**1**, faint yellow; **2**, yellow-orange; **3**, orange-red). Single crystals of **1**–**3** suitable for single crystal X-ray diffraction were obtained by slowly evaporating the solvent of a saturated solution in *n*-hexane. The single crystal X-ray structures of **1** and **3** are shown in Figure 2.

**Figure 2:**
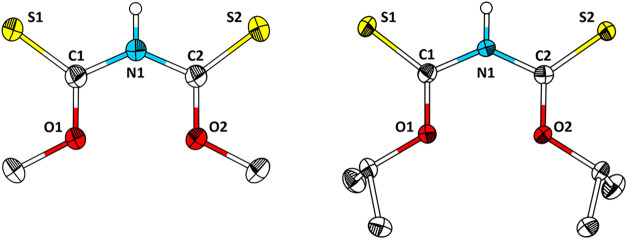
Structure of **1** (left) and **3** (right) in the single crystal. Ellipsoids are drawn with a 40% probability level. Aliphatic hydrogen atoms are omitted for clarity. Selected bond lengths (Å) and angles (°) of 1 and 3: C1–O1, 1.303(3), 1.314(1); C1–S1, 1.657(2), 1.651(1); C1–N1, 1.377(3), 1.390(2); N1–C2, 1.377(3), 1.375(2); C2–O2, 1.311(3), 1.313(1), C2–S2, 1.648(2), 1.663(1).

**1** crystallizes in the triclinic crystal system with space group *P*1̅ (*a* = 5.8447(5) Å, *b* = 7.8279(7) Å, *c* = 8.3451(6) Å, α = 84.228(7)°, β = 78.480(6)°, γ = 76.575(7)°) with one independent molecule in the asymmetric unit and two molecules in the unit cell (*Z* = 2). The bond lengths of C1 = S1 and C2 = S2 are in between a CS single (1.78 Å)^21^ and double bond (1.61 Å).^21^ Compared to the corresponding bond lengths of dithiobiuret (1.673(3)/1.702(3) Å), they are slightly shorter.^11^ The C–N bond lengths (1.311(3) Å and 1.390(2) Å) are in between a CN single (1.46 Å) and double bond (1.27 Å) and are in accordance with the corresponding bond lengths in dithiobiuret (1.367(4) Å).^11^

**3** crystallizes in the monoclinic system with space group *P*2_1_/*n* (*a* = 9.9684(3) Å, *b* = 8.0354(2) Å, *c* = 14.9997(5) Å, β = 106.461(2)°) with one independent molecule in the asymmetric unit and four molecules in the unit cell (*Z* = 4). The molecular structure closely resembles that of **1** and the previously published **2**.^14^

### Attempts to Synthesize Li­[**2**]

The N–H proton was shown to be acidic, since reaction with NiCl_2_ in ethanol in the presence of a base (e.g., pyridine) yields the complex Ni­[**2**]_2_ and pyridinium chloride as a side product. Reaction of **2** with pyridine in diethyl ether solution without the presence of NiCl_2_ did not yield the pyridinium salt. For the synthesis of Li­[**2**], the neutral ligand was deprotonated with *n*-butyllithium at −50 °C in *n*-hexane. Bulk analysis by NMR and IR spectroscopy and elemental analyses discloses a complex reaction mixture. Attempts to crystallize the obtained beige solid were unsuccessful and led to a gel-like precipitate. However, sequestering the lithium ion with 12-crown-4 leads to the formation of a small quantity of colorless crystals, which turn out to be Li­[**2**]. The single crystal X-ray structure of [Li@12-crown-4]­[**2**] is shown in Figure 3.

**Figure 3:**
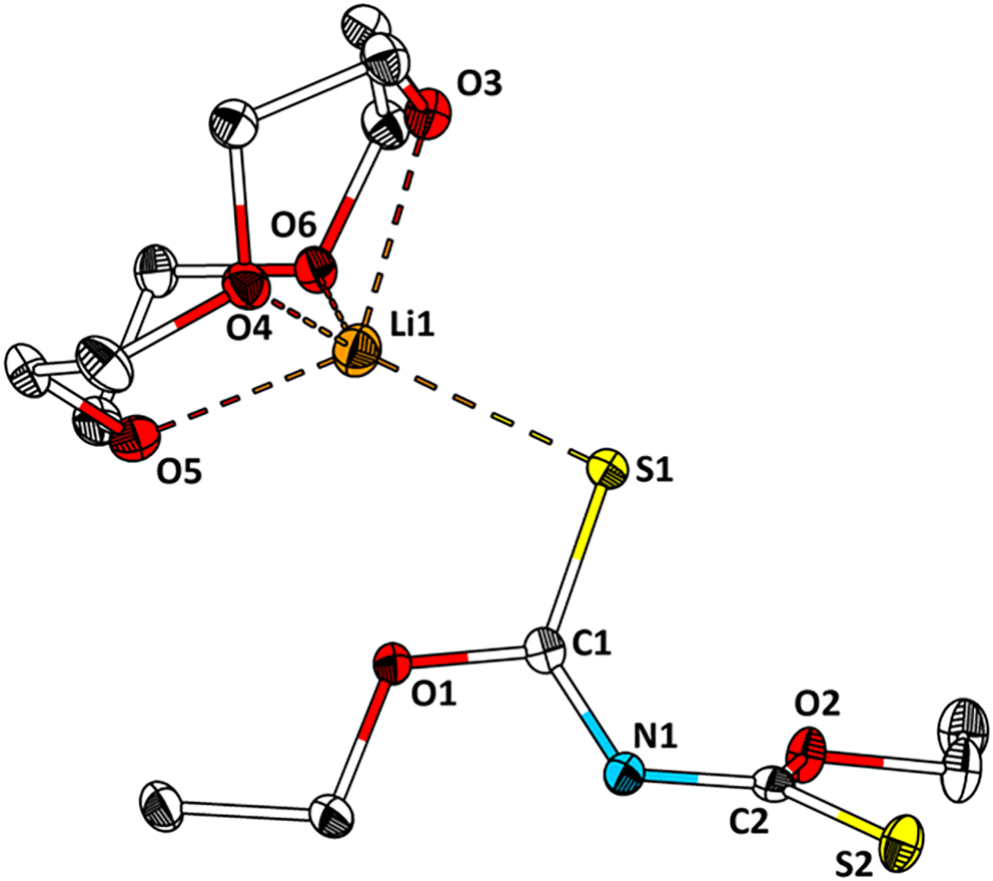
Structure of [Li@12-crown-4]­[**2**] in the single crystal. Ellipsoids are drawn with a 50% probability level. Hydrogen atoms have been omitted for the sake of clarity. Selected bond lengths (Å) and angles (°): Li1–O3, 2.179(3); Li1–O4, 2.087(3); Li1–O5, 2.141(3); Li1–O6, 2.138(2); Li1–S1, 2.456(3); S1–C1, 1.709(1); C1–O1, 1.351(2); C1–N1, 1.302(2); N1–C2, 1.343(2); C2–S2, 1.667(1); C2–O2, 1.342(1).

[Li@12-crown-4]­[**2**] crystallizes in the monoclinic crystal system with space group *P*2_1_/*n* (*a* = 12.3241(2) Å, *b* = 8.2730(1) Å, *c* = 18.5714(4) Å, β = 98.027(2)°) with one independent molecule in the asymmetric unit and two molecules in the unit cell (*Z* = 2). Li1 is coordinated by four oxygen atoms of 12-crown-4 and the S1 atom of anionic [**2**]^−^. The C1–S1 (1.709(1) Å) bond length is significantly longer than that of C2–S2 (1.667(1) Å) and the equivalent bond in **2** (1.661(3) Å, 1.65(3) Å). The C1–O1 (1.351(2) Å) and C2–O2 (1.342(1) Å) bond lengths are approximately similar and slightly longer compared to **2** (1.315(3) Å, 1.311(3) Å). The C1–N1 (1.302(2) Å) and N1–C2 (1.343(2) Å) bond lengths differ as significantly as the C1(2)–S1(2) bond lengths. Furthermore, the twisting angle about C1–N1–C2–S2 is 125.7°. The bond lengths and angles suggest a thioenolate structure of the [**2**]^−^ in the solid state (Scheme 2).

**Scheme 2:**
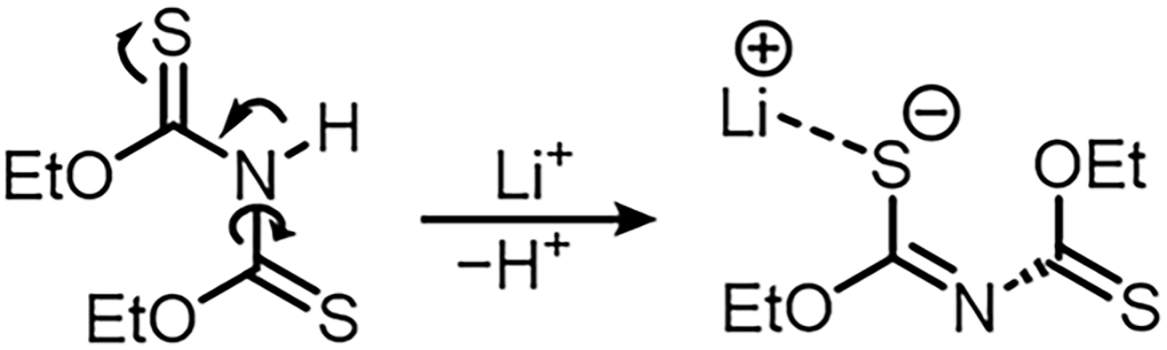
Formation of a Thioenolate Structure by Deprotonation

### Syntheses and Crystal Structures of Na[3] and K­[**3**]

Since the synthesis of Li­[**2**] did not yield a pure and isolable compound, we studied whether weaker bases and other counterions could be used for the synthesis of the alkali metal salts of **1**–**3**. We reacted **3** with potassium *tert*-butoxide (KO^*t*^Bu) in −50 °C diethyl ether and sodium hexamethyldisilazide in 0 °C diethyl ether and observed Na­[**3**] and K­[**3**] as slightly air-sensitive beige solids in 60 and 71% yield, respectively. Various crystallization attempts to crystallize Na­[**3**] were unsuccessful. However, single crystals of K­[**3**] were obtained by layering a THF solution of K­[**3**] with *n*-hexane. The single crystal X-ray structure is shown in Figure 4. An analogous attempt with crown ether leads to single crystals of K@18-crown-6­[SCN], which are most likely attributed to a decomposition of K­[**3**].

**Figure 4:**
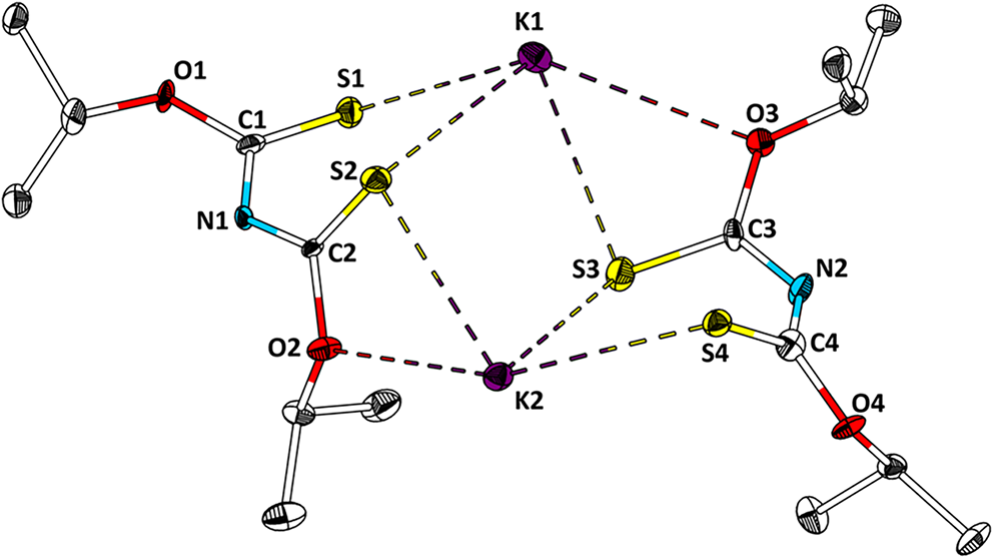
Structure of K­[**3**] in the single crystal. Ellipsoids are drawn with a 50% probability level. Hydrogen atoms are omitted for clarity. Selected bond lengths (Å) and angles (°): K1–S1, 3.212(3); K1–S2, 3.276(3); K1–S3, 3.133(3); K1–O3, 3.257(5), K2–S2, 3.202(2); K2–S3, 3.221(3); K2–S4, 3.236(3); K2–O2, 2.935(5), C1–S1, 1.660(9); C1–O1, 1.351(9); C1–N1, 1.327(9); N1–C2, 1.324(9); C2–S2, 1.670(8), C2–O2, 1.364(8); O3–C3, 1.351(9), C3–S3, 1.690(7); C3–N2, 1.318(9); N2–C4; 1.291(1), C4–S4, 1.708(9); C4–O4, 1.351(9); C1–N1–C2–S2, 52.4­(12).

K­[**3**] crystallizes in the noncentrosymmetric orthorhombic space group *Pna*2_1_ (*a* = 8.7251(5) Å, *b* = 23.397(2) Å, *c* = 12.3556(8) Å) with two independent molecules in the asymmetric unit and eight molecules in the unit cell (*Z* = 8). The bond lengths of K–S are in the range of 3.133(3)–3.276(3) Å, and no consistent trend in the bond lengths between nonbridging and bridging atoms is observed. The C–S bond lengths lie between 1.660(9) and 1.708(9) Å. Interestingly, the differences in C–S bond lengths containing bridging and nonbridging sulfur atoms are not consistent. However, in one [**3**]^−^ anion (Figure 4, right side), the C–S bond lengths are slightly longer (1.690(7) and 1.708(9) Å) than in the other [**3**]^−^ anion (Figure 4, left side, 1.660(9) and 1.670(8) Å). Consequently, the C3(4)–N2 bond lengths (1.318(9) Å, 1.291(1) Å) are shorter than the C1(2)–N1 bond lengths (1.327(9) Å, 1.324(9) Å). The C–O bond lengths are in the range of 1.351(9) to 1.365(8) Å and do not show any difference.

### Syntheses and Crystal Structures of Ni[1]_2_ and Ni­[**3**]_2_

In order to discuss commonalities and differences of bis­(imido-*C*,*C’*-dithiodicarbonic-*O*,*O*’-dialkyl ester)nickel complexes, the series of Ni complexes was completed by the synthesis of Ni­[**1**]_2_ and Ni­[**3**]_2_. Synthesizing the nickel complexes by salt metathesis via Li­[**2**] and K­[**3**] seemed logical but unnecessary in this case, since the previously published synthetic approach of nickel complex Ni­[**2**]_2_ is convenient. Therefore, we carried out analogous reactions toward Ni­[**1**]_2_ and Ni­[**3**]_2_. A mixture of anhydrous nickel­(II) chloride and the respective ligand in methanol or ethanol was treated with pyridine, leading to the formation of Ni­[**1**]_2_ and Ni­[**3**]_2_ as brown precipitates. The bench-stable complexes were obtained in moderate yields (Ni­[**1**]_2_, 59%; Ni­[**3**]_2_, 72%). Single crystals suitable for single crystal X-ray diffraction were grown by slow evaporation of the solvent from a saturated solution of the complexes in acetonitrile. The crystal structure of Ni­[**1**]_2_ is shown in Figure 5.

**Figure 5:**
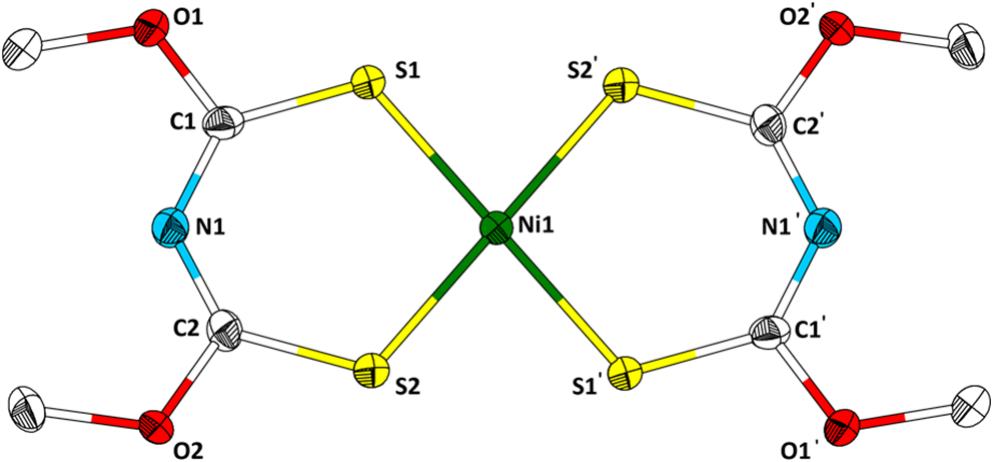
Structure of Ni­[**1**]_2_ in the single crystal. Ellipsoids are drawn with a 50% probability level. Hydrogen atoms are omitted for clarity. Selected bond lengths (Å) and angles (°): C1–O1, 1.337(4); S1–Ni1, 2.1750(8); S2–Ni1, 2.1646(8); C1–S1, 1.689(3); C1–N1, 1.320(4); N1–C2, 1.309(4); C2–S2, 1.701(3); C2–O2, 1.334(4).

Ni­[**1**]_2_ crystallizes in the monoclinic system with space group *P*2_1_/*c* (*a* = 3.9708(4) Å, *b* = 11.1573(10) Å, *c* = 15.6482(13) Å, β = 95.966(7)°) with two molecules in the unit cell (*Z* = 2) and one independent molecule in the asymmetric unit. The molecular structure of Ni­[**1**]_2_ is similar to the one of previously published Ni­[**2**]_2_.^14^ The nickel center is coordinated by four sulfur atoms of two ligands in a slightly distorted square planar fashion. The ligand backbone of Ni­[**1**]_2_ is slightly tilted; for a detailed discussion, see below (Figures 9 and 10). Ni–S distances are 2.1750(8) and 2.1646(8) Å, and the bite angle of ligand **1** is 97.17(3)°. Both are similar to the respective bond lengths and bite angles in the respective dithiobiuret complex (2.160(2) Å, 2.171(2) Å), and 96.09°.^12^ The C–S bond lengths are 1.689(3) and 1.701(3) Å and are slightly longer compared to its neutral free form (**1**, 1.657(2) Å, 1.648(2) Å). The opposite observation can be made by comparing the C–N bonds of the nickel complexes Ni­[**1**]_2_ (1.320(4) Å, 1.309(4) Å) to its free ligand **1** (both 1.377(3) Å). However, C–O bond lengths (1.337(4) Å, 1.334(4) Å) of Ni­[**1**]_2_ are larger than in **1** (1.303(3) Å, 1.311(3) Å): The shortening (enlargement) of C–N (C–S) bond lengths indicates an electron delocalization along the S–C–N–C–S moiety. A comparison of the bond lengths of dithiobiuret and its nickel complex reveals a similar trend. It is noteworthy that the shortening (enlargement) of C–N (C–S) bond lengths of ligands **1**–**3** and their complexes is more prominent than in dithiobiuret and its complexes.^12,13^ The reason for that might be stronger electron-donating properties of the −NH_2_ moiety in comparison to −O*R* (R = Me, Et, and ^*i*^Pr). Nevertheless, this comparison should be treated with caution because of strong hydrogen bonding within the crystal structure of the dithiobiuret nickel complexes.^12^ All discussed trends are in accordance with the discussion of Ni­[**2**]_2_ and its free ligand; the only significant difference is that the distortion of Ni­[**1**]_2_ is weaker than that of Ni­[**2**]_2_; see below for discussion (Figures 9 and 10).

Ni­[**3**]_2_ crystallizes in the triclinic space group *P*1̅ (*a* = 7.4842(6) Å, *b* = 9.0139(7) Å, *c* = 9.2050(8) Å, α = 66.960(6)°, β = 87.255(7)°, γ = 75.168(6)°) with one molecule in the unit cell (*Z* = 1, Figure 6).

**Figure 6:**
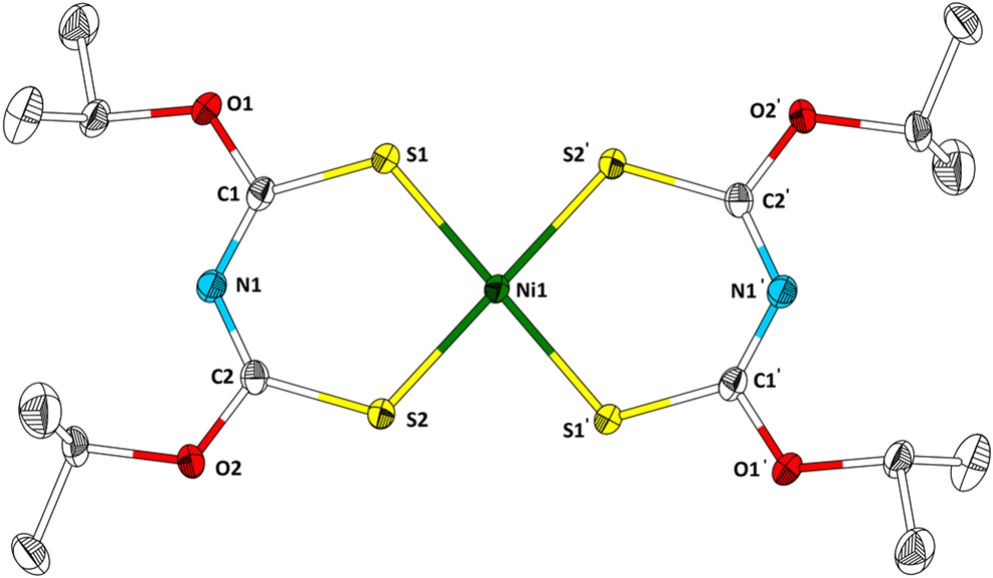
Structure of Ni­[**3**]_2_ in the single crystal. Ellipsoids are drawn with a 50% probability level. Hydrogen atoms are omitted for clarity. Selected bond lengths (Å) and angles (°): C1–O1, 1.321(2); S1–Ni1, 2.1597(5); S2–Ni1, 2.1548(5); C1–S1, 1.704(2); C1–N1, 1.319(2); N1–C2, 1.316(2); C2–S2, 1.701(2); C2–O2, 1.329(2).

A bond length comparison of Ni­[**3**]_2_ and **3** reveals the trend discussed earlier for Ni­[**1**]_2_ and **1**; see above. The C–N bond lengths of Ni­[**3**]_2_ shorten in comparison to its ligand **3**, and in contrast, the C–S bond lengths become longer, underlining the electron delocalization along the S–C–N–C–S moiety.

### Syntheses and Crystal Structures of Pd[1–3]_2_ and Pt­[**1–3**]_2_

With the nickel complexes in hand, it seems that our ligands **1**–**3** are suited for the synthesis of square planar coordinated metal centers. Therefore, we completed the group 10 metal series to discuss differences and similarities. We tested the reactivity of neutral **1**–**3** toward palladium­(II) and platinum­(II) salts prior to using the salt elimination approach via Li­[**2**] and K­[**3**]. For the palladium complexes, we used Pd­[OAc]_2_, since it is a common Pd^2+^ source that is soluble in coordinating solvents. Furthermore, [OAc]^−^ is a weak base, which makes the use of an additional base (like pyridine in the case of nickel complexes Ni­[**1**–**3**]_2_) unnecessary. It turned out that the base strength of [OAc]^−^ in acetonitrile solution is sufficient for abstracting the proton of **1**–**3**, and we obtained Pd­[**1**–**3**] in moderate (Pd­[**1**]_2_, 53%; Pd­[**2**]_2_, 63%) to good yields (Pd­[**3**]_2_, 82%). For the synthesis of the analogous platinum complexes, we used PtCl_2_ and the respective ligand in acetonitrile (Scheme 3).

**Scheme 3:**
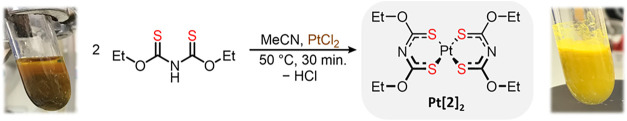
Suspension of Brown PtCl_2_ with Dissolved Ligand **2** in Acetonitrile (left)^a^

a Bright yellow suspension of Pt­[**2**]_2_ after completion of the reaction (right).

Compounds Pd­[**1**–**3**]_2_ and Pt­[**1**–**3**]_2_ are bench-stable bright yellow solids. Single crystals suitable for single crystal X-ray diffraction were obtained by slow evaporation of the solvent from a saturated solution of the complexes in acetonitrile. Pd­[**1**]_2_ and Pt­[**1**]_2_ crystallize isotypically to Ni­[**1**]_2_ (Figure 5). Pd­[**2**]_2_ does not crystallize isotopically to its analogous nickel complex Ni­[**2**]_2_. Instead, Pd­[**2**]_2_ crystallizes in the noncentrosymmetric orthorhombic system with space group *Pna*2_1_ (*a* = 14.8487(2) Å, *b* = 7.2233(1) Å, *c* = 17.3180(3) Å) with one molecule in the asymmetric unit and four units in the unit cell (*Z* = 4) (Figure 7).

**Figure 7:**
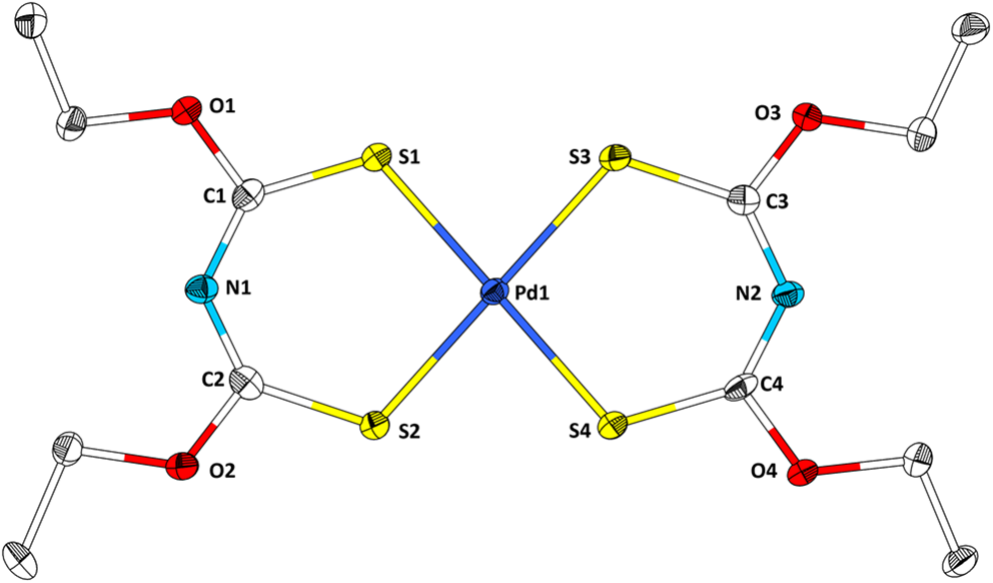
Structure of Pd­[**2**]_2_ in the single crystal. Ellipsoids are drawn with a 50% probability level. Hydrogen atoms are omitted for clarity. Selected bond lengths (Å) and angles (°): S1–Pd1, 2.288(2); S2–Pd1, 2.284(2); S3–Pd1, 2.287(2); S4–Pd1, 1.285(2); O1–C1, 1.327(1); C1–S1, 1.705(8); C1–N1, 1.329(1), N1–C2, 1.313(9); C2–S2, 1.713(8); C2–O2, 1.331(9); C3–O3, 1.323(9); C3–S3, 1.710(7); C3–N2, 1.320(9); N2–C4, 1.318(9); C4–S4, 1.707(8); C4–O4, 1.337(9), S1–Pd1–S2, 95.69(7).

Pd­[**3**]_2_ and Pt­[**3**]_2_ do not crystallize isotopically to their respective nickel congener. However, they crystallize isotypically in comparison to each other. Pd­[**3**]_2_ and Pt­[**3**]_2_ crystallize in the monoclinic space group *P*2_1_/*n* (Pd­[**3**]_2_, *a* = 7.3323(3) Å, *b* = 9.0815(4) Å, *c* = 16.9684(8) Å, β = 100.753(4)°; Pt­[**3**]_2_, *a* = 7.3445(3) Å, *b* = 9.1008(2) Å, *c* = 16.8609(7) Å, β = 100.937(3)°) with two molecules in the unit cell (*Z* = 2) and one independent molecule in the asymmetric unit (Figure 8).

**Figure 8:**
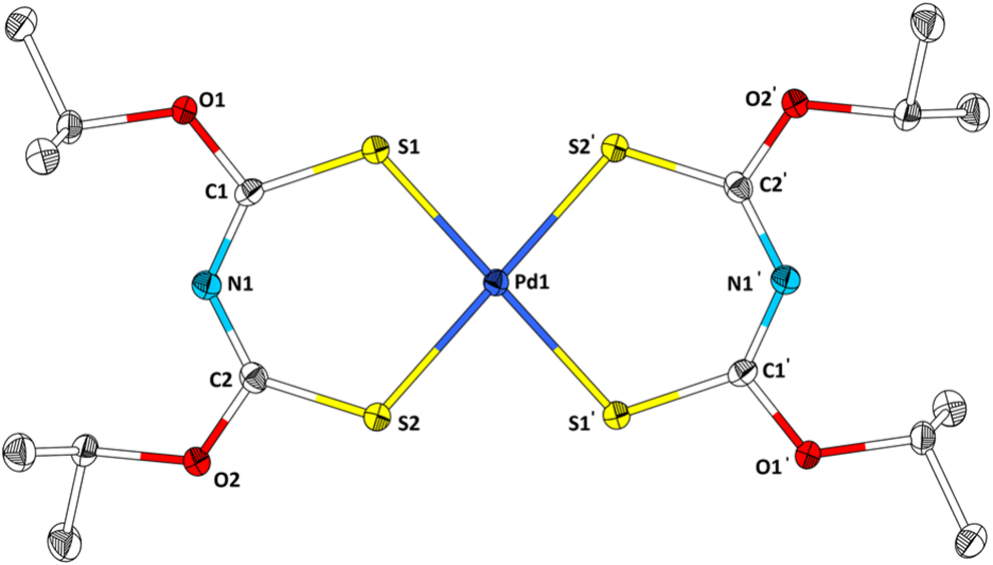
Structure of Pd­[**3**]_2_ in the single crystal; Pt­[**3**]_2_ crystallizes isotypically. Ellipsoids are drawn with a 50% probability level. Hydrogen atoms are omitted for clarity. Selected bond lengths (Å) and angles (°): S1–Pd1, 2.2738(5), S2–Pd1, 2.2804(5); C1–O1, 1.330(2); C1–S1, 1.697(2); C1–N1, 1.314(3); N1–C2, 1.316(3); C2–S2, 1.704(2); C2–O2, 1.327(3).

### Comparative Discussion

Ni­[**1**–**3**]_2_, Pd­[**1**–**3**]_2_, and Pt­[**1**–**3**]_2_ are bench-stable solids and are obtained in moderate to good yields (see SI Table S5). Their solubility depends on the aliphatic size of the ligand backbone: With increasing size of the aliphatic backbone of the ligand, the solubility increases. With regard to the synthesis, it is noticeable that with increasing size of group 10 metal, the affinity for ligand is increasing, which is consistent with the HSAB-concept.^22^ The reaction of NiCl_2_ with **1**–**3** in ethanol suspension without the addition of an additional base does not lead to the formation of Ni­[**1**–**3**]_2_, whereas the reaction of PtCl_2_ with **1**–**3** in acetonitrile leads to the formation of Pt­[**1**–**3**]_2_. To exclude a possible effect of the solvent, we performed the cross-experiment: We observed no reaction of NiCl_2_ with **1**–**3** in acetonitrile without the addition of bases. Quantitative ultraviolet–visible (UV–vis) spectra of the compounds in discussion reveal absorption maxima in the range of 277–420 nm (for detailed information about the UV–vis spectra, see SI Figures S42–S44 and Table S1).

In the solid state, the structural motifs of all complexes closely resemble each other. The metal centers are coordinated by four sulfur atoms of two ligands in a slightly distorted square planar fashion. On the one hand, complexation of group 10 metals Ni^2+^, Pd^2+^, and Pt^2+^ with **1**–**3** leads to the aforementioned change in binding situation of the ligand, manifesting itself in differences in bond lengths. C–S bond lengths are elongated by ∼0.05 Å from ∼1.65 Å in the free ligand to ∼1.70 Å in the complexes. C–N bond lengths, on the other hand, shorten by ∼0.05 Å from ∼1.37 Å in the ligand to ∼1.31 Å in *M*[**1**–**3**]_2_ (*M* = Ni^2+^, Pd^2+^, Pt^2+^). Furthermore, C–O bond lengths elongate slightly, ∼0.02 Å from 1.31 Å in the ligands to ∼1.33 Å in the complexes. As expected, S–*M* bond lengths increase from Ni^2+^ (∼2.16 Å) to Pd^2+^ and Pt^2+^ (both ∼2.28 Å), which is in accordance with the theoretical value for the respective S–*M* bond lengths.^21^ (See SI Table S4 for all relevant bond lengths and angles). The molecular structures of all of the complexes are not planar. For a description of the tilting angles observed for the ligand backbone, we compare angle Φ between the planes spanned by the ligand backbone S–C–N–C–S and the respective sulfur metal plane. This is illustrated in Figure 9 on the example of Ni­[**1**]_2_. In all complexes, this angle is nonzero and ranges from 6.6° in Ni­[**1**]_2_ to 19.5° in Ni­[**3**]_2_.

**Figure 9:**
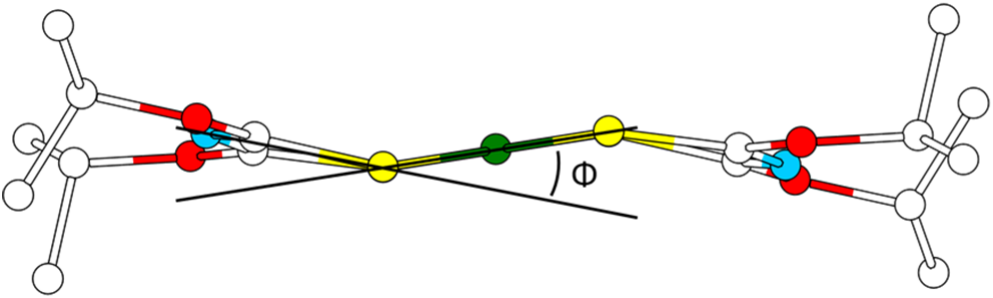
Side view of the structure of Ni[3]_2_. Tilting is indicated by the angle Φ of planes S–C–N–C–S and S–Ni–S. Atoms are shown with an arbitrary radius. Hydrogen atoms are omitted for clarity. Color code: green nickel, light blue nitrogen, white carbon, yellow sulfur, and red oxygen.

A comparison of Φ in Ni­[**1**–**3**]_2_, Pd­[**1**–**3**]_2_, and Pt­[**1**–**3**]_2_ is shown in Figure 10. For *M*[**1**]_2_ (*M* ^2+^ = Ni^2+^, Pd^2+^, Pt^2+^), the distortion increases with increasing size of the central atom. In contrast, this trend does not hold true for the complexes of **2** and **3**. Here, the distortion is larger in general, and a clear trend is absent.

**Figure 10:**
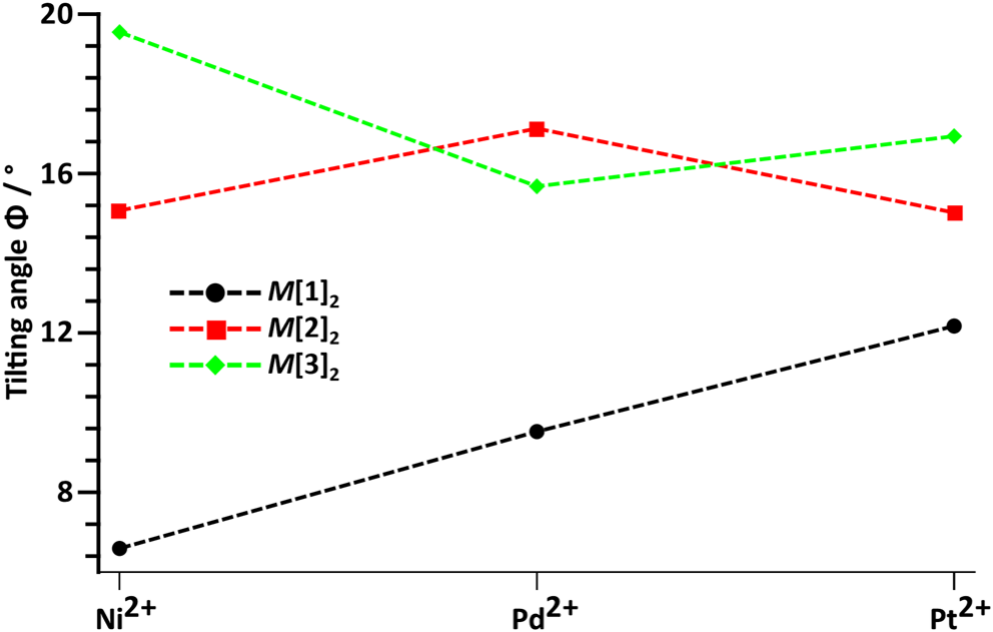
Tilting angle Φ of complexes M­[**1**–**3**]_2_ (*M* ^2+^ = Ni^2+^, Pd^2+^, Pt^2+^).

It appears that the steric influence of the ligand on Φ is the biggest for Ni­[**1**–**3**]_2_ (6.6–19.6°) and becomes less significant for Pd­[**1**–**3**]_2_ (9.5–17.1°) and Pt­[**1**–**3**]_2_ (12.2–17.0°). Discontinuities could potentially be attributed to crystal packing effects as the structures are, in part, non-isotypical.

### NMR Studies

^1^H- and ^13^C NMR spectra were collected for all of the compounds in question. In the ^1^H NMR spectrum of **1**, the two magnetically equivalent methyl groups are detected at 4.11 ppm and the imido moiety at 9.33 ppm (N*H*). In the spectra of the analogous *iso-*propyl compound **3**, the *iso-*propyl hydrogen atoms can be found at chemical shifts of 1.39 ppm of (CH­(C*H*_3_)_2_) and 5.52 ppm of (C*H*(CH_3_)_2_). The imido moiety (N*H*) is detected at 9.18 ppm. These shifts are in accordance with literature data^15^ except for the N*H* shift for the methyl compound, which can be attributed to the use of acetone-d^6^ in the literature as NMR solvent. The ^13^C NMR spectra of compounds **1**–**3** are shown in Figure 11.

**Figure 11:**
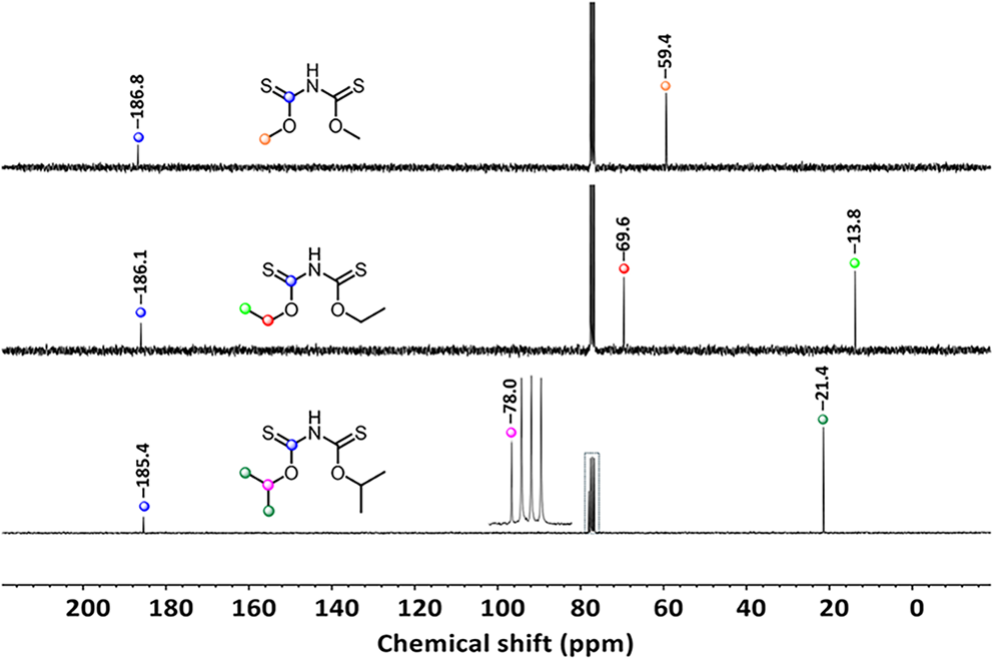
^13^C NMR spectra of **1**, **2** ^14^, and **3**.

The CH_3_ moiety of **1** is detected at 59.4 ppm and the *C*=S moiety at 186.8 ppm. In the ^13^C NMR spectrum of **3**, three signals are detected: 21.4 ppm (CH­(*C*H_3_)_2_), 78.0 ppm (*C*H­(CH_3_)_2_), and 185.4 ppm (*C*S). In the series **1**–**3**, a small high field shift of the *C*S moiety with increasing size of the alkyl rest is observed. The ^1^H NMR spectrum of Na­[**3**] and K­[**3**] with the *iso*-propyl group is detected at 1.22 ppm (1.22 ppm) and 5.33 ppm (5.30 ppm), and therefore, is almost identical. The same similarity can be found in the ^13^C NMR: Signals are observed at 22.4 (22.4 ppm), 72.9 (72.5 ppm), and 192.6 (191.5 ppm) for Na­[**3**] and K­[**3**], respectively. Compared to its neutral compound, the signal of the *iso*-propyl moieties is shifted toward the high field. A contrary observation is found for the *C*=S moiety: in Na­[**3**] (192.6 ppm) and K­[**3**] (191.5 ppm), it is shifted more downfield than the neutral compound (185.4 ppm).

In the ^13^C NMR spectra of Ni­[**1**]_2_, Pd­[**1**]_2_, and Pt­[**1**]_2_, the methyl group is detected at almost identical shifts at approximately 59 ppm, which is similar to the free ligand **1** (59.4 ppm). In contrast, the chemical shift of the *C*S moiety differs significantly between the complexes *M*[**1**]_2_ (Figure 12) and its free ligand **1**. The shift of *C*S in Ni­[**1**]_2_ (207.6 ppm) shows a large difference to its neutral ligand **1** (186.8 ppm). The respective palladium complex Pd­[**1**]_2_ shows the signal at 191.0 ppm and the platinum complex at 186.3 ppm, which is almost identical to the chemical shift of **1** (186.8 ppm). By comparing the *C*S shifts of Ni­[**1**]_2_, Pd­[**1**]_2_, and Pt­[**1**]_2_, it can be observed that with increasing size of the metal center, a shift toward high field is observed.

**Figure 12:**
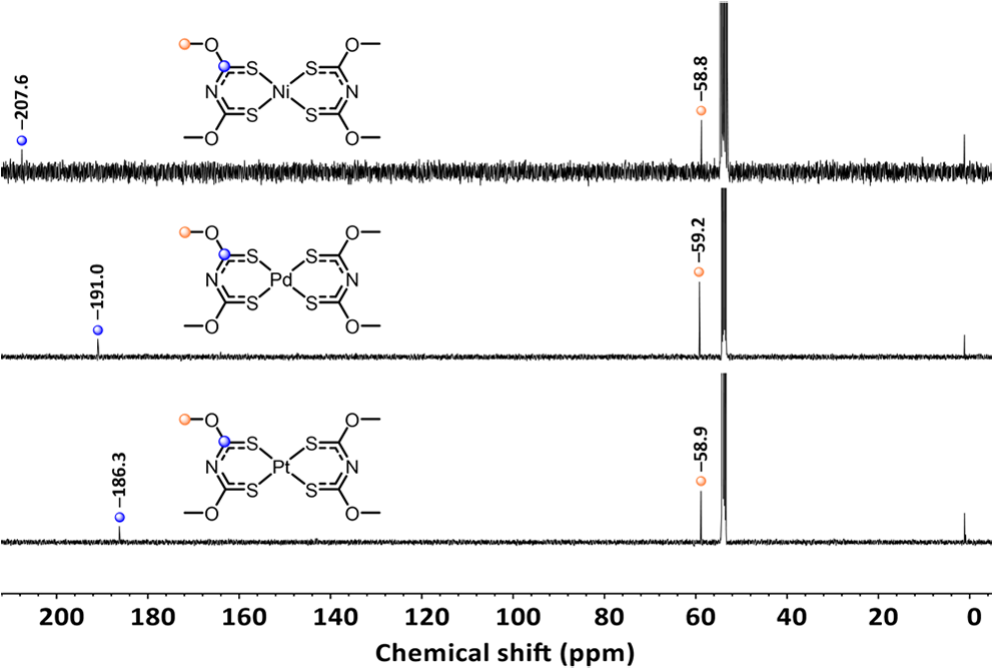
^13^C NMR spectra of Ni­[**1**]_2,_ Pd­[**1**]_2_, and Pt­[**1**]_2_.

In the ^13^C NMR spectra of Ni­[**2**]_2_, Pd­[**2**]_2_, and Pt­[**2**]_2_, the ethyl group is detected at almost identical shifts at approximately 69 and 14 ppm (Figure 13). These chemical shifts are similar to those of **2** (13.8 and 69.6 ppm). The same observation is made by a comparison of the ethyl group in ^1^H NMR (see the SI). However, the CS moiety does not conform to this similarity.

**Figure 13:**
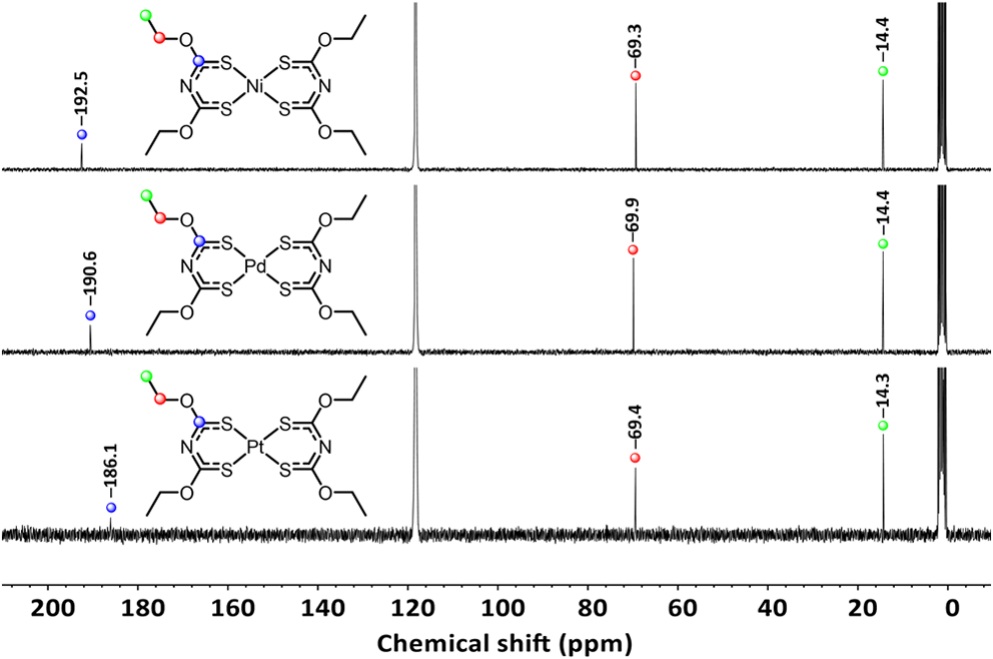
^13^C NMR spectra of Ni­[**2**]_2,_ Pd­[**2**]_2_, and Pt­[**2**]_2_.

Highfield shift trend is observed from Ni­[**2**]_2_ (192.5 ppm), Pd­[**2**]_2_ (190.6 ppm) to Pt­[**2**]_2_ (186.1 ppm). Only the chemical shift of the *C*S moiety of Pt­[**2**]_2_ represents similarity to its neutral ligand **2** (186.1 ppm).

In the ^1^H- and ^13^C NMR spectra of Ni­[**3**]_2_, Pd­[**3**]_2_, and Pt­[**3**]_2_, the previously discussed trends are observed, too. The *iso*-propyl groups are detected at almost identical shifts at approximately 22 and 77 ppm. Compared to its neutral compound **3** (21 and 78.0 ppm), only tiny differences can be observed. The *C*S moiety does not conform to this similarity and is more influenced by the metal center (Figure 14).

**Figure 14:**
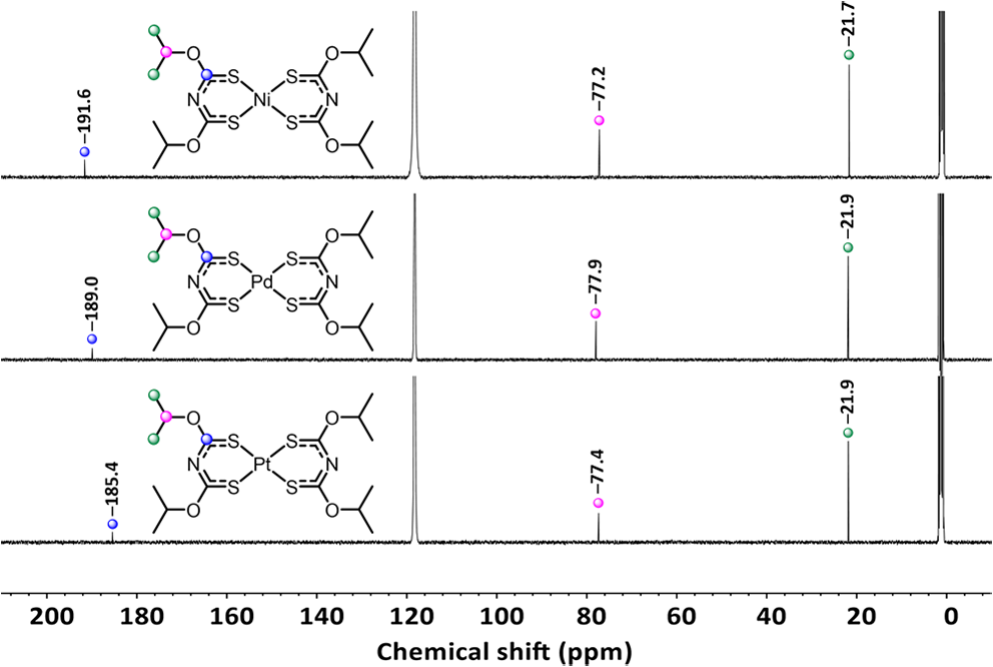
^13^C NMR spectra of Ni­[**3**]_2_, Pd­[**3**]_2_, and Pt­[**3**]_2_.

Similarly to complexes **1** and **2**, a high field shift is observed from Ni­[**3**]_2_ (191.6 ppm), Pd­[**3**]_2_ (189.0 ppm) to Pt­[**3**]_2_ (185.4 ppm). As before, the chemical shift of the C=S moiety of Pt­[**3**]_2_ (185.4 ppm) represents similarity to the shift in **3** (185.4 ppm).

In summary, two observations can be made. The chemical shifts of the *C*S moieties in the ^13^C NMR spectra of the discussed complexes are in the range of 185.4 ppm (Pt­[**3**]_2_) and 207.6 ppm (Ni­[**1**]_2_). These are predominantly influenced by the metal center and weakly by the organic residue of the respective ligand. The heavier the metal gets, the higher field shift the *C*=S moiety experiences. Spin–orbit heavy-atom effect on the light-atom (SO-HALA effect) can largely be excluded, since this effect is only non-negligible for directly neighboring atoms.^23^ Interestingly, a more electron rich organic backbone of the ligand manifests itself in more high field shifted *C*S signals in the case of the nickel complexes 207.6 ppm for Ni­[**1**]_2_ and 191.6 ppm for Ni­[**3**]_2_; however, this effect is almost negligible for Pd­[**1**–**3**]_2_ and Pt­[**1**–**3**]_2_ (Figure 15).

**Figure 15:**
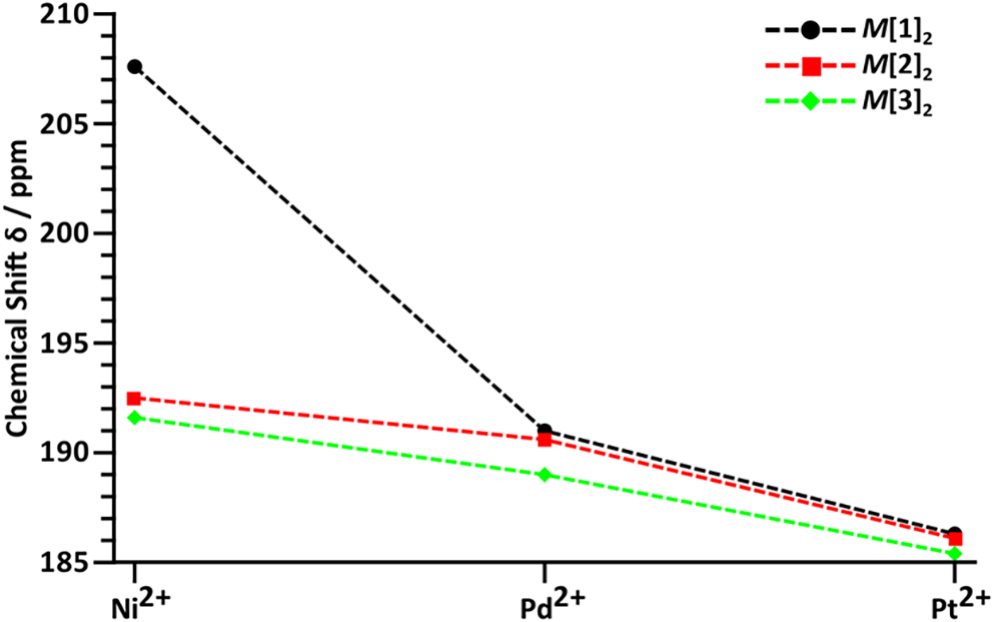
^13^C NMR shifts of CS moiety in complexes Ni­[**1**–**3**]_2_,^14^ Pd­[**1**–**3**]_2_, and Pt­[**1**–**3**]_2._.

## Conclusion

In this work, we present a series of group 10 metal complexes of imido-*C*,*C’*-dithiodicarbonic-*O*,*O*’-dialkyl ester (ligands **1** = Me, **2** = Et, **3** = ^*i*^Pr; and complexes Ni­[**1**–**3**]_2_, Pd­[**1**–**3**]_2_, and Pt­[**1**–**3**]_2_), and discuss their syntheses, structural relations, and NMR signatures. These monoanionic chelate ligands form two six-membered thiolate–thione rings, complexing the respective metal center in square planar geometry. A comparison of the bond lengths in the free ligands 1–3 and their complexes M­[**1**–**3**]_2_ (M = Ni^2+^, Pd^2+^, Pt^2+^) reveals electron delocalization along the S–C–N–C–S backbone, evidenced by a lengthening of the C–S bonds and a shortening of the C–N bonds upon coordination. Despite the complexation, significant tilting of the S–C–N–C–S–M moiety is observed. The angle Φ of planes S–C–N–C–S and S–M–S increases with the steric demand of the respective ligand. This effect is most clearly observed in the nickel complexes (span of Φ of Ni­[**1**]_2_ = 6.6 and Ni­[**3**]_2_ = 19.6°) and weaker in Pd­[**1**–**3**]_2_ and Pt­[**1**–**3**]_2_. Here, it seems that with increasing size of the metal center, the steric influence of the ligand on Φ becomes less significant. ^13^C NMR studies reveal shifts of 185.4–186.8 ppm of the CS moiety for the ligands **1**–**3** and shifts of 185.4 ppm (Pt­[**3**]_2_) up to 207.6 ppm (Ni­[**1**]_2_) for the complexes. By comparison of ^13^C NMR shifts of the CS moiety within the series of complexes *M*[**1**–**3**]_2_ (M = Ni^2+^, Pd^2+^, Pt^2+^), two trends are observed. With the increasing size of the metal center, the respective δ (*C*S) is shifted stronger toward high field. More electron-rich substituents also lead to high field shifted δ (*C*S). This effect is significant for the nickel complexes; however, for palladium and platinum complexes, this effect is modest.

## Experimental Section

### Synthesis of **1**

Thiocarbonyl dithiocyanate (0.80 g, 4.99 mmol, 1.00 equiv) was dissolved in dichloromethane (5 mL) and cooled to – 50 °C (cooling leads to precipitation of the thiocarbonyl dithiocyanate). Methanol (5.00 mL, 123.28 mmol, 50.00 equiv) was added dropwise into the orange suspension. After complete addition, the cooling bath was removed, and the reaction mixture was allowed to warm to room temperature. After 18 h of stirring at room temperature, all volatiles were removed *in vacuo*. The yellowish residue was then dissolved in dichloromethane (5 mL) and filtered. All volatiles of the orange solution were removed under reduced pressure. The product (172.00 mg, 1.04 mmol, 21%) was obtained as a faint yellow solid. Single crystals suitable for single crystal X-ray diffraction were obtained by slowly evaporating the solvent of a saturated solution in *n*-hexane. ^**1**^ **H NMR** (CDCl_3_) δ/ppm: 9.33 (s, 1H, N*H*), 4.11 (s, 6H, OC*H*_3_). ^**13**^ **C NMR** (CDCl_3_) δ/ppm: 69.6 (O*C*H_3_), 186.8 (*C*=S). **CHNS** (calc/found) C: 29.08/28.60, H: 4.27/4.116, N: 8.48/9.23, S: 38.81/42.339. (Due to the high sulfur content weight%, the calibration value for S was overshot; the values are not reliable.) **Mass** (ESI+, MeOH) for [M + H^+^] = C_4_H_7_N_1_O_2_S_2_H^+^ *m*/*z* found (calcd): 165.9987 (165.9991).

### Synthesis of **3**

Thiocarbonyl dithiocyanate (0.35 g, 2.18 mmol, 1.00 equiv) was dissolved in dichloromethane (5 mL) and cooled to −50 °C (cooling leads to precipitation of the thiocarbonyl dithiocyanate). Then, *iso*-propanol (5.00 mL, 64.88 mmol, 29.70 equiv) was added dropwise into the orange suspension. After complete addition, the cooling bath was removed, and the reaction mixture was allowed to warm to room temperature. After 18 h of stirring at room temperature, all volatiles were removed *in vacuo*. The product (319.00 mg, 1.13 mmol, 66%) was obtained as an orange-red solid. Single crystals suitable for single crystal X-ray diffraction were obtained by slowly evaporating the solvent of a saturated solution in *n*-hexane. ^**1**^ **H NMR** (CDCl_3_) δ/ppm: 9.18 (s, 1H, N*H*), 5.52 (h, 2H, ^3^ *J*_HH_ = 6.15 Hz, OC*H*(CH_3_)_2_), 1.39 (t, 6H, ^3^ *J*_HH_ = 6.26 Hz, OCH­(C*H*_3_)_2_). ^**13**^ **C NMR** (CDCl_3_) δ/ppm: 185.4 (*C*=S), 78.0 (O*C*H­(CH_3_)_2_), 21.4 (OCH_2_ *C*H_3_). **CHNS** (calc/found) C: 43.41/42.99, H: 6.83/6.376, N: 6.33/6.92, S: 28.97/30.707. **Mass** (ESI+, MeOH) for [M+H^+^] = C_6_H_11_N_1_O_2_S_2_H^+^ *m*/*z* found (calcd) 193.0304 (193.0231).

### Synthesis of Na­[**3**]

NaHMDS (165.70 mg, 903.62 μmol, 1.00 equiv) was suspended in diethyl ether (10 mL) and cooled to 0 °C. A solution of **3** (200.00 mg, 903.62 μmol, 1.00 equiv) in diethyl ether (5 mL) was added dropwise, which led to the formation of a yellow-orange precipitate. The cooling bath was removed, and the reaction mixture was stirred for 2 h. Afterward, the solvent of the crude beige reaction mixture was removed *in vacuo*. The obtained beige solid was washed with *n*-hexane (2 × 7 mL) and subsequently dried *in vacuo*. Na­[**3**] is obtained as a beige solid (132.00 mg, 542.51 μmol, 60%). ^**1**^ **H NMR** (THF-d^8^) δ/ppm: 5.33 (septet, 2H, ^3^ *J*_HH_ = 6.27 Hz, OC*H*(CH_3_)_2_), 1.22 (t, 6H, ^3^ *J*_HH_ = 6.26 Hz, OCH­(C*H*_3_)_2_). ^**13**^ **C NMR** (THF-d^8^) δ/ppm: 192.6 (*C*=S), 72.9 (O*C*H­(CH_3_)_2_), 22.4 (OCH_2_ *C*H_3_). **CHNS** (calcd/found) C: 39.49/38.19, H: 5.80/5.73, N: 5.76/8.13, S: 26.35/24.43.

### Synthesis of K­[**3**]

KO^t^Bu (101.4 mg, 903.62 μmol, 1.00 equiv) was suspended in diethyl ether (5 mL) and cooled to −30 °C. A solution of **3** (200.00 mg, 903.62 μmol, 1.00 equiv) in diethyl ether (10 mL) was added dropwise, which led to the formation of a colorless precipitate. After being stirred for 30 min, the precipitate was filtered off and dried *in vacuo*. The product (167.00 mg, 643.74 μmol, 71%) was obtained as a beige solid. Crystals suitable for single crystal X-ray diffraction were grown by slow diffusion of *n*-hexane into a concentrated solution of K­[**3**] in THF. ^**1**^ **H NMR** (THF-d^8^) δ/ppm: 5.30 (septet, 2H, ^3^ *J*_HH_ = 6.23 Hz, OC*H*(CH_3_)_2_), 1.22 (t, 6H, ^3^ *J*_HH_ = 6.26 Hz, OCH­(C*H*_3_)_2_). ^**13**^ **C NMR** (THF-d^8^) δ/ppm: 191.5 (*C*=S), 72.5 (O*C*H­(CH_3_)_2_), 22.4 (OCH_2_ *C*H_3_). **CHNS** (calcd/found) C: 37.04/37.16, H: 5.44/5.60, N: 5.40/5.70, S: 24.72/25.19.

### Synthesis of Ni­[**1**]_2_

NiCl_2_ (30.00 mg, 231.49 μmol, 1.00 equiv) and **1** (80.32 mg, 486.14 μmol, 2.10 equiv) were suspended in methanol (5 mL) at room temperature. After the addition of pyridine (39.49 μL, 486.14 mmol, 2.10 equiv), a brown precipitate formed. The brown suspension was stirred at room temperature for 1 day. The solvent was removed *in vacuo*, and the crude product was successively washed with methanol (1 mL) and *n*-pentane (3 mL) and dried *in vacuo*. The product (53.00 mg, 136.91 μmol, 59%) was obtained as a brown solid. Single crystals suitable for single crystal X-ray diffraction were obtained by slowly evaporating the solvent of a saturated solution in acetonitrile. ^**1**^ **H NMR** (THF-d^8^) δ/ppm: 3.96 (s, 12H) ^**13**^ **C NMR** (CD_2_Cl_2_) δ/ppm: 207.6 (*C*=S), 58.8 (O*C*H_3_). **CHNS** (calcd/found) C: 24.82/24.14, H: 3.12/3.113, N: 7.24/7.56, S: 33.13/34.507.

### Synthesis of Pd­[**1**]_2_

Pd­(OAc)_2_ (30.00 mg, 133.63 μmol, 1.00 equiv) was dissolved in acetonitrile (3 mL), and a solution of **1** (46.36 mg, 280.61 μmol, 2.10 equiv) in acetonitrile (6 mL) was added. The reaction mixture was stirred at room temperature for 3 days. Afterward, the solvent of the orange solution was removed *in vacuo*, and the yellow/orange solid was washed with *n*-pentane (3 × 3 mL). The product (31.00 mg, 71.29 μmol, 53%) was obtained as a yellow-orange solid. Single crystals suitable for single crystal X-ray diffraction were obtained by slowly evaporating the solvent of a saturated solution in acetonitrile. ^**1**^ **H NMR** (CD_2_Cl_2_) δ/ppm: 3.99 (s, 12H) ^**13**^ **C NMR** (CD_2_Cl_2_) δ/ppm: 191.0 (*C*=S), 59.2 (O*C*H_3_). **CHNS** (calc./found.) C: 22.10/23.41, H: 2.78/2.944, N: 6.44/9.52, S: 29.49/29.722. **Mass** (LIFDI+, THF) for [M^+^] = C_8_H_6_N_2_O_4_S_4_Pd^+^ *m*/*z* found (calcd) 433.87311 (433.87147).

### Synthesis of Pt­[**1**]_2_

PtCl_2_ (40.00 mg, 150.38 μmol, 1.00 equiv) was suspended in acetonitrile (3 mL), and a freshly filtered solution of **1** (62.12 mg, 375.96 μmol, 2.50 equiv) in 40 °C acetonitrile (6 mL) was added. The reaction mixture was stirred for 2 h at 40 °C and 7 h at room temperature. Afterward, the reaction mixture was cooled to – 20 °C and filtered. The yellow solid was washed with 0 °C cold methanol (5 mL) and *n*-pentane (5 mL) and dried *in vacuo*. The product (48.50 mg, 92.64 μmol, 62%) was obtained as a yellow solid. Single crystals suitable for single crystal X-ray diffraction were obtained by slowly evaporating the solvent of a saturated solution in acetonitrile. ^**1**^ **H NMR** (CD_2_Cl_2_) δ/ppm: 3.95 (s, 12H) ^**13**^ **C NMR** (CD_2_Cl_2_) δ/ppm: 186.3 (*C*=S), 58.9 (O*C*H_3_). **CHNS** (calcd/found) C: 18.35/18.56, H: 2.31/2.414, N: 5.35/6.67, S: 24.50/24.773.

### Synthesis of Pd­[**2**]_2_

Pd­(OAc)_2_ (30.00 mg, 133.63 μmol, 1.00 equiv) was dissolved in acetonitrile (5 mL), and a solution of **2** (51.65 mg, 267.25 μmol, 2.00 equiv) in acetonitrile (5 mL) was added. The brown reaction mixture was stirred at room temperature for 12 h. The obtained reaction mixture with yellow precipitate was cooled to −20 °C, and the liquid phase was separated by decantation. Afterward, the yellow solid was washed with diethyl ether (2 mL) and dried *in vacuo*. The product (41.00 mg, 83.51 μmol, 63%) was obtained as a yellow solid. Single crystals suitable for single crystal X-ray diffraction were obtained by slowly evaporating the solvent of a saturated solution in acetonitrile. ^**1**^ **H NMR** (CD_3_CN) δ/ppm = 4.43 (q, 4H, ^3^ *J*_HH_ = 7.10 Hz, OC*H*_2_CH_3_), 1.33 (t, 6H, ^3^ *J*_HH_ = 7.09 Hz, OCH_2_C*H*_3_). ^**13**^ **C NMR** (75 MHz, CD_3_CN, r.t.) δ/ppm = 190.6 (*C*=S), 69.9 (O*C*H_2_CH_3_), 14.4 (OCH_2_ *C*H_3_). **CHNS** (calc./found.) C: 29.36/30.94, H: 4.11/4.403, N, 5.71/6.45, S, 26.12/26.317. **Mass** (ESI+, CH_3_CN) for [M + H^+^] = C_12_H_20_N_2_O_4_S_4_PdH^+^ *m*/*z* found (calcd): 490.9409 (490.9416).

### Synthesis of Pt­[**2**]_2_

PtCl_2_ (60.00 mg, 225.58 μmol, 1.00 equiv) and **2** (87.20 mg, 451.15 μmol, 2.00 equiv) were suspended in acetonitrile (3 mL). The reaction mixture was stirred at 50 °C for 30 min. Afterward, the reaction mixture with yellow precipitate was cooled down to −20 °C, and the liquid phase was removed by decantation. The yellow precipitate was washed with −20 °C cold acetonitrile (3 mL) and diethyl ether (2 × 4 mL), and dried *in vacuo*. The product (78.00 mg, 134.57 μmol, 60%) was obtained as a yellow solid. Single crystals were grown by slowly evaporating the solvent of a saturated solution in acetonitrile. ^**1**^ **H NMR** (300 MHz, CD_3_CN, r.t.) δ/ppm = 4.40 (q, 4H, ^3^ *J*_HH_ = 7.08 Hz, OC*H*_2_CH_3_), 1.34 (t, 6H, ^3^ *J*_HH_ = 7.08 Hz, OCH_2_C*H*_3_). ^**13**^ **C NMR** (75 MHz, CD_3_CN, r.t.) δ/ppm = 186.1 (*C*=S), 69.4 (O*C*H_2_CH_3_), 14.3 (OCH_2_ *C*H_3_). **CHNS** (calcd/found) C: 24.87/24.93, H: 3.48/3.471, N: 4.83/5.27, S: 22.12/22.106. **Mass** (LIFDI, THF) for [M^+^] = C_12_H_20_N_2_O_4_S_4_Pt^+^ *m*/*z* found (calcd) 578.99585 (578.99536).

### Synthesis of Ni­[**3**]_2_

NiCl_2_ (23.00 mg, 177.48 μmol, 1.00 equiv) was suspended in ethanol (6 mL), and a freshly filtered solution of **3** (78.56 mg, 354.96 μmol, 2.00 equiv) in ethanol (6 mL) was added. Dropwise addition of pyridine (42.98 μL, 537.43 μmol, 3.00 equiv) led to the formation of a light brown precipitate. Afterward, the reaction mixture was stirred for 1 day at room temperature. After dilution with water (10 mL) and cooling to 0 °C, the brown precipitate was filtered off. The crude product was washed with methanol (5 mL) and then with *n*-pentane (5 mL) and subsequently dried *in vacuo*. The product (64.00 mg, 128.17 μmol, 72%) was obtained as a light brown powder. Single crystals were grown by the slow evaporation of the solvent of a saturated solution in acetonitrile. ^**1**^ **H NMR** (300 MHz, CD_3_CN, r.t.) δ/ppm = 5.35 (hept, 4H, ^3^ *J*_HH_ = 6.21 Hz, OC*H*(CH_3_)_2_), 1.33 (d, 24H, ^3^ *J*_HH_ = 6.23 Hz, OCH­(C*H*_3_)_2_) ^**13**^ **C NMR** (75 MHz, CD_3_CN, r.t.) δ/ppm = 191.6 (*C*=S), 77.2 (O*C*H­(CH_3_)_2_), 21.7 (OCH­(*C*H_3_)_2_). **CHNS** (calc./found.) C: 38.49/37.52, H: 5.496/5.479, N: 5.61/5.45, S: 25.68/25.499. **Mass** (LIFDI+, THF) for [M^+^] = C_16_H_28_N_2_O_4_S_4_Ni^+^ *m*/*z* found (calcd) 498.03029 (498.02854).

### Synthesis of Pd­[**3**]_2_

Pd­(OAc)_2_ (39.00 mg, 173.71 μmol, 1.00 equiv) was dissolved in acetonitrile (3 mL), and a filtered solution of **3** (96.12 mg, 434.28 μmol, 2.50 equiv) in acetonitrile (6 mL) was added. The reaction mixture immediately formed a yellow precipitate and was then stirred for 7 days at room temperature. Afterward, the suspension was cooled to −20 °C and filtered. The yellow solid was washed with *n*-pentane (2 × 10 mL) and dried *in vacuo*. The product (78.10 mg, 142.76 μmol, 82%) was obtained as a yellow powder. Single crystals were grown by slow evaporation of the solvent of a saturated solution in acetonitrile. ^**1**^ **H NMR** (500 MHz, CD_3_CN, r.t.) δ/ppm = 5.32 (hept, 4H, ^3^ *J*_HH_ = 6.24 Hz, OC*H*(CH_3_)_2_), 1.32 (d, 24H, ^3^ *J*_HH_ = 6.20 Hz, OCH­(C*H*_3_)_2_). ^**13**^ **C NMR** (75 MHz, CD_3_CN, r.t.) δ/ppm = 189.0 (*C*=S), 77.0 (O*C*H­(CH_3_)_2_), 21.1 (OCH­(*C*H_3_)_2_). **CHNS** (calc./found.) C: 35.13/35.07, H: 5.16/4.957, N: 5.61/5.45, S: 25.68/25.499. **Mass** (LIFDI+, THF) for [M^+^] = C_16_H_28_N_2_O_4_S_4_Pd^+^ *m*/*z* found (calcd): 545.99610 (545.99667).

### Synthesis of Pt­[**3**]_2_

PtCl_2_ (40.00 mg, 150.38 μmol, 1.00 equiv) was suspended in acetonitrile (3 mL), and a filtered solution of **3** (83.21 mg, 375.96 μmol, 2.50 equiv) in 40 °C warm acetonitrile (6 mL) was added. After stirring at 40 °C for 2 h, the yellow-brownish suspension was stirred for 2 days at room temperature. Then the suspension was cooled to −20 °C, filtered, and the solid was dried *in vacuo*. The product (54.00 mg, 84.94 μmol, 56%) was obtained as a yellow-brown powder. Single crystals were grown by slow evaporation of the solvent of a saturated solution in acetonitrile. **NMR** (500 MHz, CD_3_CN, r.t.) δ/ppm = 5.32 hept, 4H, ^3^ *J*_HH_ = 6.24 Hz, OC*H*(CH_3_)_2_, 1.32 (d, 24H, ^3^ *J*_HH_ = 6.20 Hz, OCH­(C*H*_3_)_2_). ^**13**^ **C NMR** (75 MHz, CD_3_CN, r.t.) δ/ppm = 189.0 (*C*=S), 77.0 (O*C*H­(CH_3_)_2_), 21.1 (OCH­(*C*H_3_)_2_). ^**195**^ **Pt NMR** (500 MHz, CD_3_CN, rt) δ/ppm = −3719.84. **CHNS** (calc./found.) C: 35.13/35.07, H: 5.16/4.957, N: 5.61/5.45, S: 25.68/25.499. **Mass** (LIFDI+, THF) for [M^+^] = C_16_H_28_N_2_O_4_S_4_Pt^+^ *m*/*z* found (calcd) 635.05999 (635.05796).

## Supplementary Material



## Notes

They contain further experimental details, NMR spectra, and X-ray crystallographic information (PDF)
