# Supplementary material for: Six-Membered Thiolate–Thione Chelate Complexes of Group 10 Elements—Syntheses, Crystal Structures, and NMR Studies
Source: ACS Omega. 2025 Oct 15;10(42):50153–62. doi: 10.1021/acsomega.5c06668 (PMC12573023; doi:10.1021/acsomega.5c06668)
Supplement: Supplementary file 1 [file ao5c06668_si_001.pdf]

Electronic Supporting Information (ESI)

Six-membered thiolate-thione chelate complexes of group 10 elements – Syntheses, crystal structures and NMR studies

Hennes Günther[a], Stephan Hohloch[b] and Frank Tambornino* [a]

[a]Fachbereich Chemie, Philipps-Universität Marburg, Hans-Meerwein-Straße 4, 35043 Marburg, Germany

[b]Universitiy of Innsbruck, Department of Coordination Chemistry, Innrain 80-82, A-6020 Innsbruck, Austria.

Contents

1. Experimental section 3
1.1 General remarks 3
1.2 Additional characterization techniques 3
2. NMR and IR spectra 4
2.1 Imido-*C*,*C'*-dithiodicarbonic-*O*,*O*'-dimethyl ester (1) 4
2.2 Imido-*C*,*C'*-dithiodicarbonic-*O*,*O*'-diisopropyl ester (3) 6
2.3 Sodium salt of imido-*C*,*C'*-dithiodicarbonic-*O*,*O*'-diisopropyl ester (Na[3]) 8
2.4 Potassium salt of imido-*C*,*C'*-dithiodicarbonic-*O*,*O*'-diisopropyl ester (K[3]) 10
2.5 Bis(imido-*C*,*C'*-dithiodicarbonic-*O*,*O*'-dimethyl ester)nickel(II) (Ni$[1]_2$) 12
2.6 Bis(imido-*C*,*C'*-dithiodicarbonic-*O*,*O*'-dimethyl ester)palladium(II) (Pd$[1]_2$) 14
2.7 Bis(imido-*C*,*C'*-dithiodicarbonic-*O*,*O*'-dimethyl ester)platinum(II) (Pt$[1]_2$) 16
2.8 Bis(imido-*C*,*C'*-dithiodicarbonic-*O*,*O*'-diethyl ester)palladium(II) (Pd$[2]_2$) 18
2.9 Bis(imido-*C*,*C'*-dithiodicarbonic-*O*,*O*'-diethyl ester)platinum(II) (Pt$[2]_2$) 19
2.10 Bis(imido-*C*,*C'*-dithiodicarbonic-*O*,*O*'-diisopropyl ester)nickel(II) (Ni$[3]_2$) 21
2.11 Bis(imido-*C*,*C'*-dithiodicarbonic-*O*,*O*'-diisopropyl ester)palladium(II) (Pd$[3]_2$) 23
2.12 Bis(imido-*C*,*C'*-dithiodicarbonic-*O*,*O*'-diisopropyl ester)platinum(II) (Pt$[3]_2$) 25
2.13 Unsuccessful reactions of thiocarbonyl dithiocyanate with selected alcohols 27
3 Quantitative UV-Vis spectra 30
4 Crystallographic details 32
4.1 Additional pictures of crystal structures 35
4.2 Additional tables 40
4. Literature 41

1. Experimental section

1.1 General remarks

General synthetic methods. All reactions and manipulations were performed under an inert atmosphere of argon using standard Schlenk-line or glovebox techniques (MBraun UNIlab glovebox, maintained at < 0.1 ppm H_2O and < 0.1 ppm O_2). Solvents were dried according to literature.[1]

Nickel(II) chloride (Sigma-Aldrich, 98%), palladium(II) acetate (Sigma-Aldrich, 99%) and platinum(II) chloride (abcr, 99.9%) were used as received.

Pyridine (Fisher Scientific, Certified AR, for analysis), CD_3CN (Eurisotop, 99.8 %), THF-d_8 (Eurisotop, 99.5 %) CD_2Cl_2 (Eurisotop, 99.8 %) were degassed and stored over molecular sieve (3 Å) prior to use.

1.2 Additional characterization techniques

^1H and ^{13}C NMR spectra were acquired on a Bruker Avance II (300 MHz) spectrometer at 298 K if not stated otherwise.

Elemental analyses and mass spectrometry were performed by the in-house service personnel. CHN(S) analyses were performed on a *CHNS(S)-Analysator vario MICRO CUBE* (Elementar). Samples were prepared and sealed under inert conditions in a tin crucible. Electro spray ionization (ESI) was performed on a *LTQ-FT Ultra* (Thermo Fischer Scientific) and liquid injection field desorption ionization (LIFDI) was performed on an AccuTOF GCv (Jeol).

IR spectra were recorded on a Bruker Alpha FT-IR spectrometer equipped with a diamond ATR unit.

2. NMR and IR spectra

2.1 Imido-*C*,*C'*-dithiodicarbonic-*O*,*O'*-dimethyl ester (1)

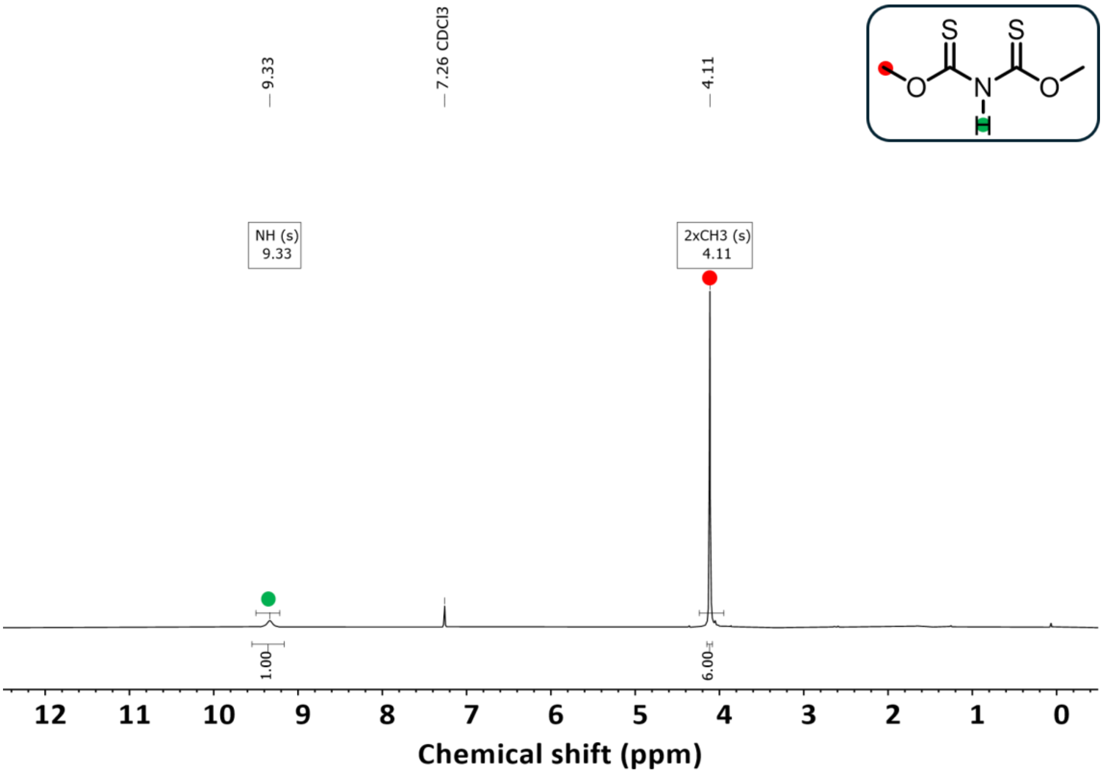

Figure S 1: ^{1}H-NMR spectrum of **1** in $CDCl_3$ at room temperature.

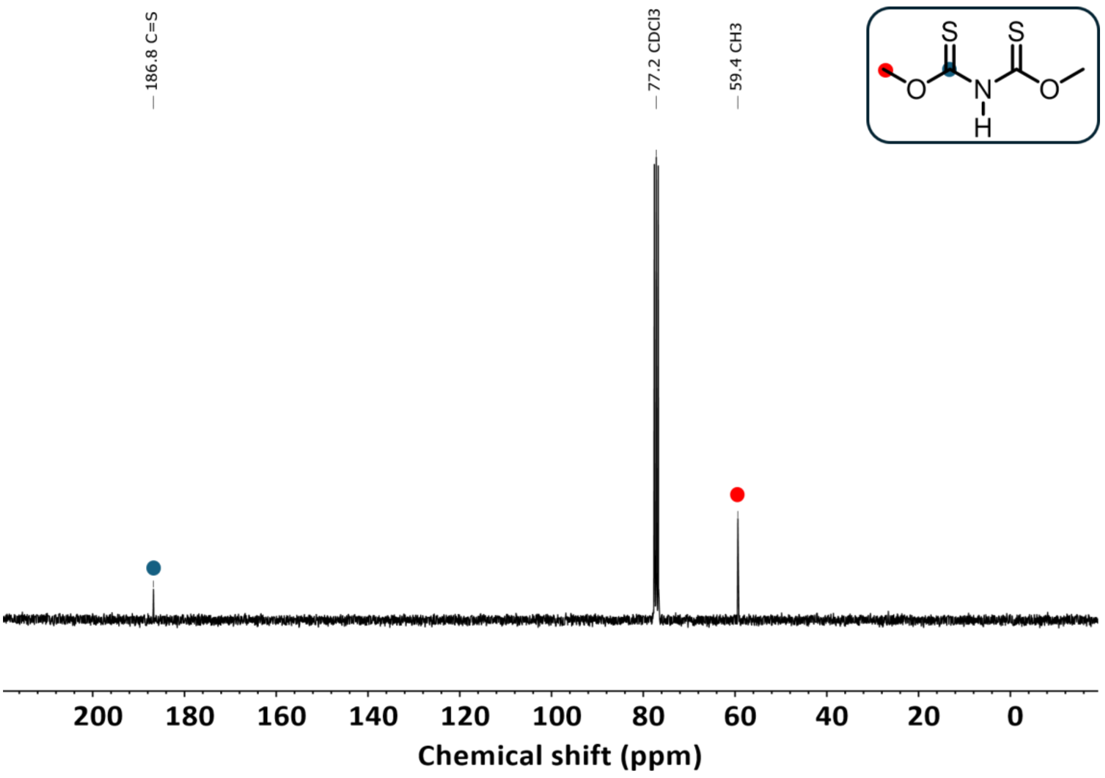

Figure S 2: ^{13}C-NMR spectrum of **1** in $CDCl_3$ at room temperature.

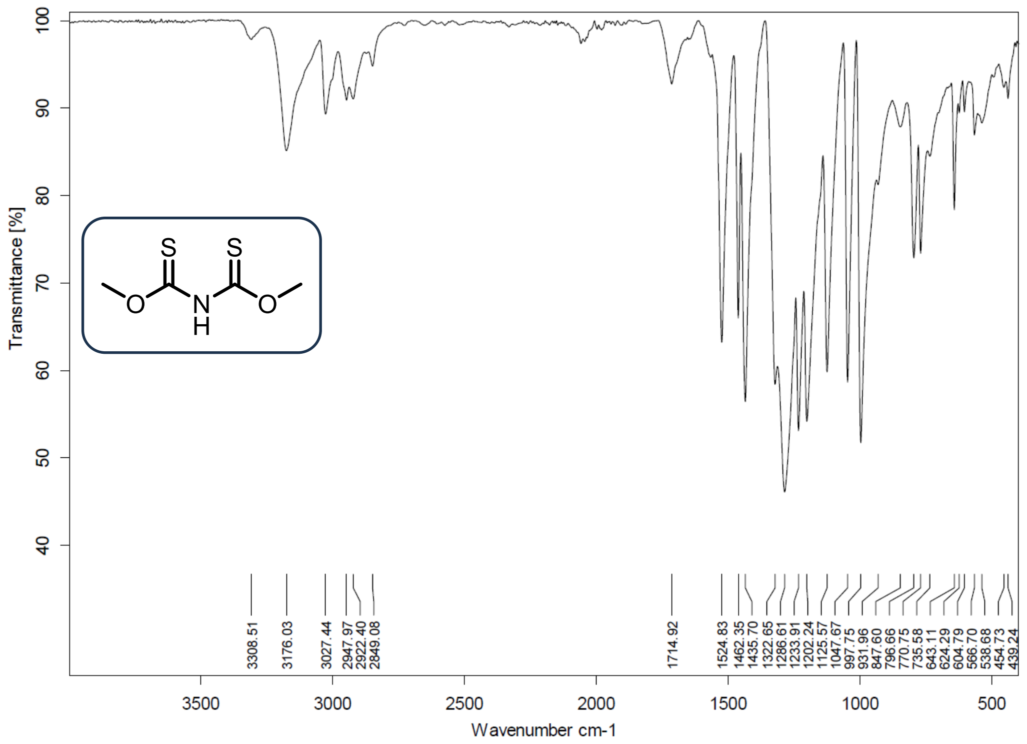

Figure S 3: IR spectrum of **1** measured at room temperature.

2.2 Imido-*C*,*C'*-dithiodicarbonic-*O*,*O'*-diisopropyl ester (3)

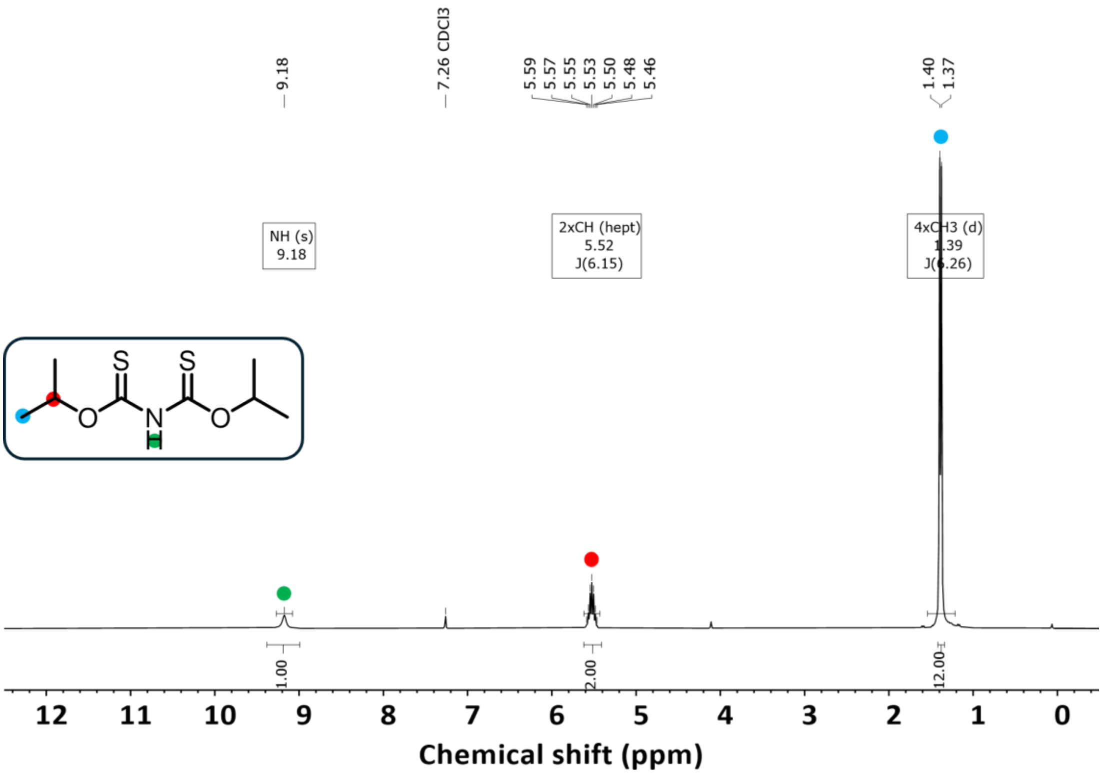

Figure S 4: ^{1}H-NMR spectrum of **3** in $CDCl_3$ at room temperature.

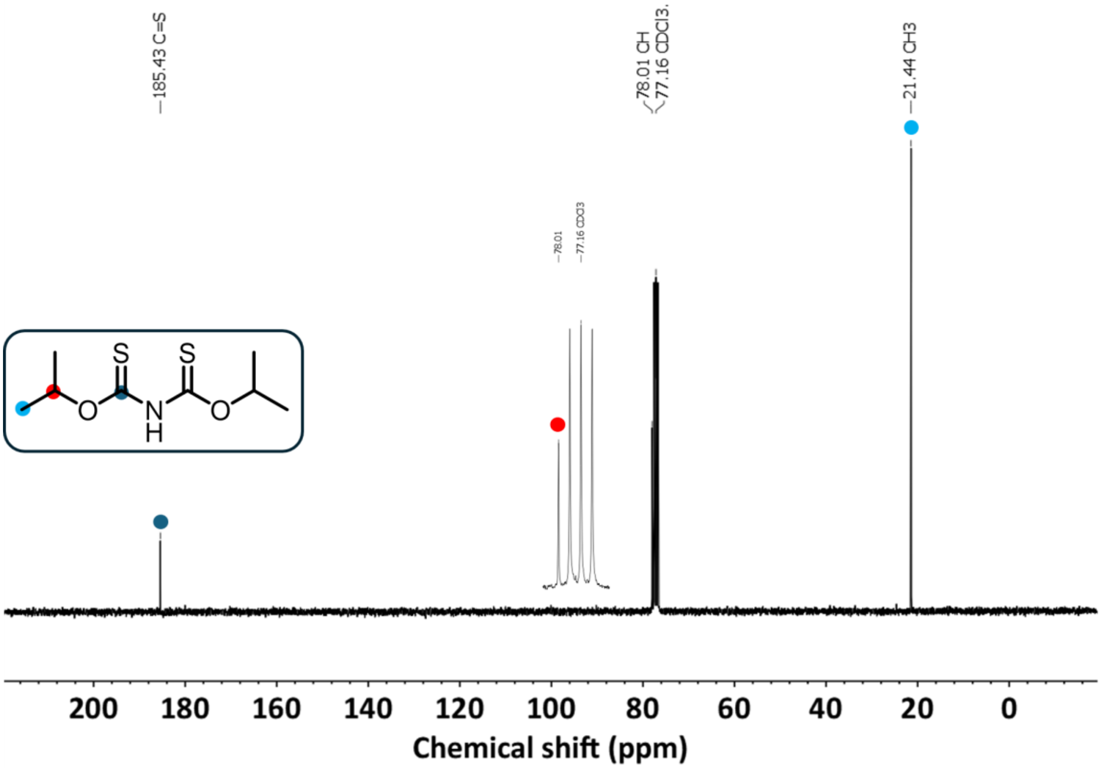

Figure S 5: ^{13}C-NMR spectrum of **3** in $CDCl_3$ at room temperature.

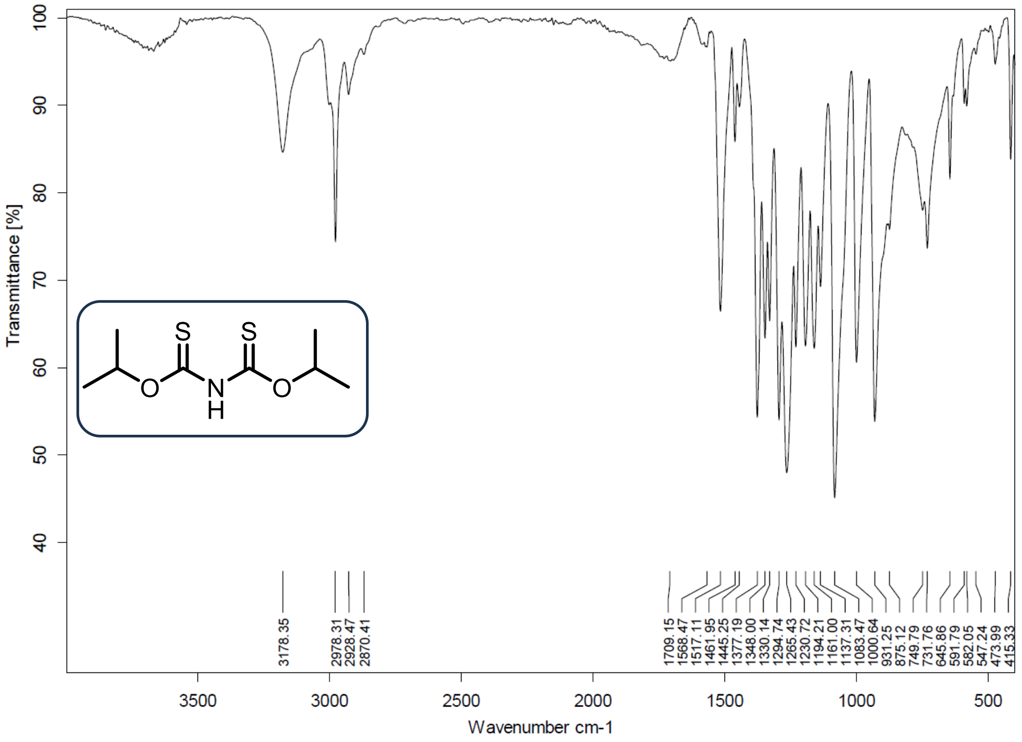

Figure S 6: IR spectrum of **3** measured at room temperature.

2.3 Sodium salt of imido-*C*,*C'*-dithiodicarbonic-*O*,*O'*-diisopropyl ester (Na[3])

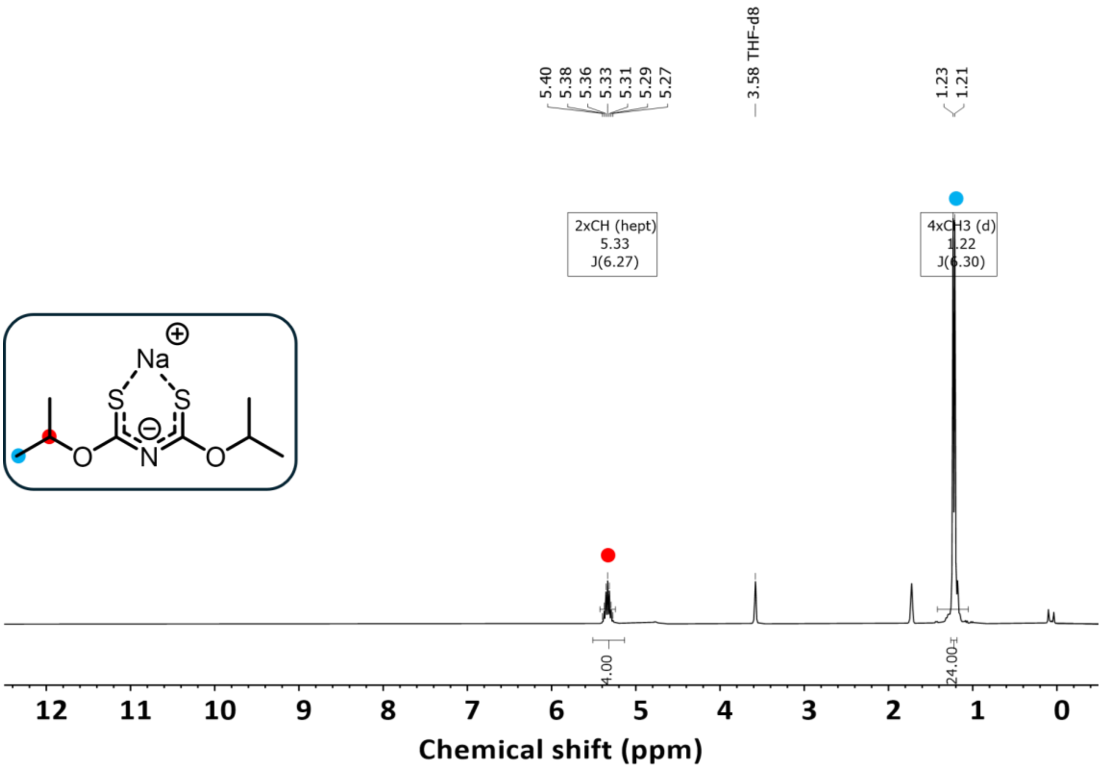

Figure S 7: ^{1}H-NMR spectrum of Na[**3**] in THF-d_8 at room temperature.

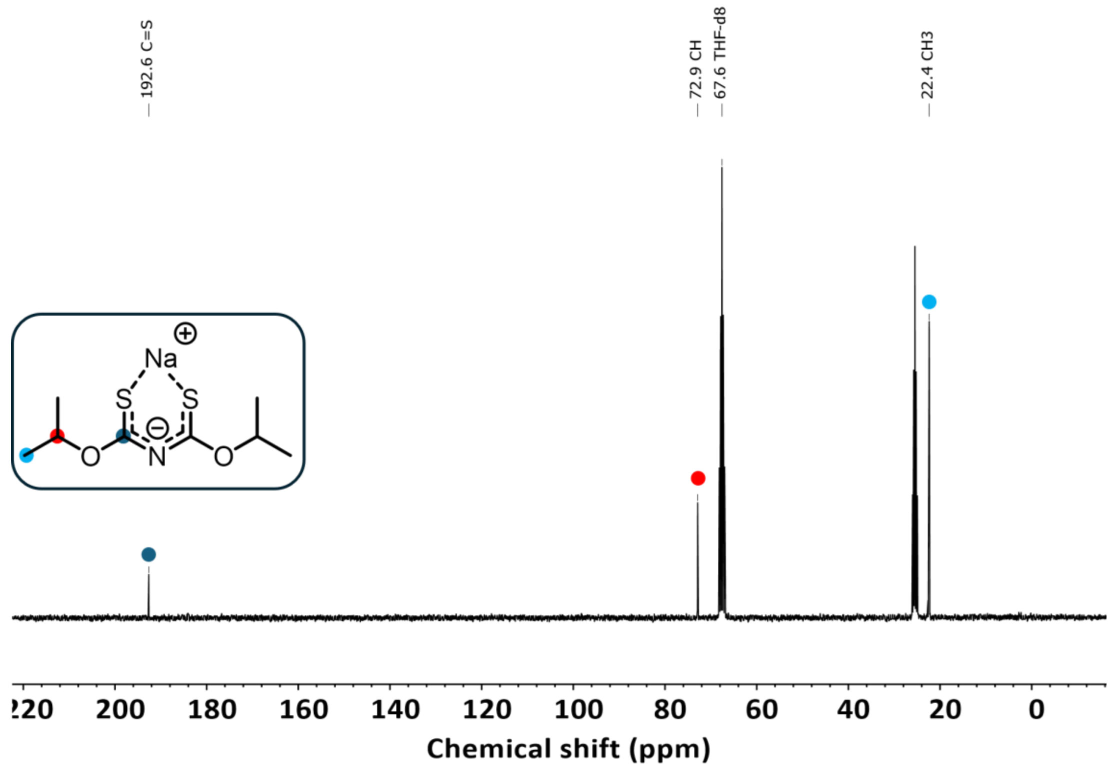

Figure S 8: ^{13}C-NMR spectrum of Na[**3**] in THF-d$_8$ at room temperature.

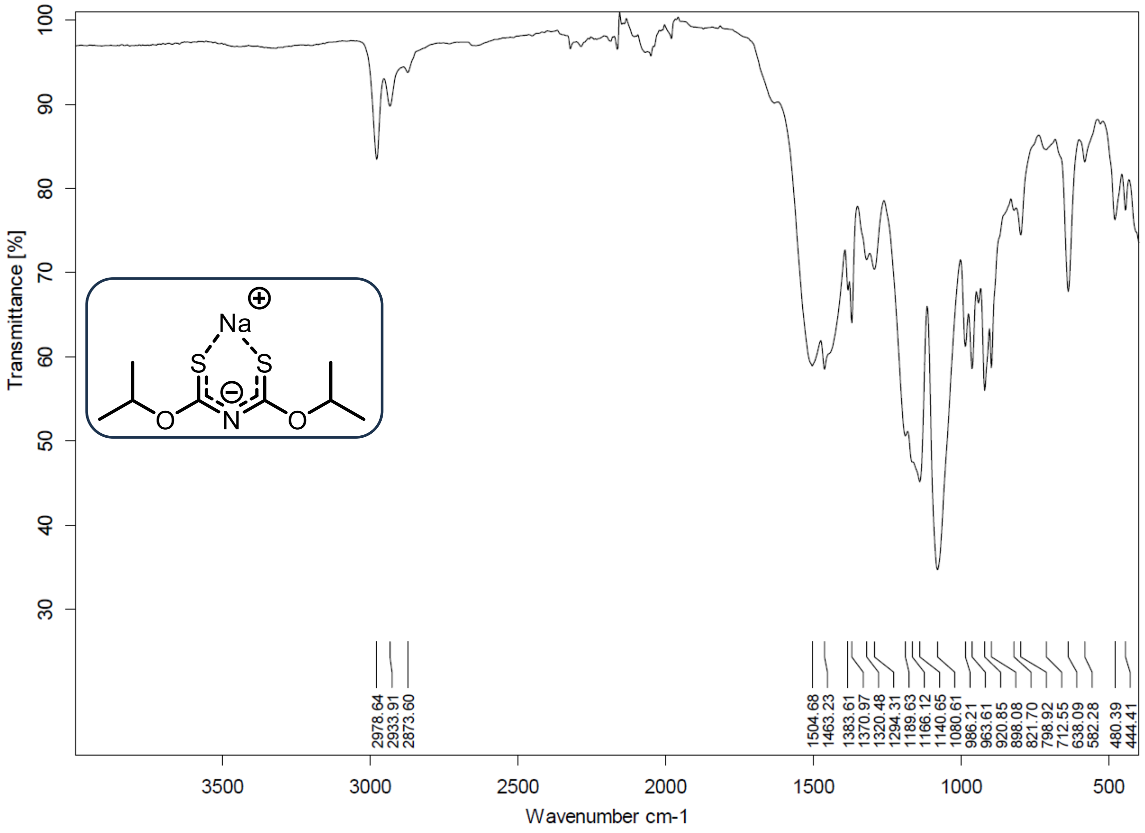

Figure S 9: IR spectrum of Na[**3**] measured at room temperature.

2.4 Potassium salt of imido-*C*,*C'*-dithiodicarbonic-*O*,*O'*-diisopropyl ester (K[3])

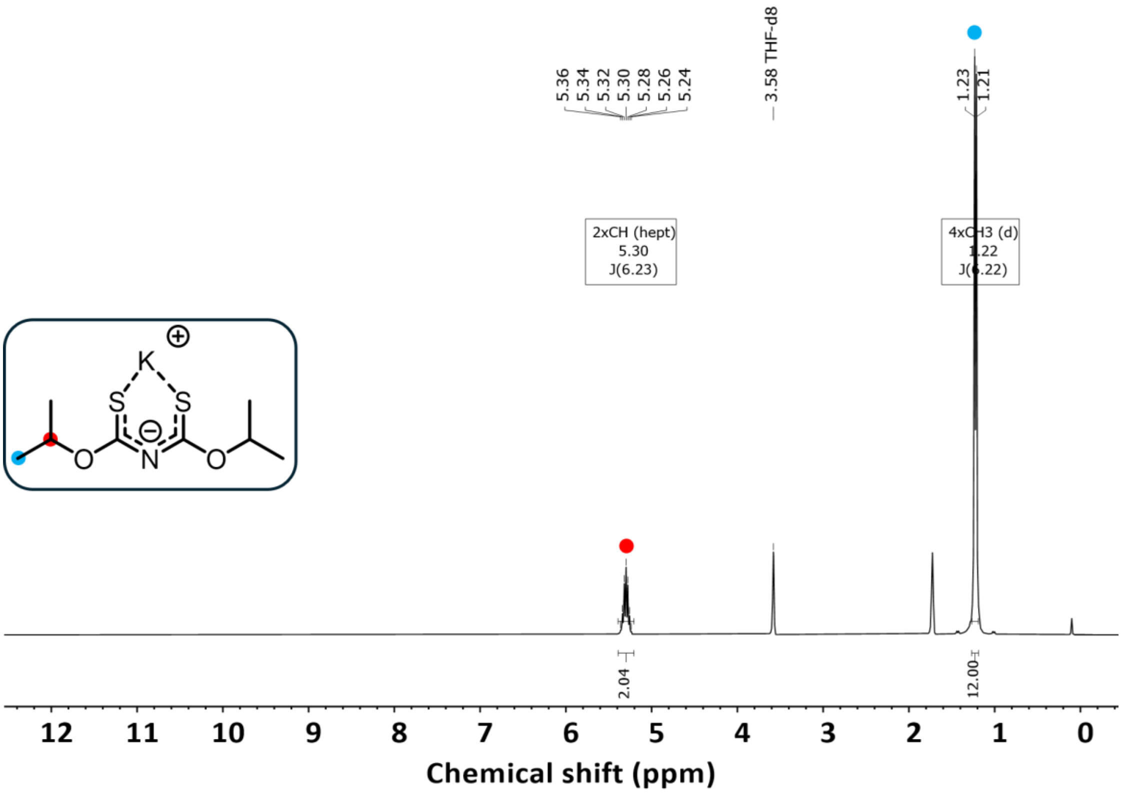

Figure S 10: ^{1}H-NMR spectrum of K[**3**] in THF-d_8 at room temperature.

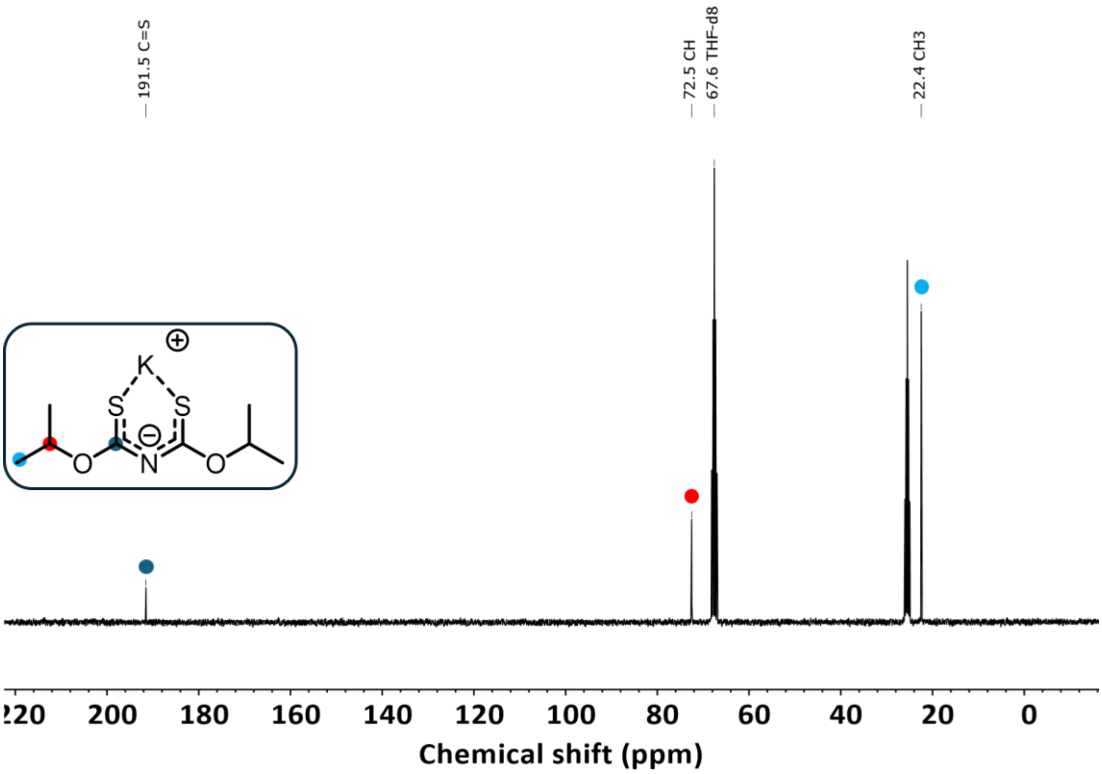

Figure S 11: ^{13}C-NMR spectrum of K[**3**] in THF-d_8 at room temperature.

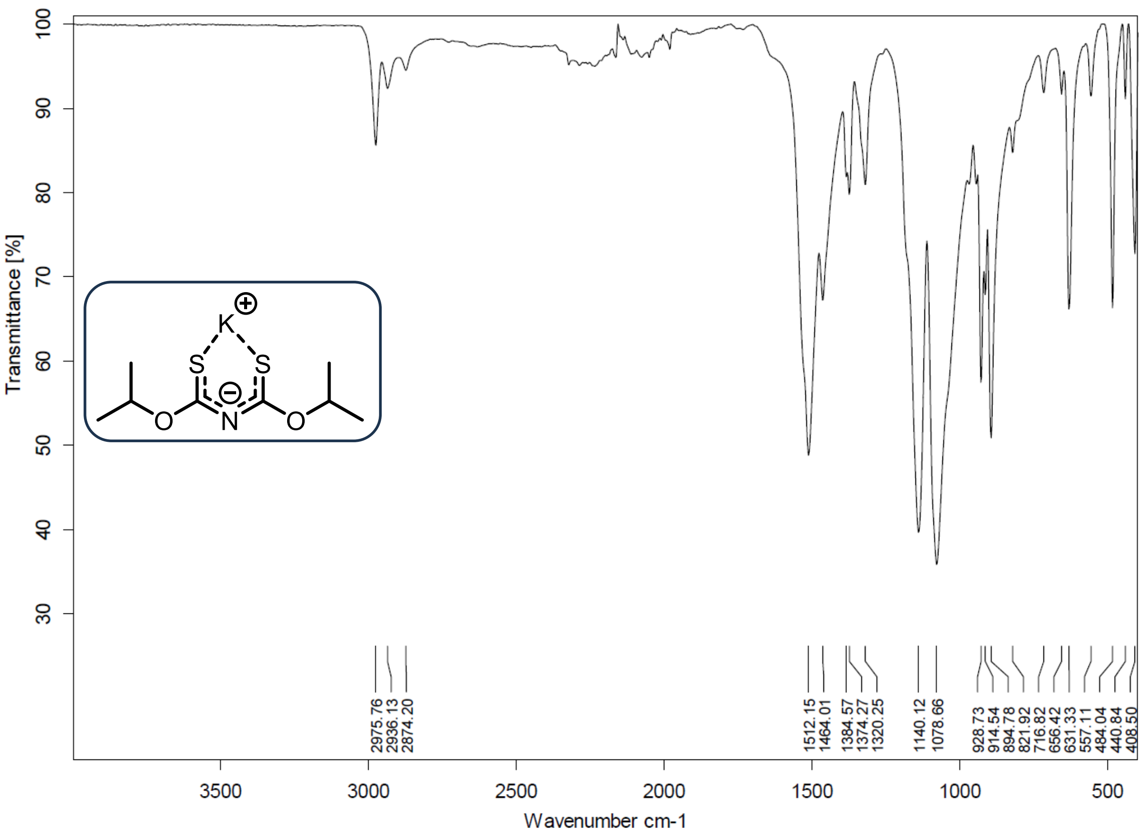

Figure S 12: IR spectrum of K[**3**] measured at room temperature.

2.5 Bis(imido-*C*,*C'*-dithiodicarbonic-*O*,*O'*-dimethyl ester)nickel(II) (Ni[1]$_2$)

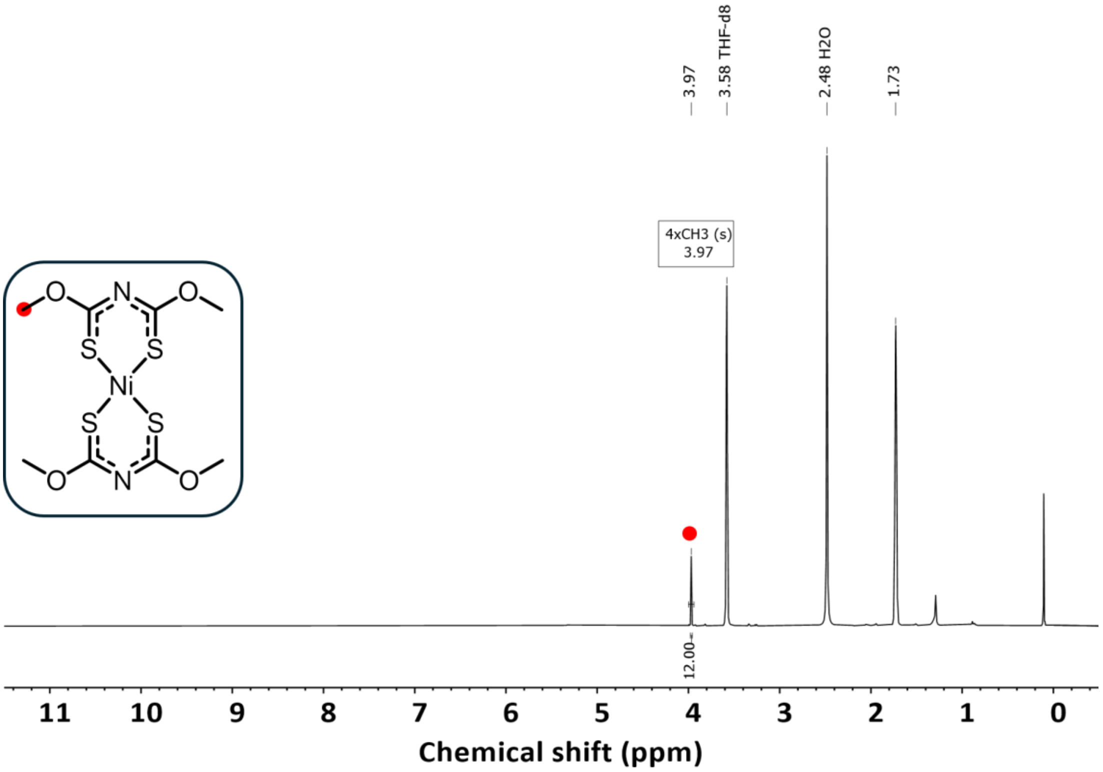

Figure S 13: ^{1}H-NMR spectrum of Ni[**1**]$_2$ in THF-d$_8$ at room temperature.

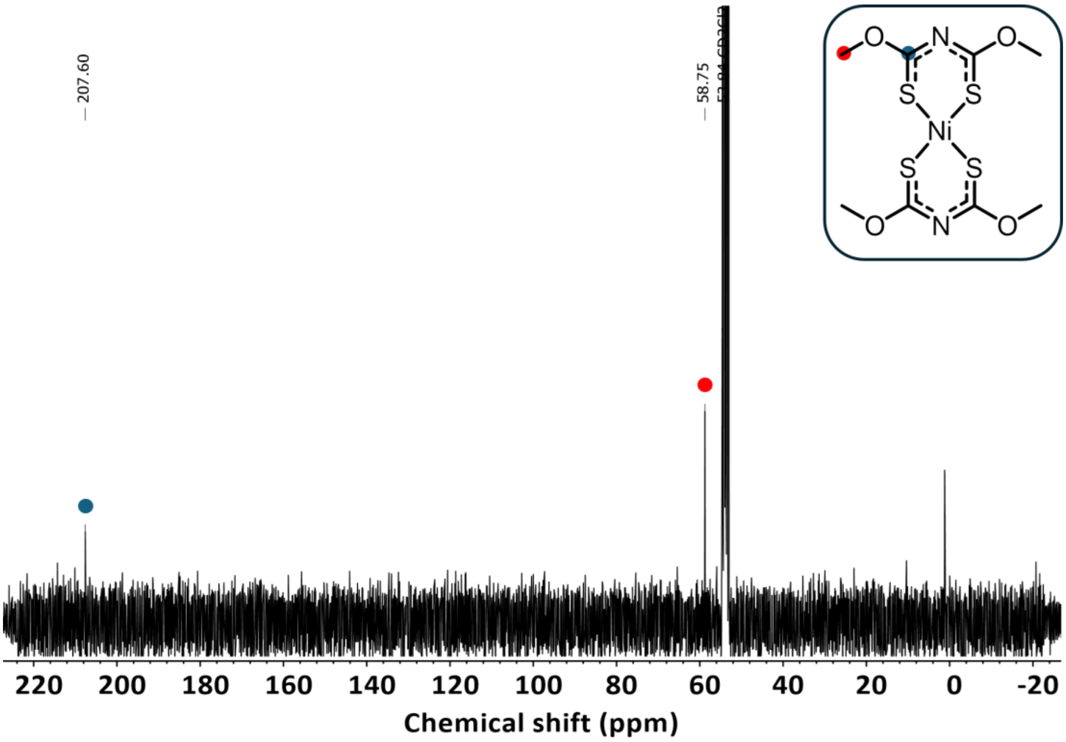

Figure S 14: ^{13}C-NMR spectrum of Ni[**1**]$_2$ in CD_2Cl_2 at room temperature.

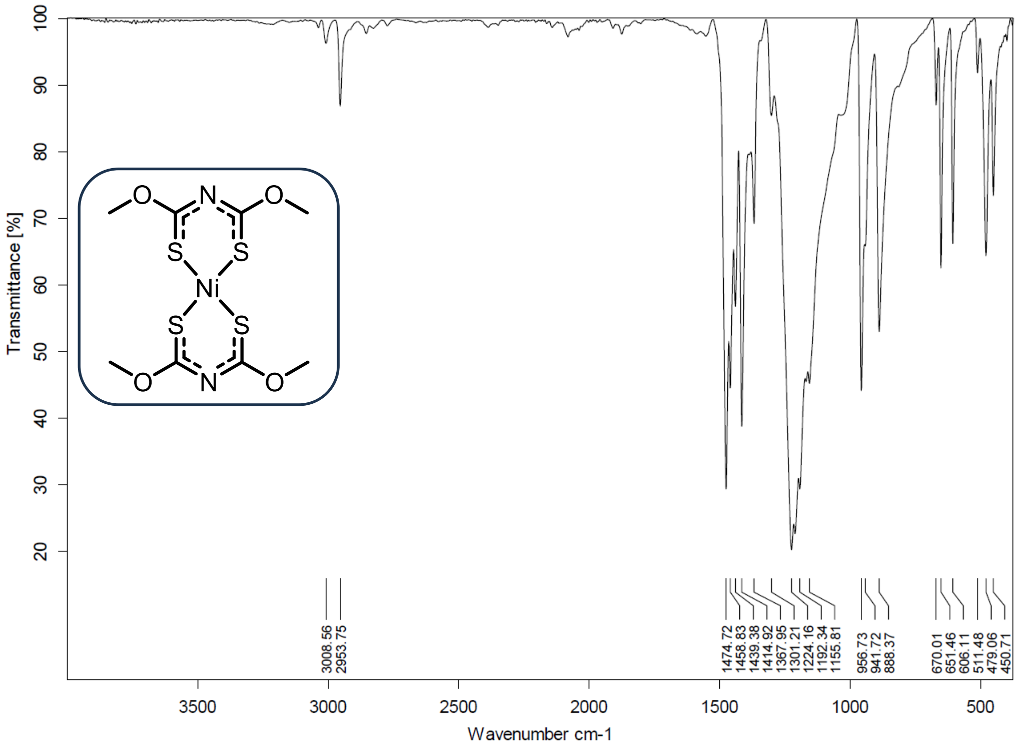

Figure S 15: IR spectrum of Ni[**1**]$_2$ measured at room temperature.

2.6 Bis(imido-*C*,*C'*-dithiodicarbonic-*O*,*O'*-dimethyl ester)palladium(II) (Pd[1]$_2$)

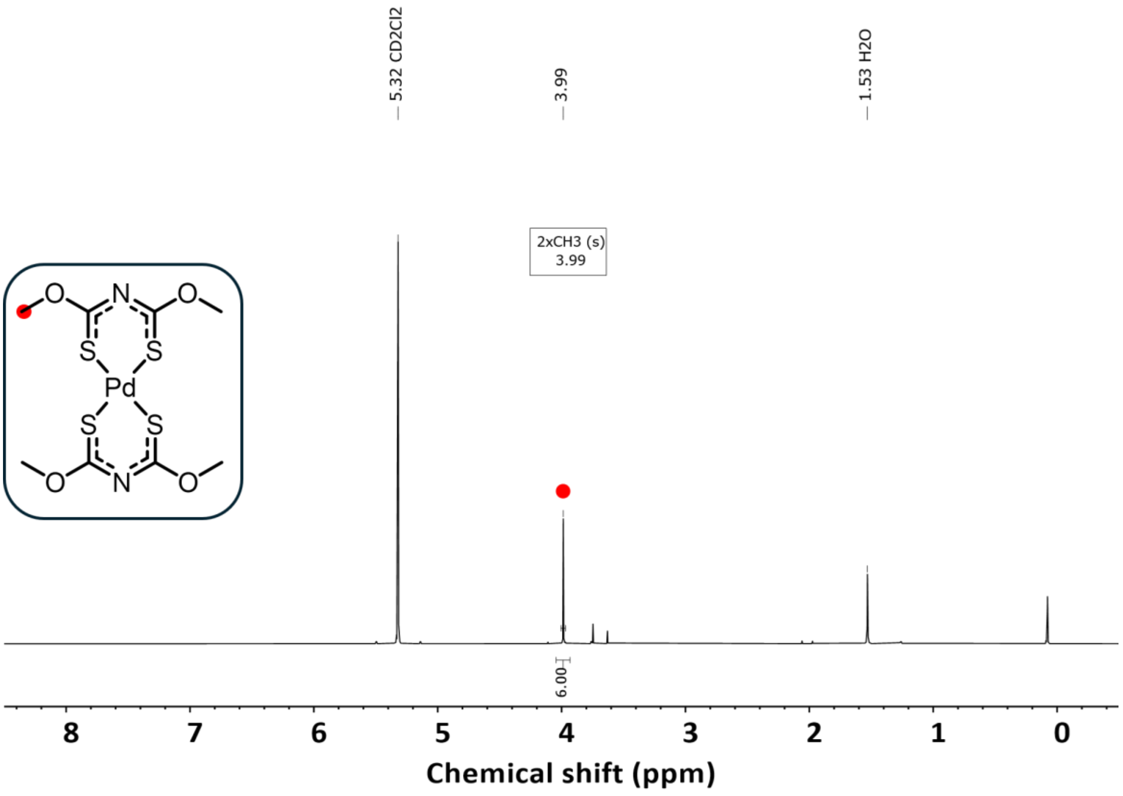

Figure S 16: ^{1}H NMR spectrum of Pd[**1**]$_2$ in CD_2Cl_2 at room temperature.

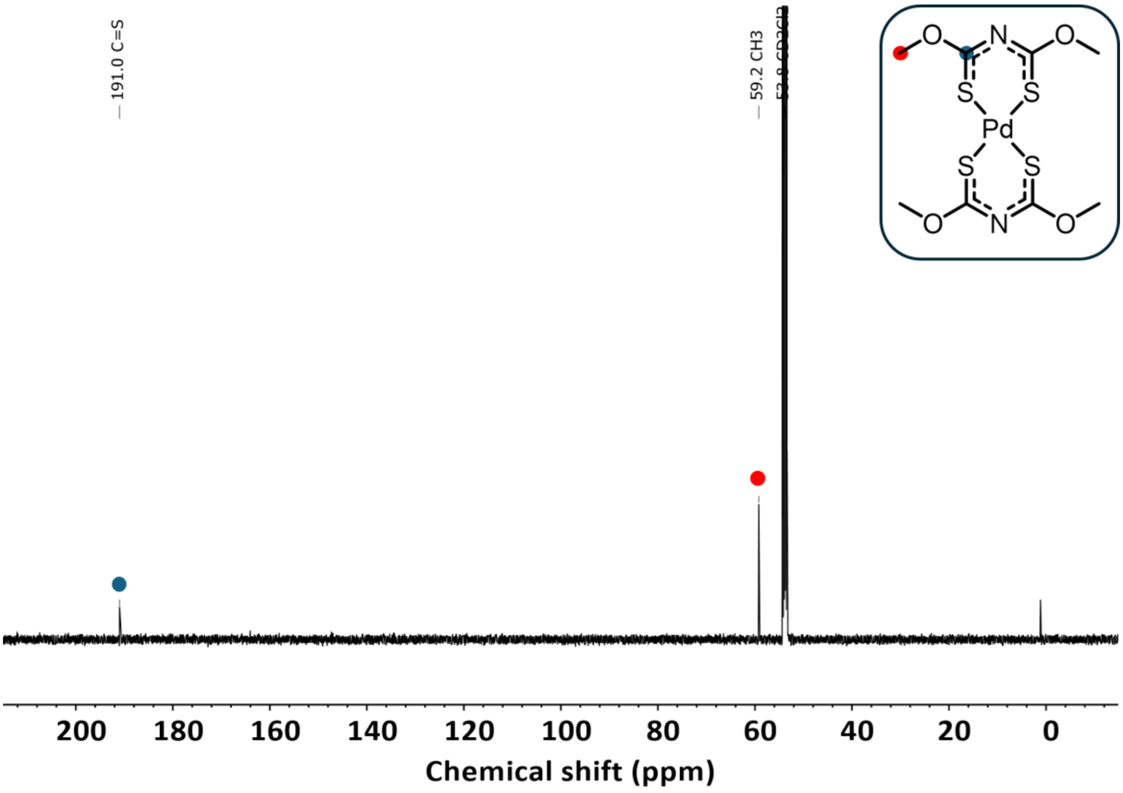

Figure S 17: ^{13}C-NMR spectrum of Pd[**1**]$_2$ in CD_2Cl_2 at room temperature.

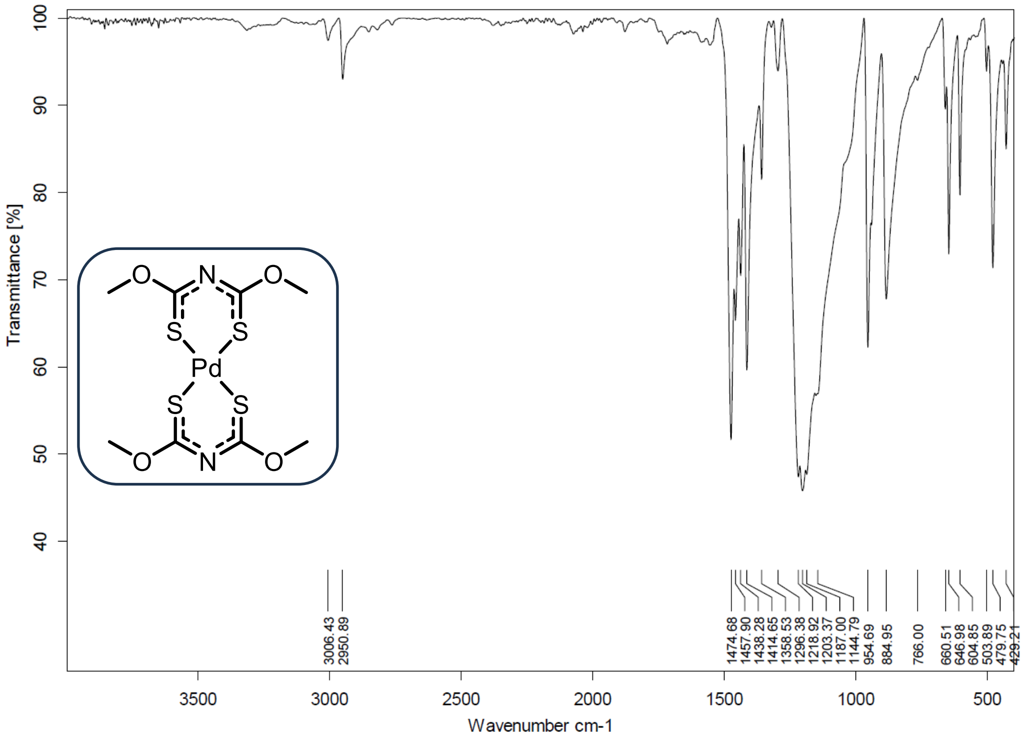

Figure S 18: IR spectrum of Pd[**1**]$_2$ measured at room temperature.

2.7 Bis(imido-*C*,*C'*-dithiodicarbonic-*O*,*O'*-dimethyl ester)platinum(II) (Pt[1]$_2$)

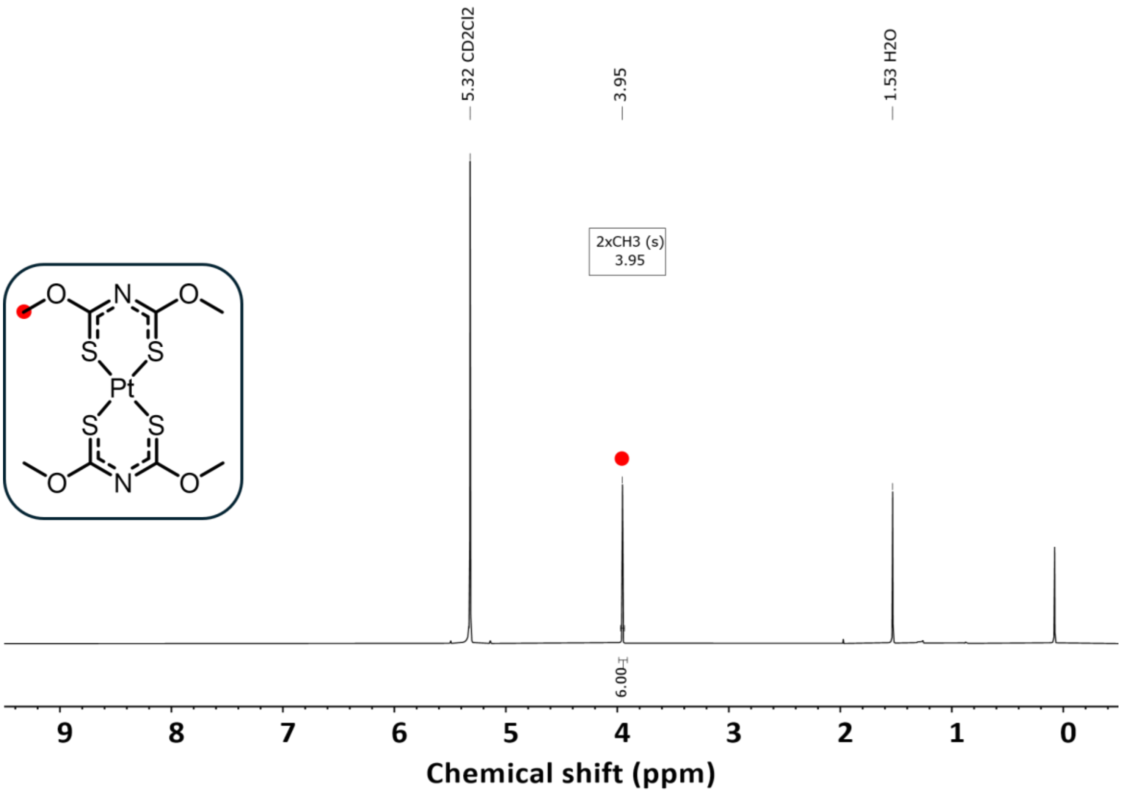

Figure S 19: ^{1}H-NMR spectrum of Pt[**1**]$_2$ in CD_2Cl_2 at room temperature.

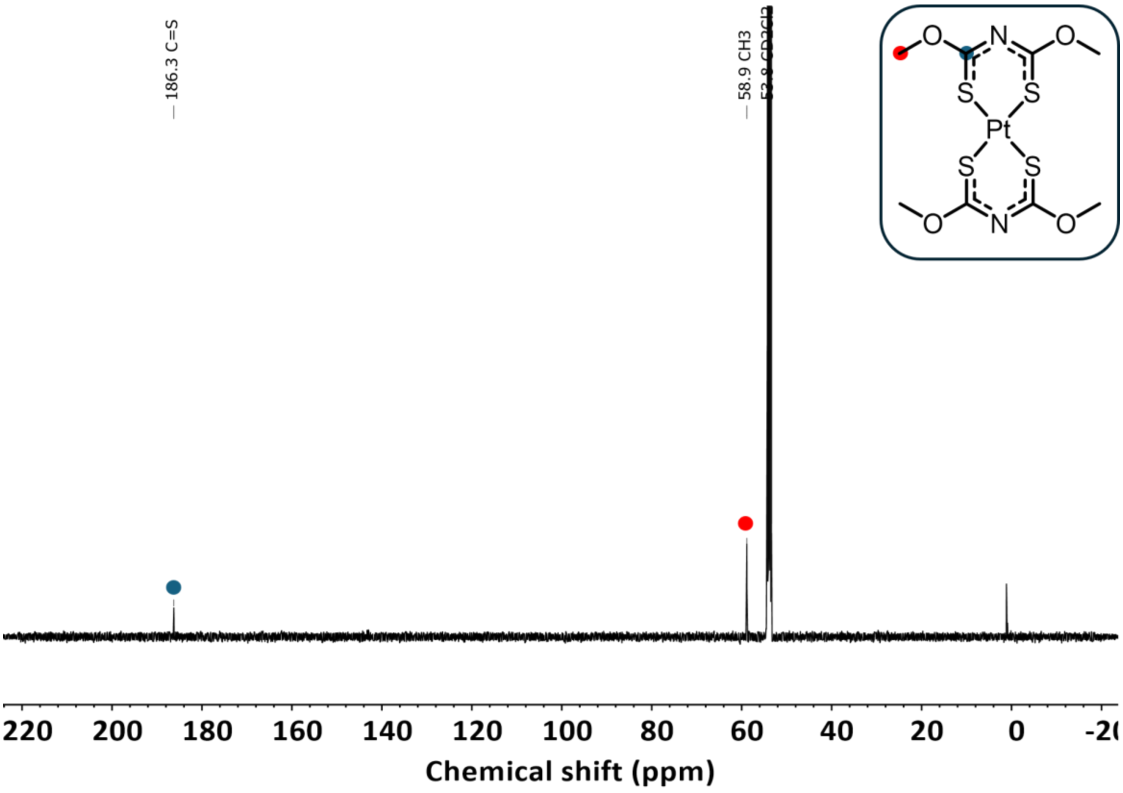

Figure S 20: ^{13}C-NMR spectrum of Pt[**1**]$_2$ in CD_2Cl_2 at room temperature.

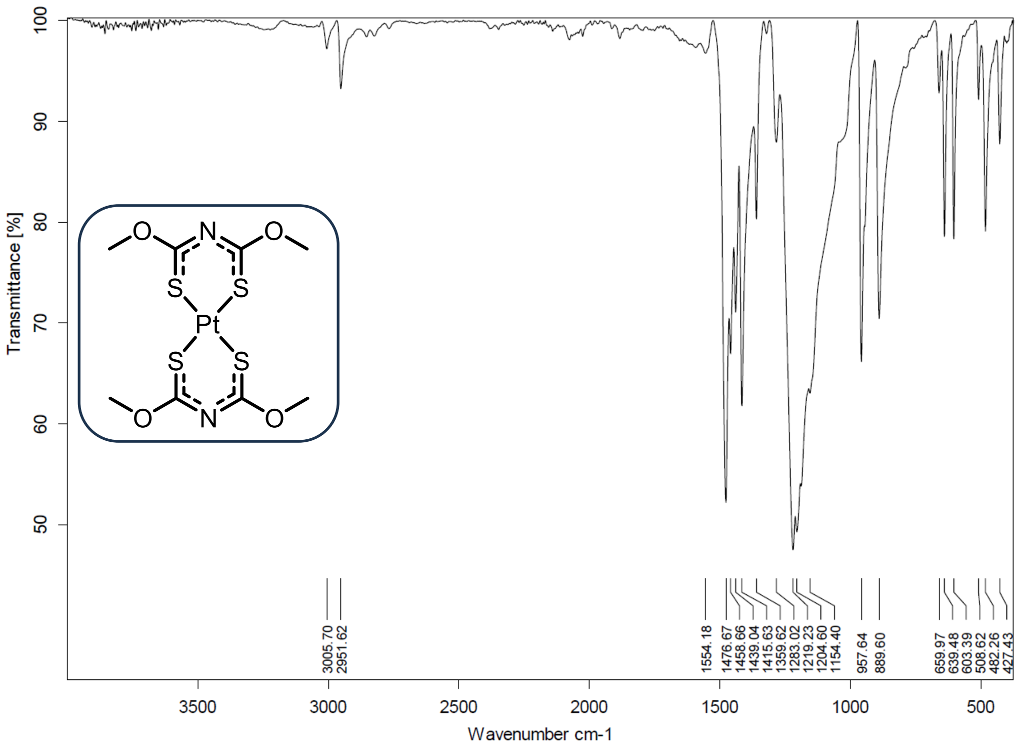

Figure S 21: IR spectrum of Pt[**1**]$_2$ measured at room temperature.

2.8 Bis(imido-*C*,*C'*-dithiodicarbonic-*O*,*O'*-diethyl ester)palladium(II) (Pd[**2**]$_2$)

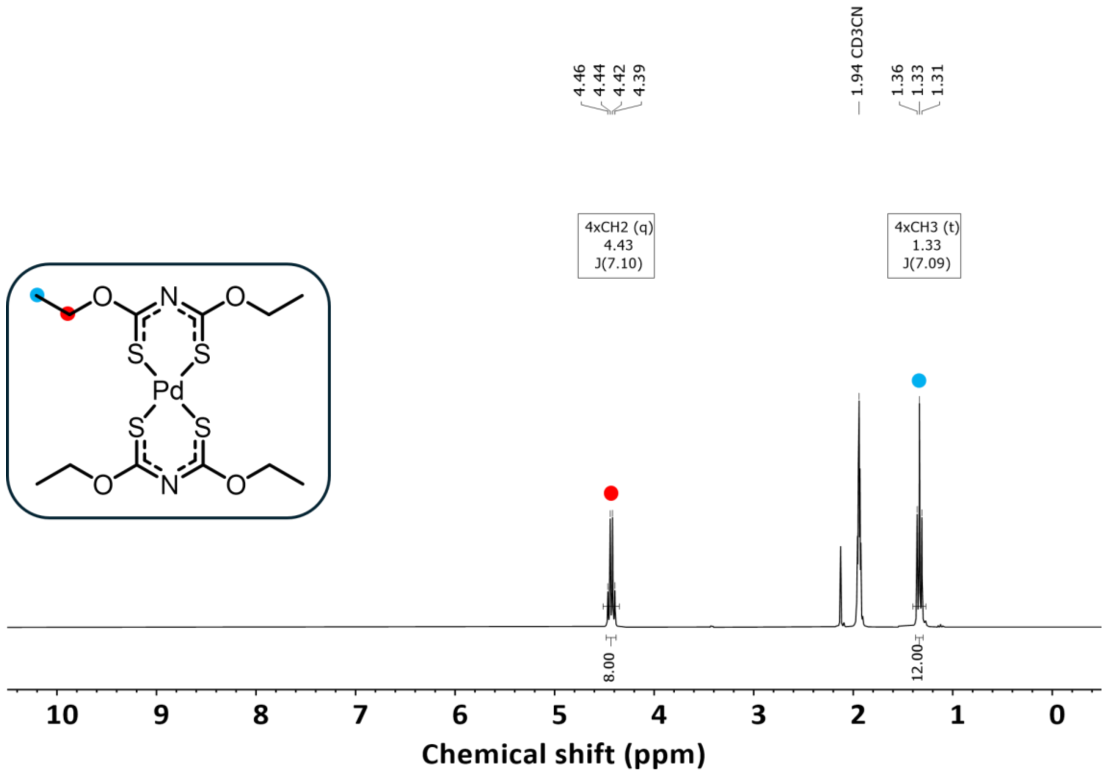

Figure S 22: ^{1}H-NMR spectrum of Pd[**2**]$_2$ in CD_3CN at room temperature.

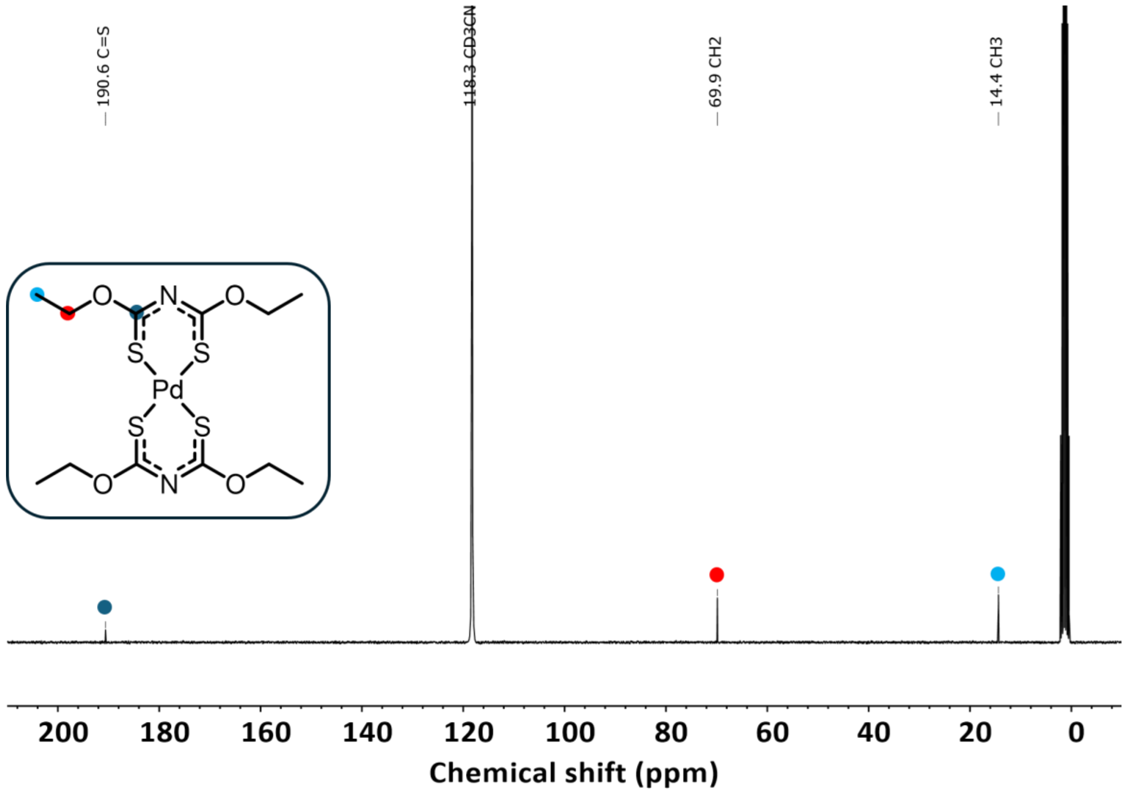

Figure S 23: ^{13}C-NMR spectrum of Pd[**2**]$_2$ in CD_3CN at room temperature.

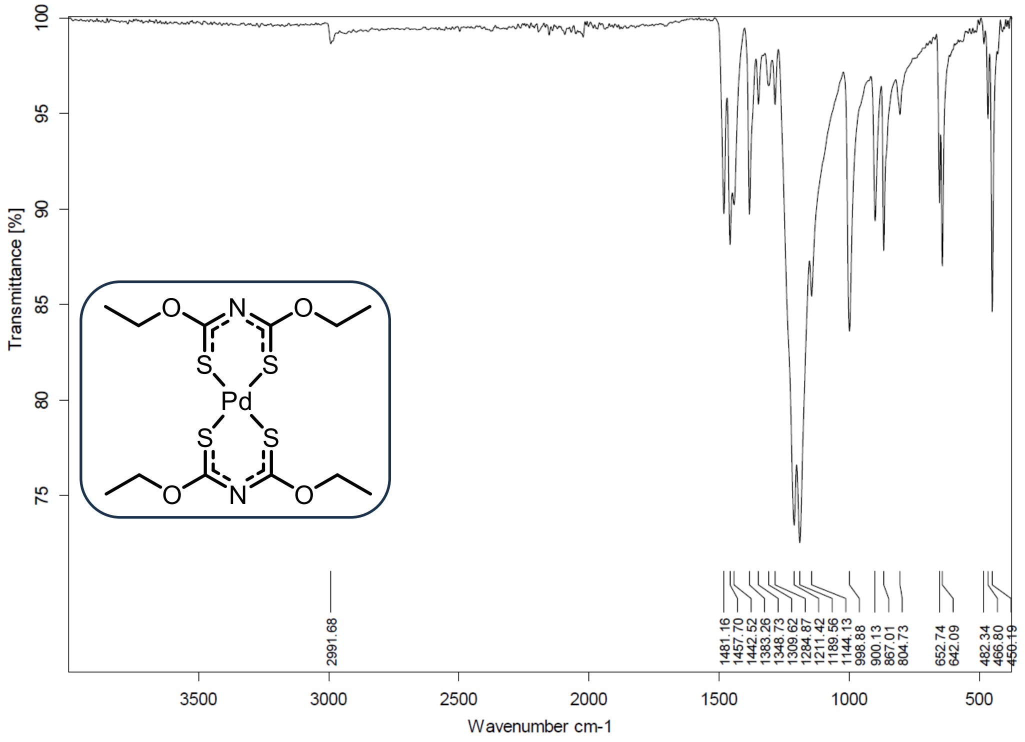

Figure S 24: IR spectrum of Pd[**2**]$_2$ measured at room temperature.

2.9 Bis(imido-*C*,*C'*-dithiodicarbonic-*O*,*O'*-diethyl ester)platinum(II) (Pt[2]$_2$)

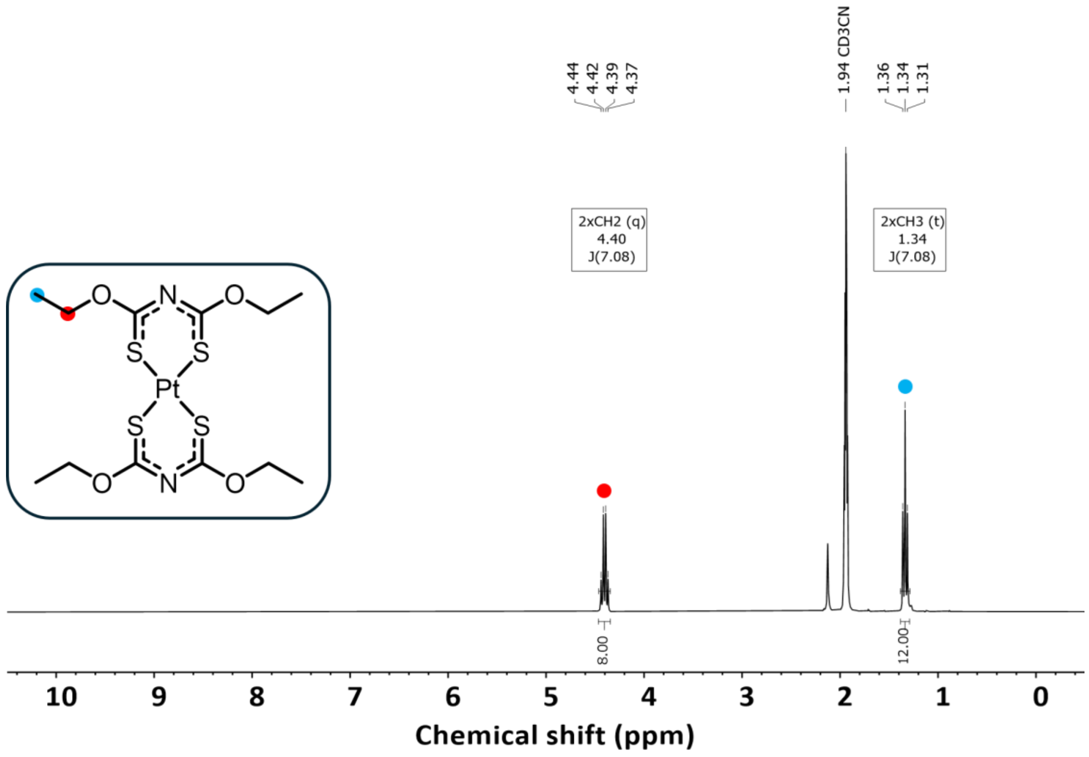

Figure S 25: ^{1}H-NMR spectrum of Pt[**2**]$_2$ in CD_3CN at room temperature.

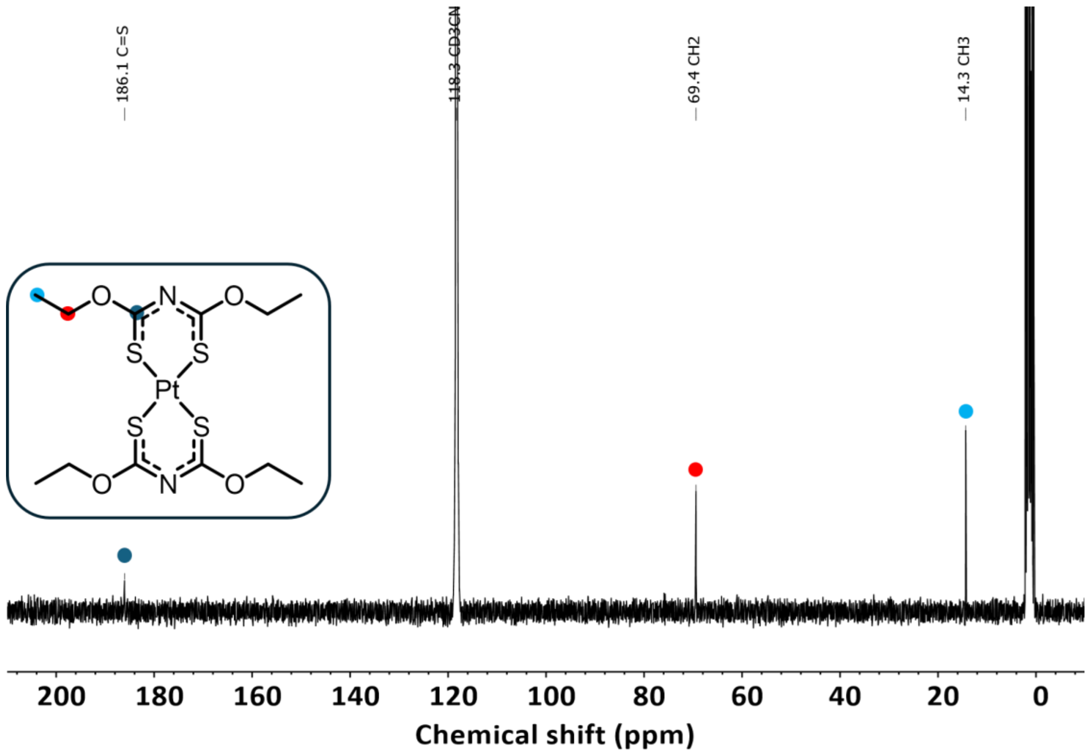

Figure S 26: ^{13}C-NMR spectrum of Pt[**2**]$_2$ in CD_3CN at room temperature.

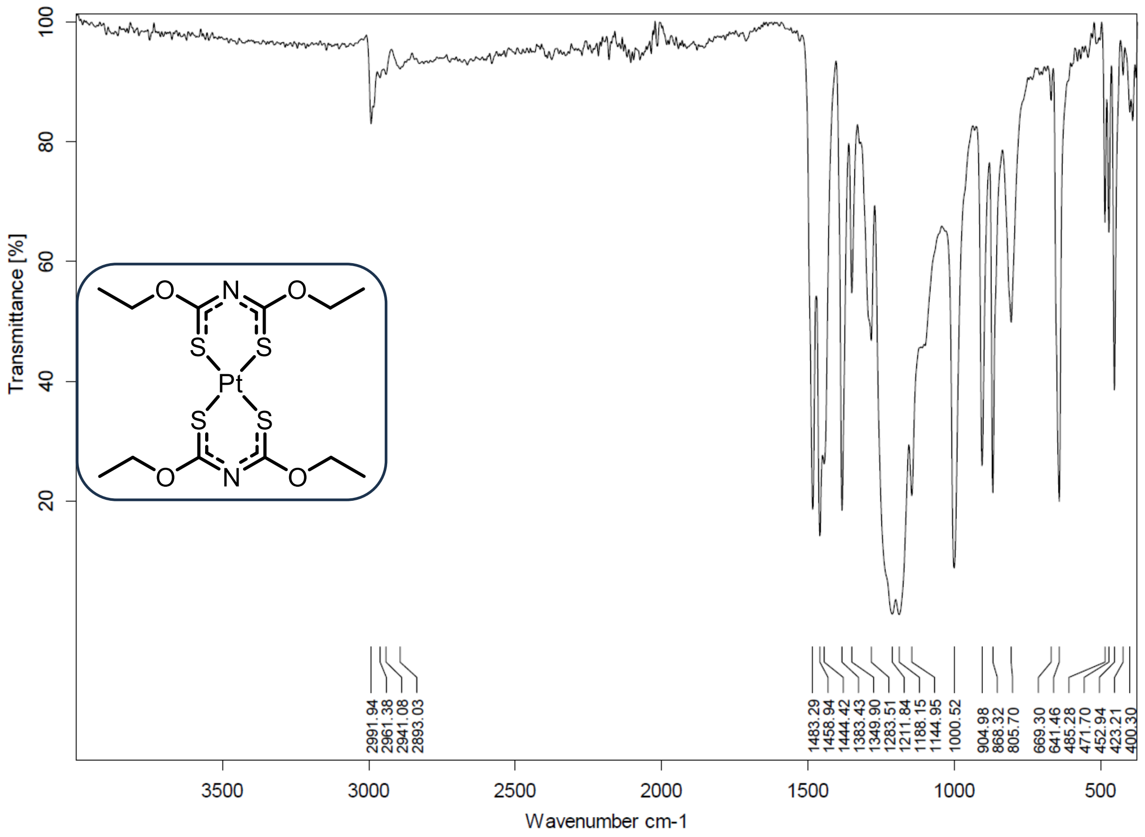

Figure S 27: IR spectrum of Pt[**2**]$_2$ measured at room temperature.

2.10 Bis(imido-*C,C'*-dithiodicarbonic-*O,O'*-diisopropyl ester)nickel(II) (Ni[3]$_2$)

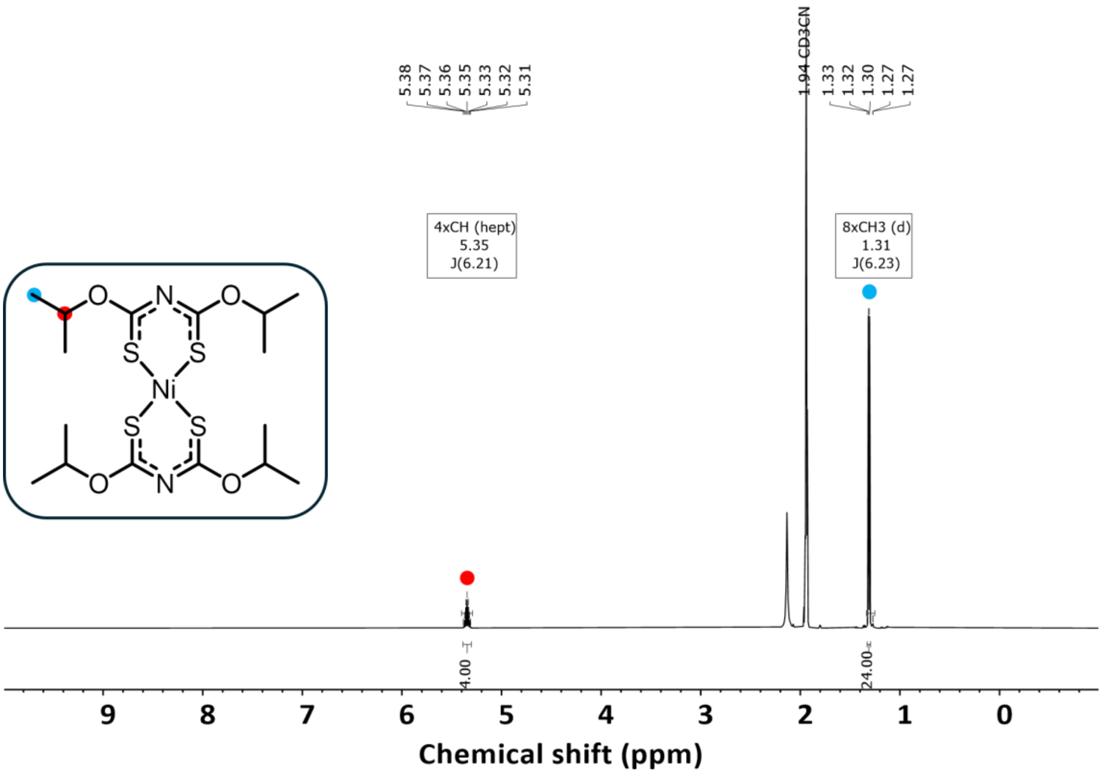

Figure S 28: ^{1}H-NMR spectrum of Ni[**3**]$_2$ in CD_3CN at room temperature.

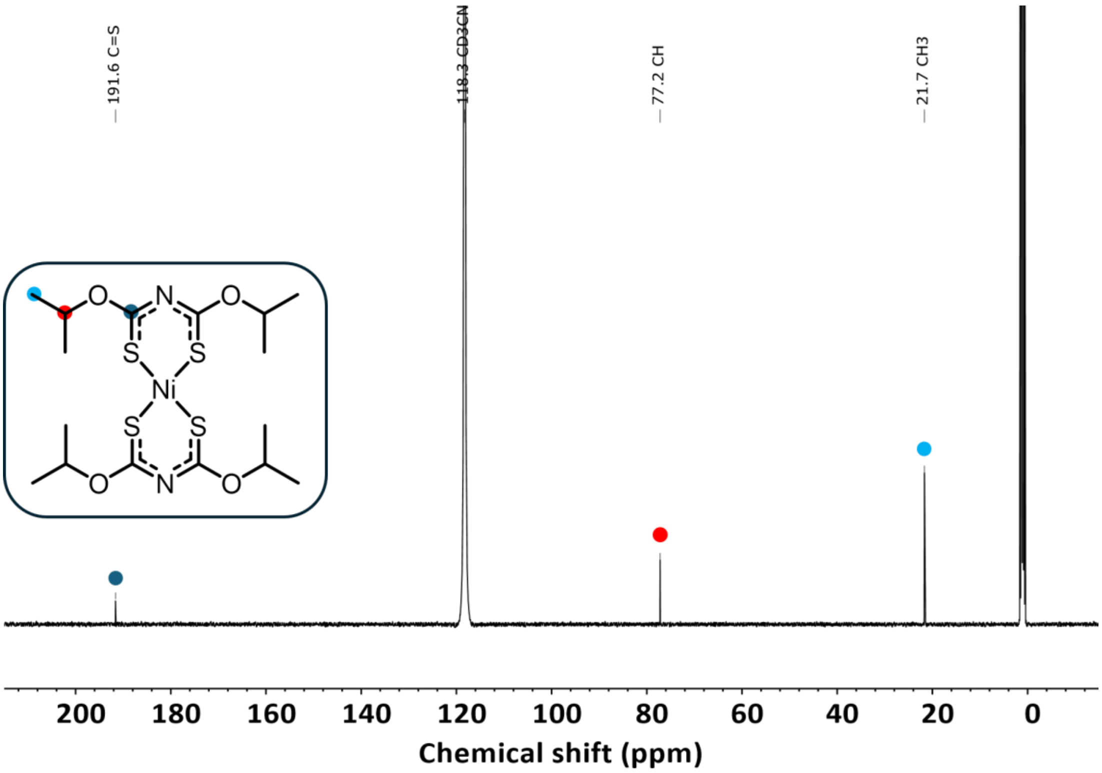

Figure S 29: ^{13}C-NMR spectrum of Ni[**3**]$_2$ in CD_3CN at room temperature.

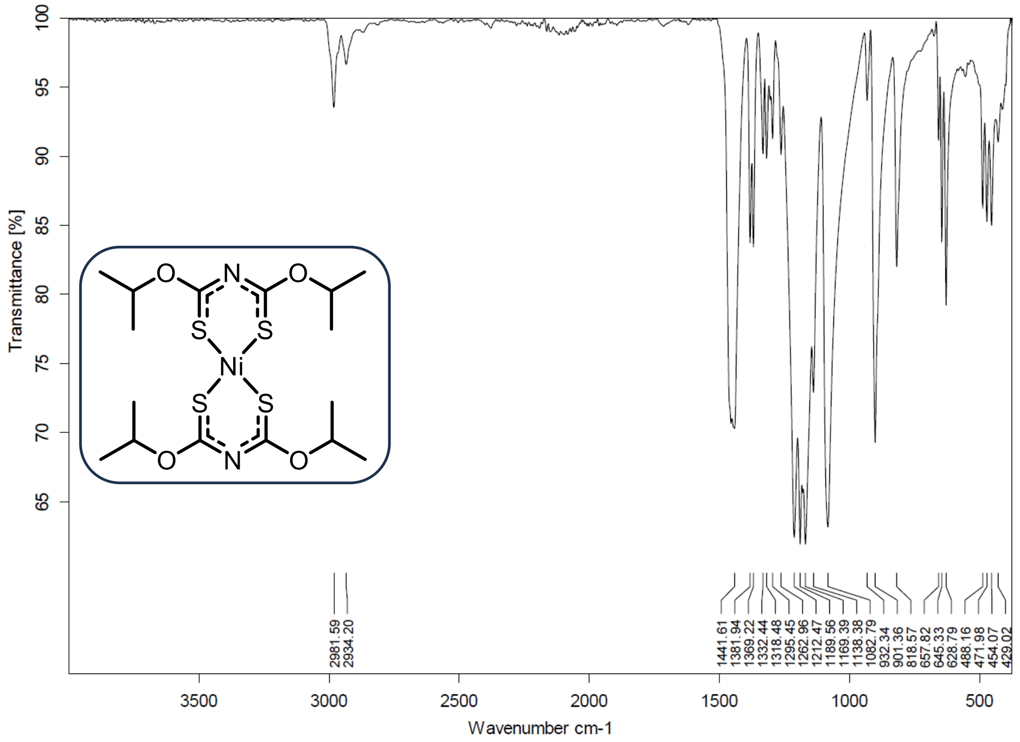

Figure S 30: IR spectrum of Ni[**3**]$_2$ measured at room temperature.

2.11 Bis(imido-*C,C'*-dithiodicarbonic-*O,O'*-diisopropyl ester)palladium(II) (Pd[3]$_2$)

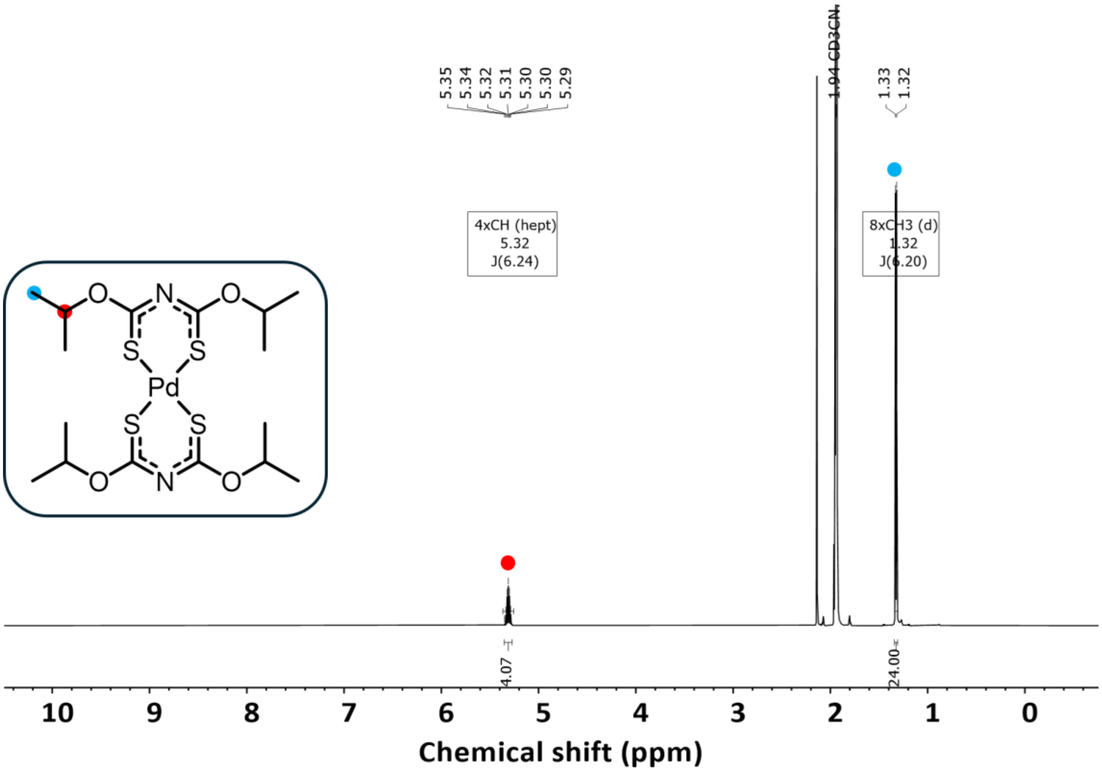

Figure S 31: ^{1}H-NMR spectrum of Pd[**3**]$_2$ in CD_3CN at room temperature.

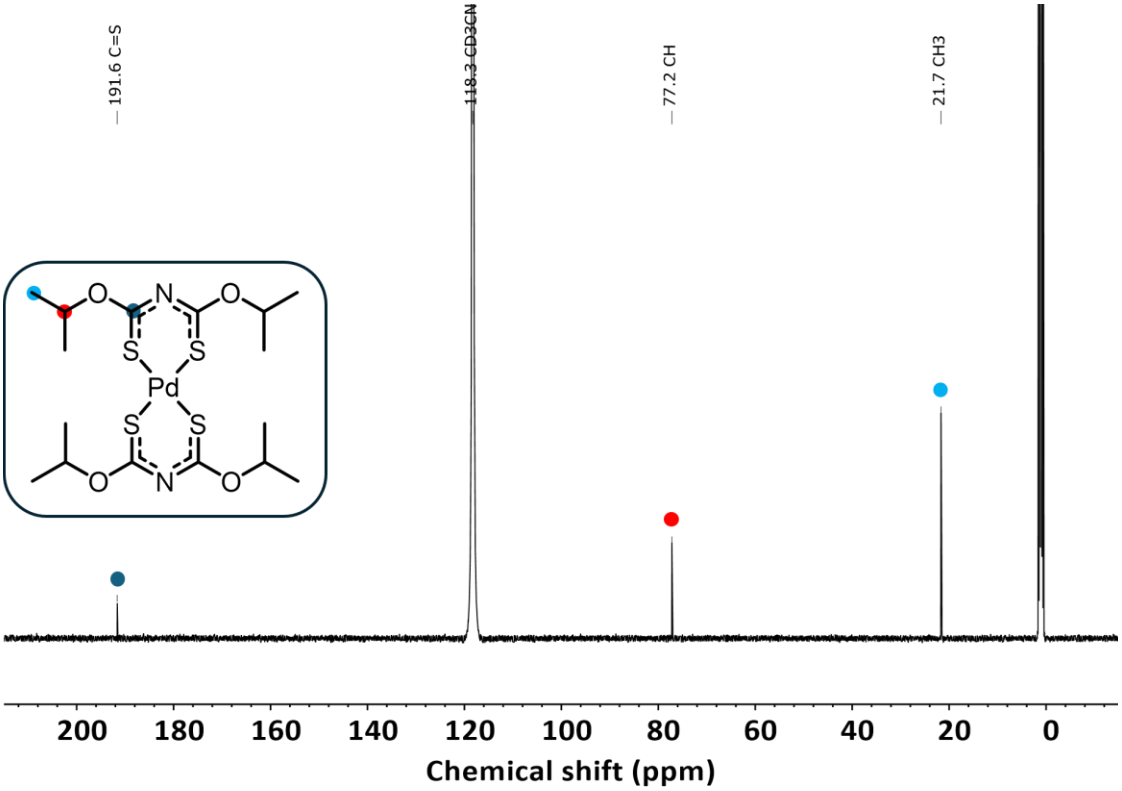

Figure S 32: ^{13}C-NMR spectrum of Pd[**3**]$_2$ in CD_3CN at room temperature.

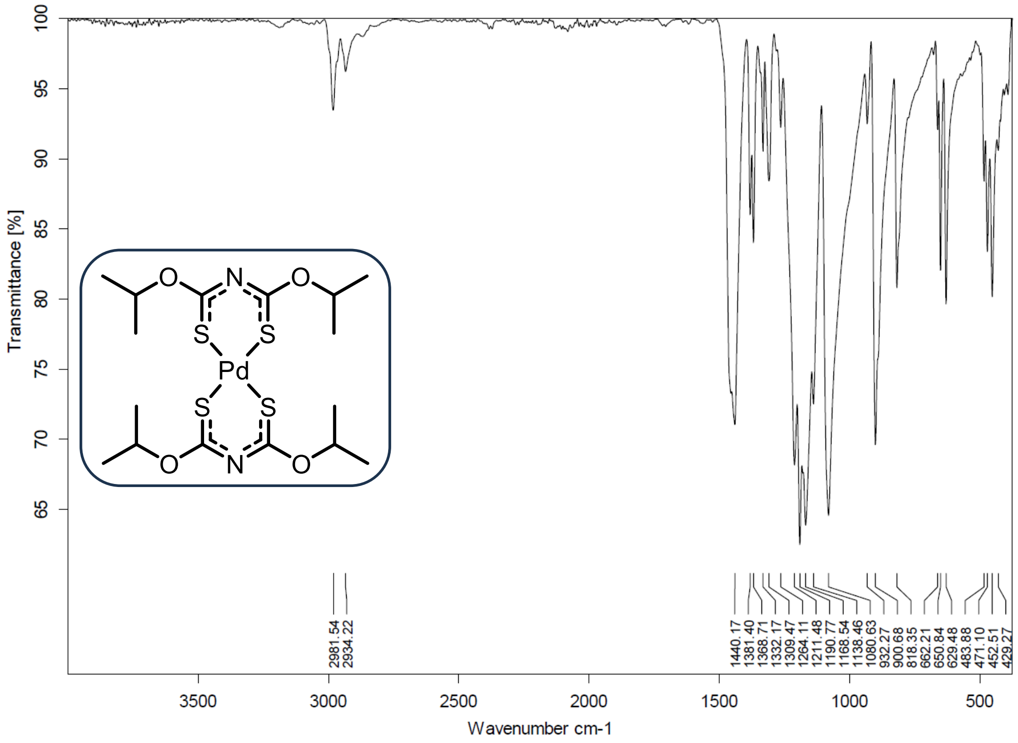

Figure S 33: IR spectrum of Pd[**3**]$_2$ measured at room temperature.

2.12 Bis(imido-*C,C'*-dithiodicarbonic-*O,O'*-diisopropyl ester)platinum(II) (Pt[3]$_2$)

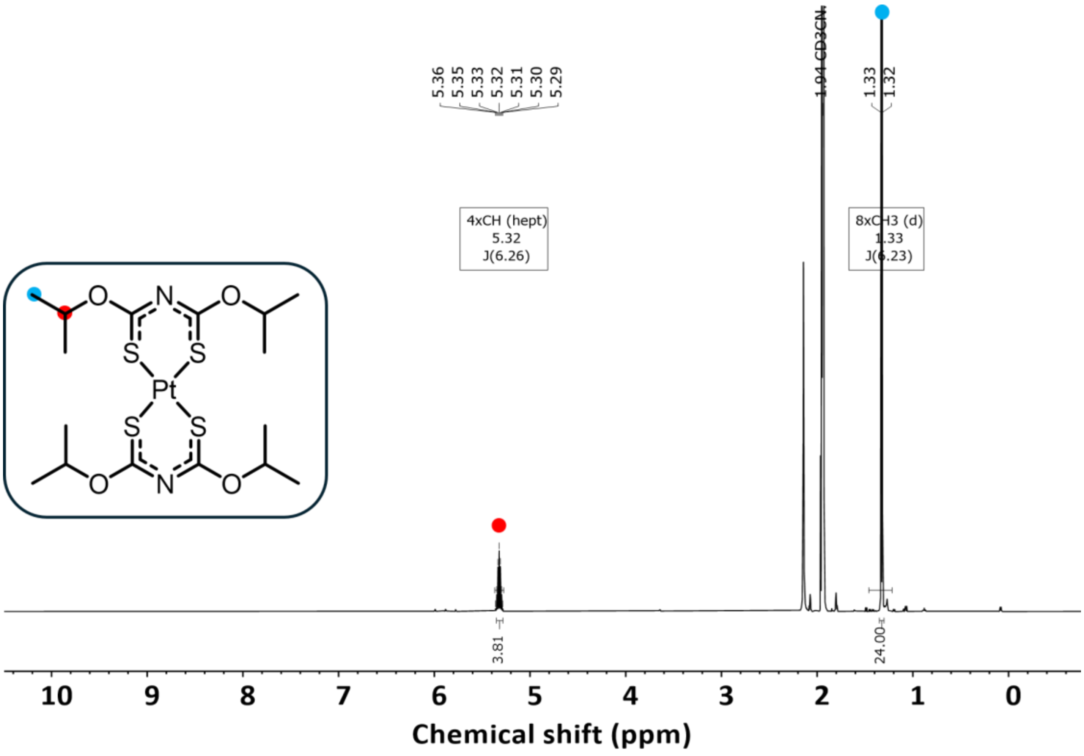

Figure S 34: ^{1}H-NMR spectrum of Pt[**3**]$_2$ in CD_3CN at room temperature.

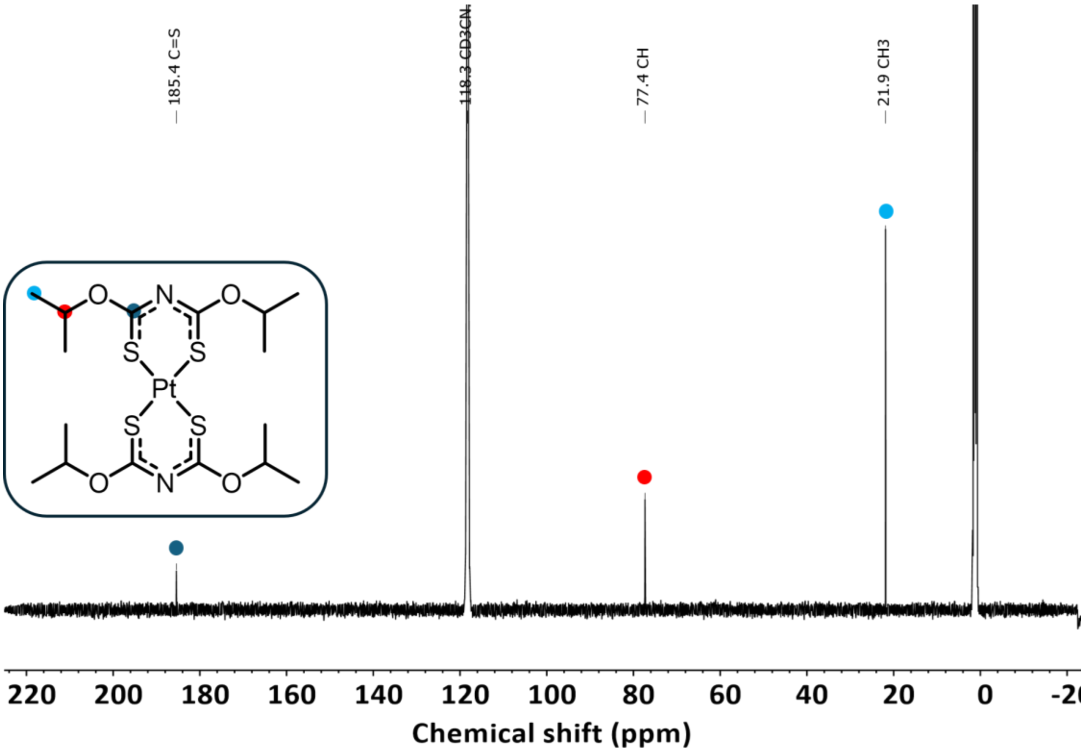

Figure S 35: ^{13}C-NMR spectrum of Pt[**3**]$_2$ in CD_3CN at room temperature.

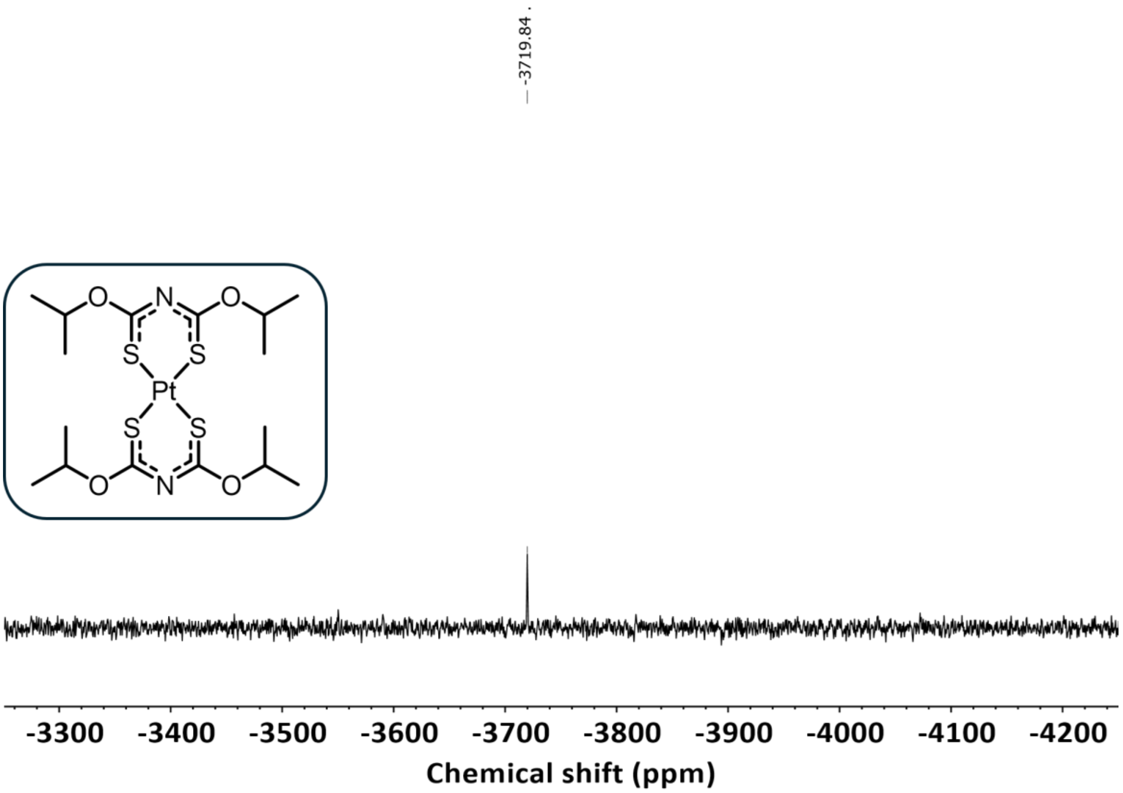

Figure S 36: ^{195}Pt-NMR spectrum of Pt[**3**]$_2$ in CD_3CN at room temperature.

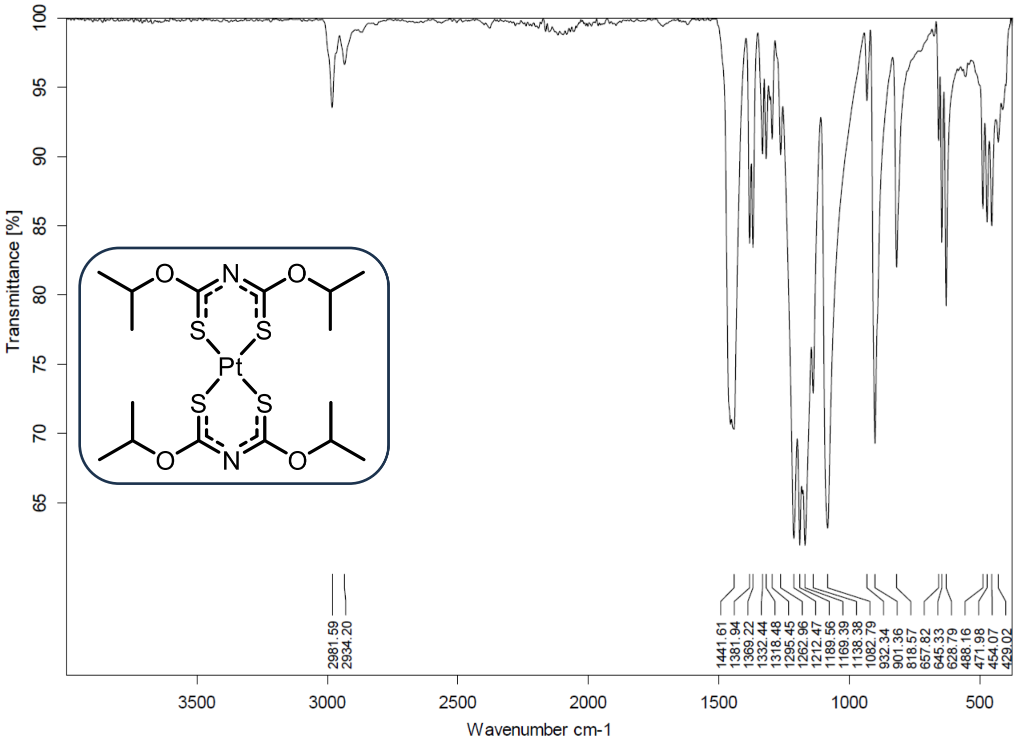

Figure S 37: IR spectrum of Pt[**3**]$_2$ measured at room temperature.

2.13 Unsuccessful reactions of thiocarbonyl dithiocyanate with selected alcohols

^{tert}Butanol

Thiocarbonyl dithiocyanate (0.80 g, 4.99 mmol, 1.00 eq.) was dissolved in dichloromethane (5 mL) and cooled to −80 °C (cooling leads to precipitation of the thiocarbonyl dithiocyanate). ^{tert}Butanol (5.00 mL, 53.20 mmol, 14.47 eq.) was added dropwise into the orange suspension. After complete addition, the cooling bath was removed, and the reaction mixture was allowed to warm up to room temperature. After 18 h of stirring at room temperature, all volatiles were removed *in vacuo* yielding a dark brown-red amorphous residue.

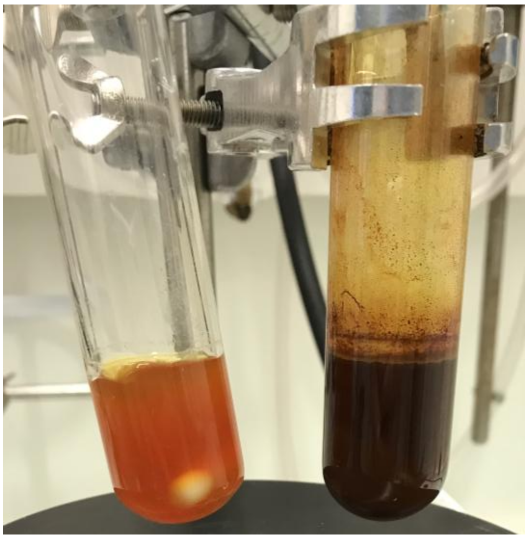

Figure S 38: Comparison of reaction mixtures of thiocarbonyl dithiocyanate with ^{iso}propanol (left) and ^{tert}butanol (right).

Benzyl alcohol

Thiocarbonyl dithiocyanate (2.1 g, 13.11 mmol, 1.00 eq.) was suspended in toluene (10 mL) and cooled to −40 °C. Then the cooling bath was removed, and the reaction mixture was slowly heated to 50 °C and stirred for 1 h which yielded a red-brown suspension. Afterwards all volatiles were removed *in vacuo* yielding a dark brown-red amorphous residue. NMR data did not show any typical shifts for a successful reaction yielding the ligand.

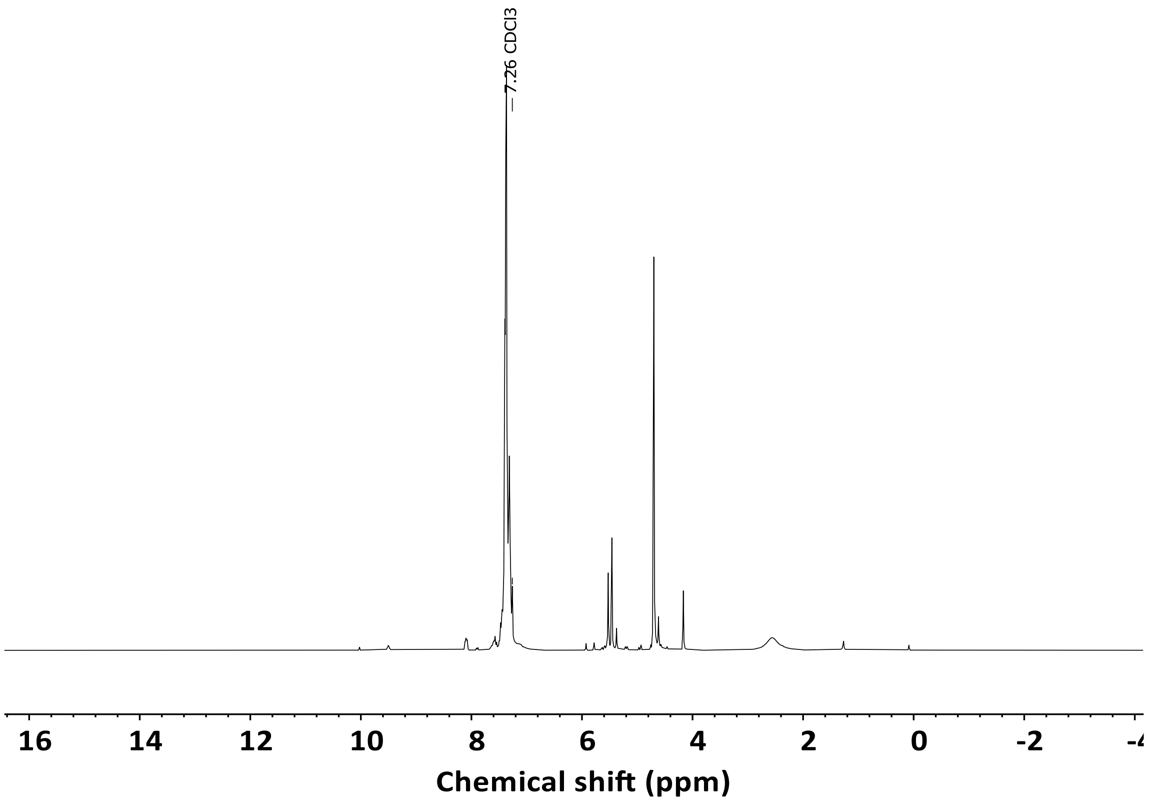

Figure S 39: ^{1}H-NMR spectrum of dark brown-red residue from the reaction with benzyl alcohol in $CDCl_3$ at room temperature.

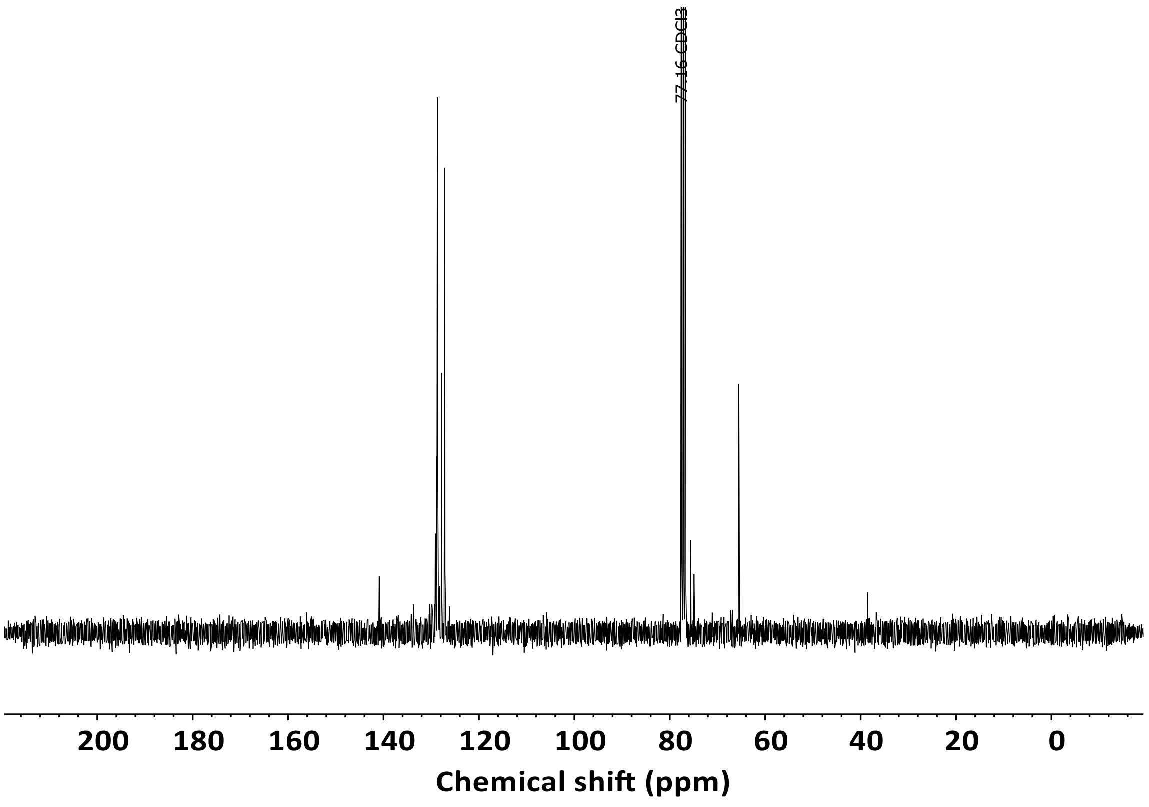

Figure S 40: ^{13}C-NMR spectrum of dark brown-red residue from the reaction with benzyl alcohol in $CDCl_3$ at room temperature.

Phenol

Thiocarbonyl dithiocyanate (330.00 mg, 2.06 mmol, 1.00 eq.) was dissolved in −25 °C precooled dichloromethane (20 mL) yielding a yellow solution. Then phenol (389.00 mg, 4.12 mmol, 2.00 eq.) was added and the reaction mixture was slowly heated to reflux for 2 h which yielded a red suspension. Afterwards all volatiles were removed *in vacuo* yielding a dark red oily residue. NMR data did not show any typical shifts for a successful reaction yielding the ligand.

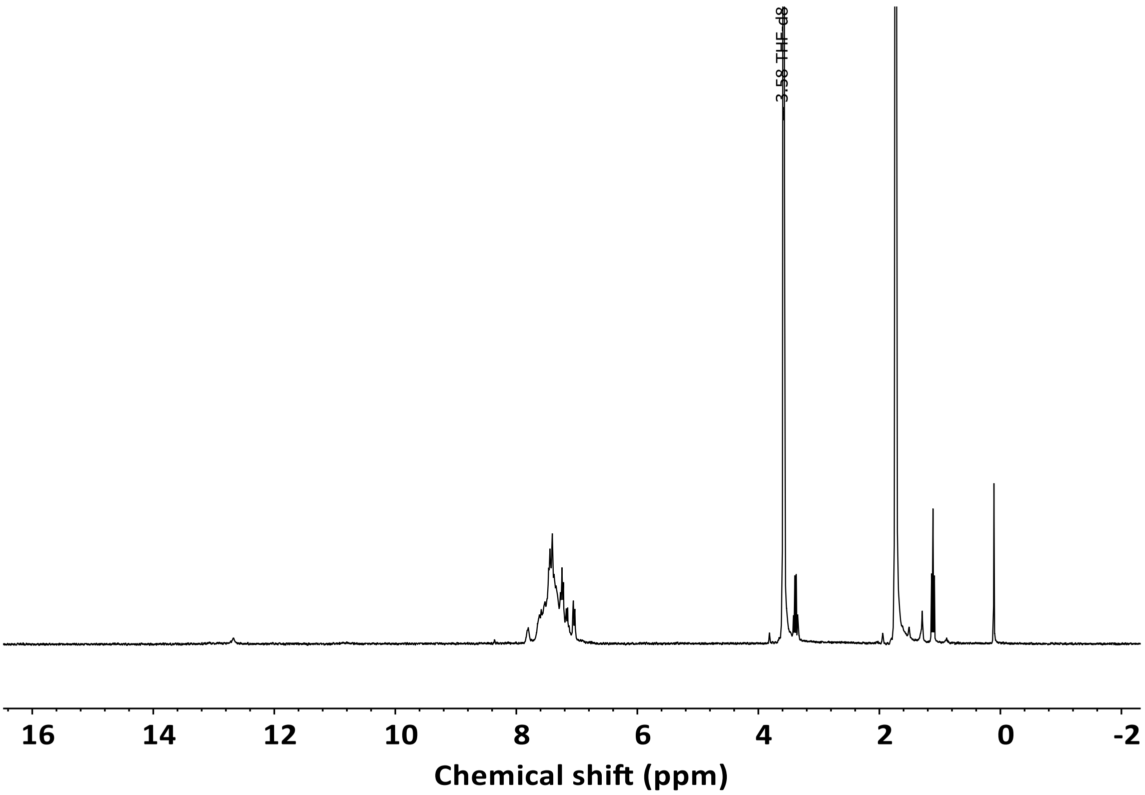

Figure S 41: ^{1}H-NMR spectrum of red oily residue from the reaction with phenol in $CDCl_3$ at room temperature.

3 Quantitative UV-Vis spectra

All (Quantitative) UV-Vis spectra were recorded at room temperature in dichloromethane using a Perkin-Elmer Lambda XLS+ spectrometer. For this, 0.00015 – 0.00025 M solutions of the corresponding complexes were prepared and measured using a standard UV-Vis cell (d = 1cm) without taking any precautions towards the exclusion of air and/or moisture.

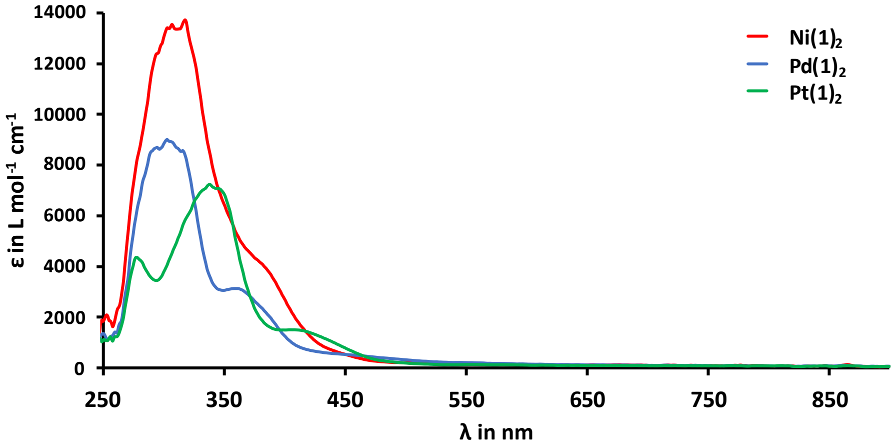

Figure S 42: Quantitative UV-Vis spectra of the reported nickel, palladium and platinum complexes of **1** recorded in dichloromethane.

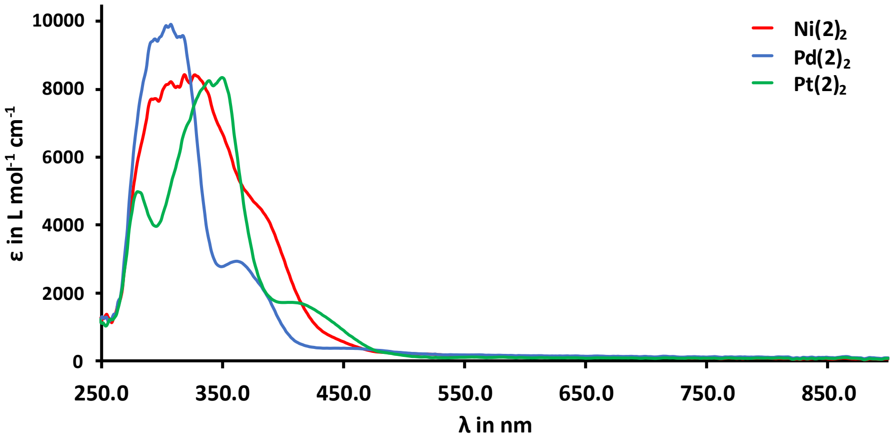

Figure S 43: Quantitative UV-Vis spectra of the reported nickel, palladium and platinum complexes of **2** recorded in dichloromethane.

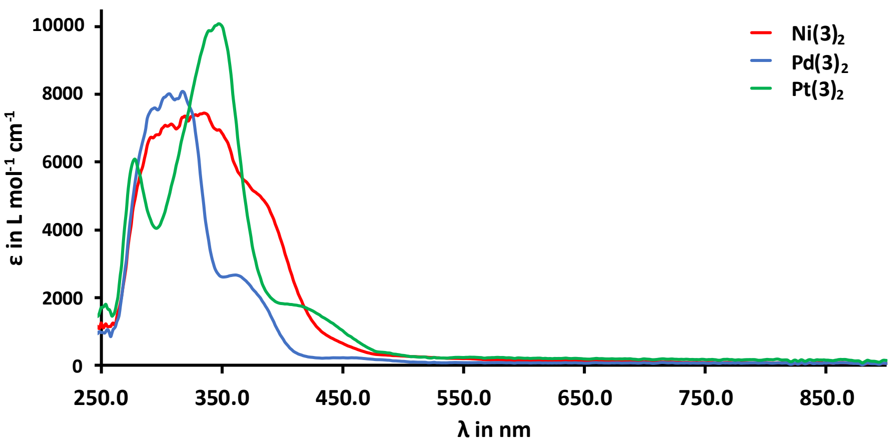

Figure S 44: Quantitative UV-Vis spectra of the reported nickel, palladium and platinum complexes of **3** recorded in dichloromethane.

Table S 1: Absorption maxima (m) and shoulders(sh) and corresponding molar extinction coefficients obtained by quantitative UV-Vis spectroscopy.

Compound	λ/nm (ε/L mol^{-1} cm^{-1})
Ni[**1**]$_2$	319 (13600) m, 387 (3800) sh
Ni[**2**]$_2$[2]	324 (8000) m, 384 (4300) sh
Ni[**3**]$_2$/°	333 (7400) m, 387 (4700) sh
Pd[**1**]$_2$	305 (8900) m, 359 (3100) m
Pd[**2**]$_2$	305 (9800) m, 363 (2900) m
Pd[**3**]$_2$	318 (8100) m, 364 (2600) m
Pt[**1**]$_2$	281 (4200) m, 340 (7200) m, 420 (1400) m
Pt[**2**]$_2$	282 (5000) m , 350 (8300) m , 419 (1600) m
Pt[**3**]$_2$	277 (6000) m, 350 (10000) m, 419 (1700) m

4 Crystallographic details

Single-crystal X-ray diffraction data were collected using an IPDS-2T (Stoe, Darmstadt) diffraction system equipped with mirror monochromated Mo Kα radiation (λ = 0.7107 Å, Xenocs Microfocus Source) and an Image Plate detector and a StadiVari (Stoe, Darmstadt) diffraction system equipped with mirror monochromated Cu K_{α} radiation (λ = 1.54186 Å, Xenocs Microfocus Source) and a Pilatus 300K detector. Crystals were selected under Paratone-N oil, mounted on micromount loops, and quench-cooled using an Oxford Cryosystems open-flow N_2 cooling device. Data were collected at 100 K and processed using the X-Area program package, including unit-cell parameter refinement and interframe scaling (which was carried out using LANA within X-Area). The structure was subsequently solved using direct dual-space methods (SHELXT)[3] and refined on F^2 with SHELXL[4] using the Olex2[5] user interface. The crystal structure drawings were generated with DIAMOND.[6]

Table S 2: Single crystal X-ray data collection and refinement parameters for **1**, **3**, [Li@12-crown-4][**2**], K[**3**], Ni[**1**]$_2$, and Pd[**1**]$_2$.

	**1**	**3**	[Li@12-crown-4][**2**]	K[**3**]	Ni[**1**]$_2$	Pd[**1**]$_2$
Formula	C4 H7 N O2 S2	C8 H15 N O2 S2	C14 H26 Li N O6 S2	C8 H14 K N O2 S2	C8 H12 N2 Ni O4 S4	C8 H12 N2 Pd O4 S4
CCDC	2450309	2450310	2450307	2450308	2450311	2450313
F. w. / g mol^{-1}	165.23	221.33	375.42	259.42	387.13	434.84
Crystal system	triclinic	monoclinic	monoclinic	orthorhombic	monoclinic	monoclinic
Space group	$P\bar{1}$	$P2_1/n$	$P2_1/n$	$Pna2_1$	$P2_1/c$	$P2_1/c$
a / Å	5.8447(5)	9.9684(3)	12.3241(2)	8.7251(5)	3.9708(4)	3.9606(4)
b / Å	7.8279(7)	8.0354(2)	8.27300(10)	23.397(2)	11.1573(10)	11.2115(8)
c / Å	8.3451(6)	14.9997(5)	18.5714(4)	12.3556(8)	15.6482(13)	15.7294(12)
α / °	84.228(7)	90	90	90	90	90
β / °	78.480(6)	106.461(2)	98.027(2)	90	95.966(7)	95.280(7)
γ / °	76.575(7)	90	90	90	90	90
V / Å^3	363.30(5)	1152.23(6)	1874.94(6)	2522.3(3)	689.51(11)	695.49(10)
Z	2	4	4	8	2	2
Radiation, λ / Å	CuKα (λ = 1.54186)	CuKα (λ = 0.71073)	CuKα (λ = 0.71073)	MoKα (λ = 1.54186)	MoKα (λ = 1.54186)	MoKα (λ = 1.54186)
Temp / K	100	100	100	100	100	100
ρ_{calc} / g cm^{-3}	1.510	1.276	1.330	1.366	1.865	2.076
μ / mm^{-1}	6.102	3.975	2.820	0.729	2.021	1.943
Reflections collected	6420	12814	20950	55004	15993	7084
Ind. Reflns. / Ind. Reflns gt	1362 / 1171	2170 / 1879	3545 / 2855	4441 / 3287	1833 / 1304	1228 / 857
Parameters	84	123	219	262	90	90
R_{int} / $R_{(\sigma)}$ / %	2.77 / 2.05	2.46 / 1.99	2.89 / 3.12	14.59 / 6.53	4.08 / 3.70	10.13 / 5.68
$R1$/$wR2$,[a] $I \geq 2\sigma I$ / %	4.35 / 12.43	2.69 / 7.25	2.47 / 2.47	3.74 / 7.05	3.93 / 9.38	5.52 / 13.11
$R1$/$wR2$,[a] all data / %	4.81 / 12.60	3.08 / 7.36	5.51 / 5.69	5.89 / 6.54	6.78 / 11.42	8.59 / 14.34
GOF	1.078	1.050	0.938	0.975	1.098	0.989
Twin law	—	—	—	1 0 0 0 −1 0 0 0 −1	—	—
BASF	—	—	—	0.2736	—	—

[a] $R1 = [\Sigma||F_o| - |F_c||]/\Sigma|F_o|$; $wR2 = \{[\Sigma w[(F_o)^2 - (F_c)^2]^2]/[\Sigma w(F_o{}^2)^2\}^{1/2}$; $w = [\sigma^2(F_o)^2 + (AP)^2 + BP]^{-1}$, where $P = [(F_o)^2 + 2(F_c)^2]/3$ and the A and B values are 0.0909 and 0.0530 for **1**, 0.0513 and 0.0000 for **3**, 0.0328, 0.0000 for Li@12-crown-4[**2**], 0.0259, 0.0000 for K[**3**], 0.0650 and 0.0000 for Ni[**1**]$_2$, 0.0961 and 0.096900 and 0.0000 for Pd[**1**]$_2$, and 0.0288 and 0.0000 for Pt[**1**]$_2$.

Table S 3: Single crystal X-ray data collection and refinement parameters for Pt[**1**], Pd[**2**]$_2$, Pt[**2**]$_2$. Ni[**3**]$_2$, Pd[**3**]$_2$ and Pt[**3**]$_2$.

	Pt[**1**]$_2$	Pd[**2**]$_2$	Pt[**2**]$_2$	Ni[**3**]$_2$	Pd[**3**]$_2$	Pt[**3**]$_2$
Formula	C8 H12 N2 Pt O4 S4	C12 H20 N2 O4 Pd S4	C12 H20 N2 O4 Pt S4	C16 H28 N2 Ni O4 S4	C16 H28 N2 O4 Pd S4	C16 H28 N2 O4 Pt S4
CCDC	2450316	2450315	2450318	2450312	2450314	2450317
F. w. / g mol^{-1}	523.53	490.94	579.63	499.35	547.04	635.754
Crystal system	monoclinic	orthorhombic	monoclinic	triclinic	monoclinic	monoclinic
Space group	$P2_1/c$	$Pna2_1$	$P2_1/c$	$P\bar{1}$	$P2_1/n$	$P2_1/n$
a / Å	3.9395(2)	14.8487(2)	18.8308(8)	7.4842(6)	7.3323(3)	7.3445(3)
b / Å	11.2020(6)	7.22330(10)	7.2206(2)	9.0139(7)	9.0815(4)	9.1008(2)
c / Å	15.7905(6)	17.3180(3)	14.8119(6)	9.2050(8)	16.9684(8)	16.8609(7)
α / °	90	90	90	66.960(6)	90	90
β / °	94.623(3)	90	113.109(3)	87.255(7)	100.753(4)	100.937(3)
γ / °	90	90	90	75.168(6)	90	90
V / Å^3	694.57(6)	1857.47(5)	1852.37(13)	551.46(8)	1110.06(9)	1106.53(7)
Z	2	4	4	1	2	2
Radiation, λ / Å	MoKα (λ = 1.54186)	CuKα (λ = 0.71073)	MoKα (λ = 1.54186)	MoKα (λ = 1.54186)	MoKα (λ = 1.54186)	MoKα (λ = 1.54186)
Temp / K	100	100	100	100	100	100
ρ_{calc} / g cm^{-3}	2.503	1.756	2.078	1.504	1.637	1.908
μ / mm^{-1}	10.711	12.433	8.043	1.282	1.236	6.740
Reflections collected	11758	35415	22993	8499	15179	20134
Ind. Reflns. / Ind. Reflns gt	2026 / 1385	3787 / 3286	4262 / 3405	3214 / 2503	3811 / 2694	3817 / 3271
Parameters	90	212	216	128	128	128
R_{int} / $R_{(\sigma)}$ / %	4.91 / 3.27	3.83 / 2.31	3.33 / 1.93	3.34 / 4.00	5.39 / 6.06	1.92 / 1.11
$R1/wR2$,[a] $I \geq 2\sigma I$ / %	2.09 / 4.42	3.01 / 7.43	326 / 856	3.42 / 8.33	3.13 / 5.35	1.43 / 3.20
$R1/wR2$,[a] all data / %	4.49 / 4.75	3.47 / 7.55	431 / 902	5.31 / 8.78	6.23 / 5.78	1.98 / 4.06
GOF	0.918	0.976	1.090	1.006	0.927	0.974
Twin law	—	—	1 0 1 0 −1 0 0 0 −1 2	—	—	—
BASF	—	—	0.06519	—	—	—

[a] $R1 = [\Sigma||F_o| - |F_c||]/\Sigma|F_o|$; $wR2 = \{[\Sigma w[(F_o)^2 - (F_c)^2]^2]/[\Sigma w(F_o^2)^2\}^{1/2}$; $w = [\sigma^2(F_o)^2 + (AP)^2 + BP]^{-1}$, where $P = [(F_o)^2 + 2(F_c)^2]/3$ and the A and B values are 0.0288 and 0.0000 for Pt[**1**]$_2$, 0.0572 and 0.0000 for Pd[**2**]$_2$, 0.053800 and 2.226600 for Pt[**2**]$_2$, 0.0547 and 0.0000 for Ni[**3**]$_2$, 0.028858 and 0.0000 for Pd[**3**]$_2$ and 0.017416 2.838632 for Pt[**3**]$_2$.

4.1 Additional pictures of crystal structures

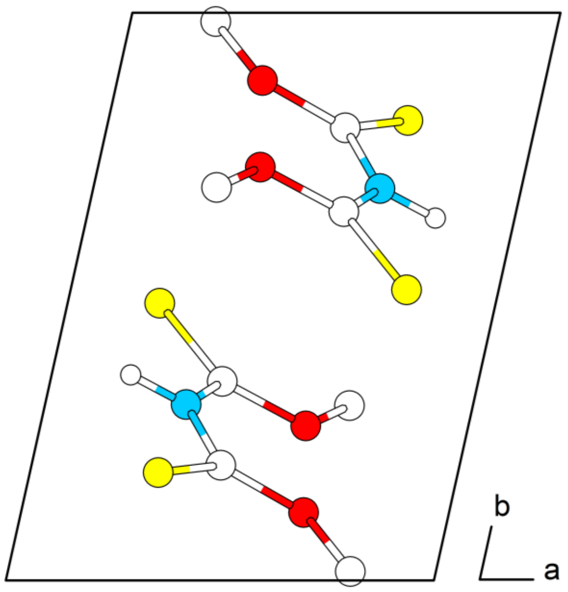

Figure S 45: Crystal structure of **1** viewed along [001]. Atoms are drawn with arbitrary radii. Aliphatic hydrogen atoms are omitted for clarity. Colour code: S yellow, C white, N blue, H white.

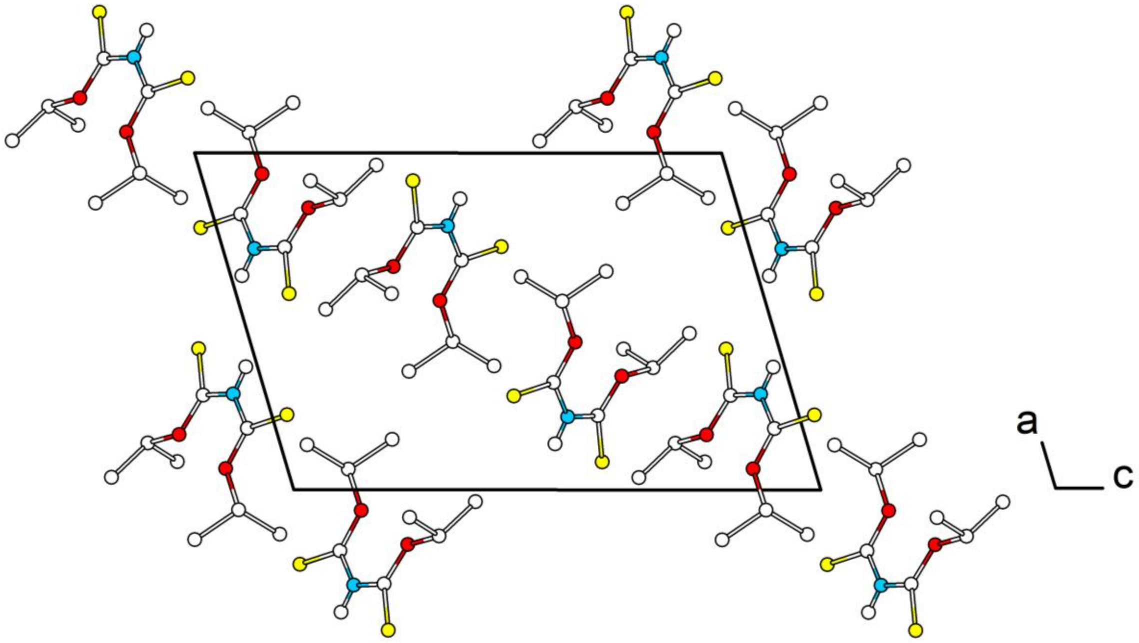

Figure S 46: Crystal structure of **3** viewed along [010]. Atoms are drawn with arbitrary radii. Aliphatic hydrogen atoms are omitted for clarity. Colour code: S yellow, C white, N blue.

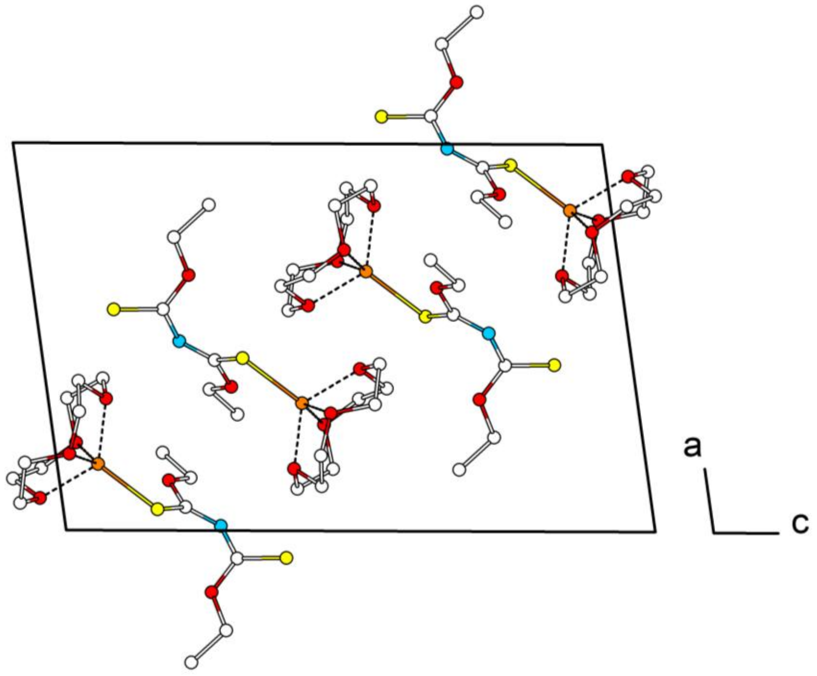

Figure S 47: Crystal structure of Li@12-crown-4[**2**] viewed along [010]. Atoms are drawn with arbitrary radii. Aliphatic hydrogen atoms are omitted for clarity. Colour code: S yellow, C white, N blue, Li orange.

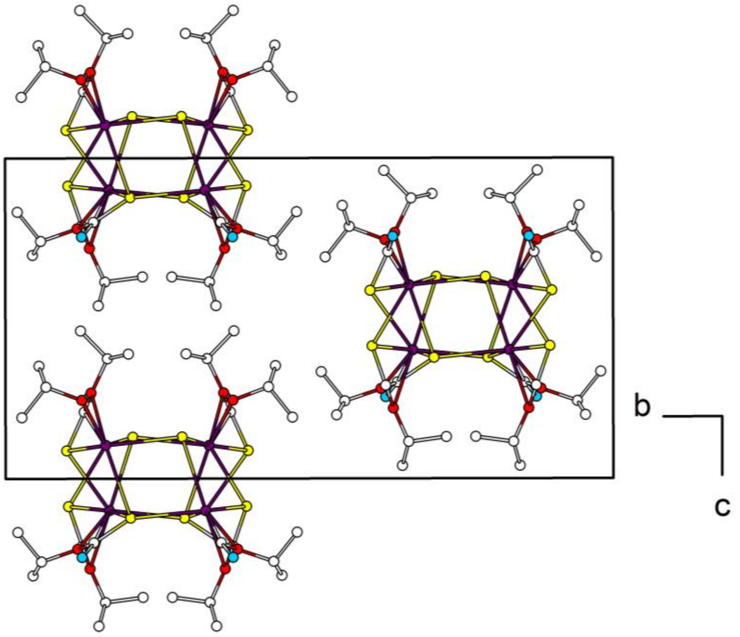

Figure S 48: Crystal structure of K[**3**] along [100]. Atoms are drawn with arbitrary radii. Aliphatic hydrogen atoms are omitted for clarity. Colour code: S yellow, C white, N blue, K purple.

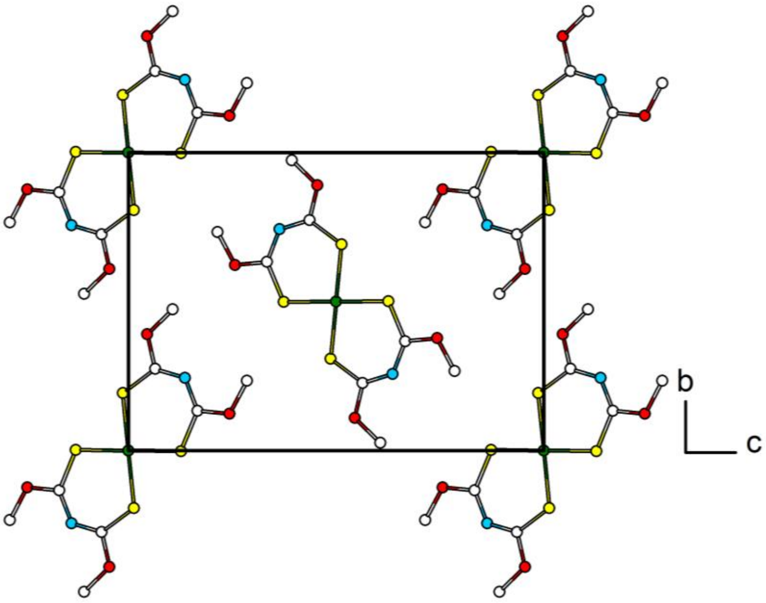

Figure S 49: Crystal structure of Ni[**1**]$_2$ viewed along [100]. Pd[**1**]$_2$ and Pt[**1**]$_2$ crystallize isotopically. Atoms are drawn with arbitrary radii. Colour code: S yellow, C white, N blue, Ni green

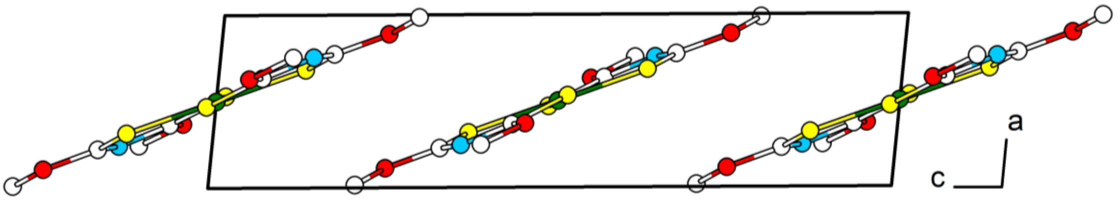

Figure S 50: Crystal structure of Ni[**1**]$_2$ viewed along [100]. Pd[**1**]$_2$ and Pt[**1**]$_2$ crystallize isotopically. Atoms are drawn with arbitrary radii. Colour code: S yellow, C white, N blue, Ni green

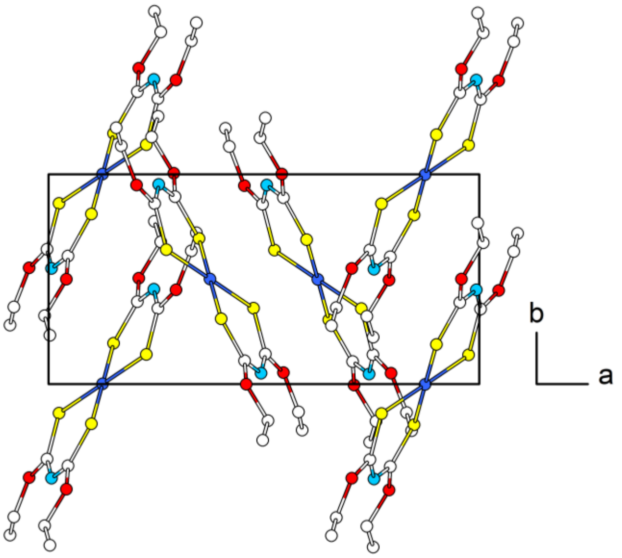

Figure S 51: Crystal structure of Pd[**2**]$_2$ view along [001]. Atoms are drawn with arbitrary radii. Colour code: S yellow, C white, N blue, Pd blue.

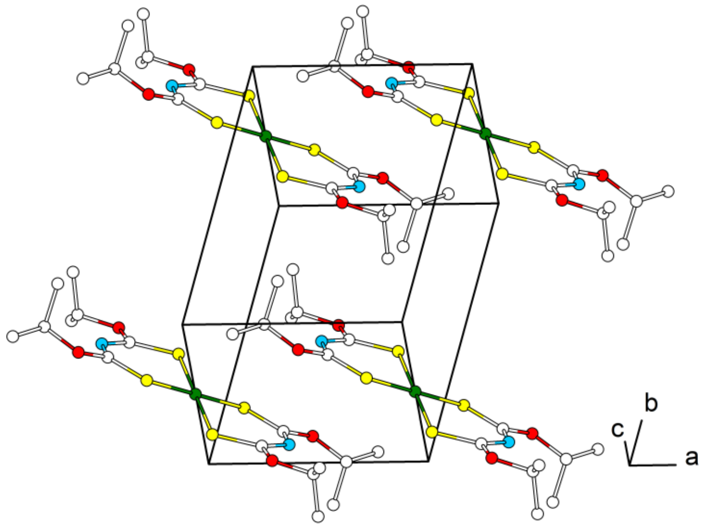

Figure S 52: Crystal structure of Ni[**3**]$_2$. Atoms are drawn with arbitrary radii. Colour code: S yellow, C white, N blue, Ni green.

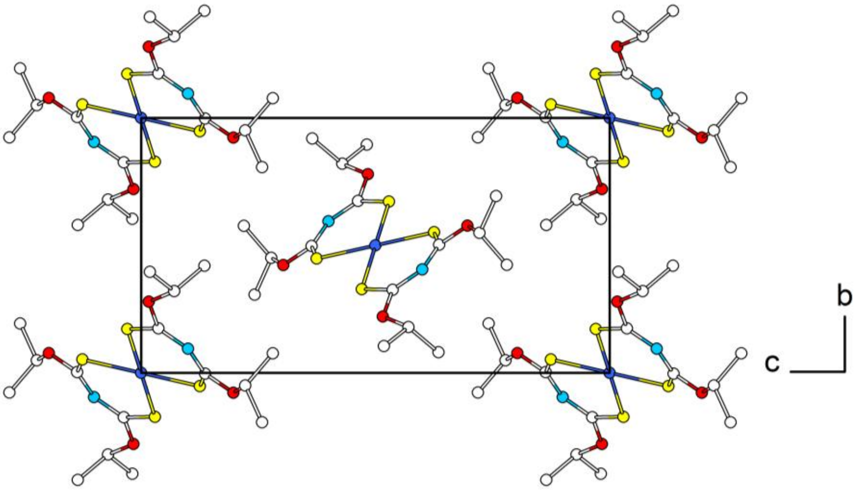

Figure S 53: Crystal structure of Pd[**3**]$_2$ viewed along [010]. Pt[**3**]$_2$ crystallizes isotopically Atoms are drawn with arbitrary radii. Colour code: S yellow, C white, N blue, Pd blue.

4.2 Additional tables

Table S 4: Selected bond lengths and angles of Ni[**1**–**3**]$_2$, Pd[**1**–**3**]$_2$ and Pt[**1**–**3**]$_2$.

Compound	C–S / Å	C–N / Å	S–*M* / Å	S–M–S / °
Ni[**1**]$_2$	1.689(3), 1.701(3)	1.320(4), 1.309(4)	2.1750(8), 2.1646(8)	97.17(3)
Ni[**2**]$_2$[2]	1.711(3), 1.708(3), 1.712(3), 1.706(3)	1.319(4), 1.324(4), 1.319(3), 1.327(4)	2.1647(5), 2.1686(7), 2.1665(6), 2.1675(6)	96.88(2), 96.70(2)
Ni[**3**]$_2$	1.704(2), 1.701(2)	1.319(2), 1.316(2)	2.1597(5), 2.1548(5)	96.68(2)
Pd[**1**]$_2$	1.684(8), 1.683(7)	1.3111(97), 1.3166(94)	2.287(2), 2.273(2)	96.15(6)
Pd[**2**]$_2$	1.707(8), 1.710(7), 1.705(8), 1.713(8)	1.318(9), 1.320(9), 1.33(1), 1.313(9)	2.285(2), 2.287(2), 2.288(2), 2.284(2)	95.57(7), 95.69(7)
Pd[**3**]$_2$	1.697(2), 1.704(2)	1.314(3), 1.316(3)	2.2738(5), 2.2804(5)	95.93(2)
Pt[**1**]$_2$	1.693(5), 1.692(4)	1.314(5), 1.317(5)	2.2788(8), 2.274(1)	96.91(4)
Pt[**2**]$_2$	1.702(5), 1.702(5), 1.697(5), 1.706(6)	1.325(5), 1.327(6), 1.321(7), 1.320(7)	2.283(1), 2.283(1), 2.283(2), 2.280(1)	96.54(4), 96.43(4)
Pt[**3**]$_2$	1.698(2), 1.705(2)	1.318(3), 1.314(2)	2.274(5), 2.277(5)	96.32(2)

Table S 5: Yields (in %) of Ni[**1**–**3**]$_2$, Pd[**1**–**3**]$_2$ and Pt[**1**–**3**]$_2$.

Moiety	Me	Et	^{i}Pr
Ni^{2+}	59	76[2]	72
Pd^{2+}	53	63	82
Pt^{2+}	62	60	56

4. Literature

[1] W. L. F. Armarego, C. L. L. Chai, *Purification of Organic Chemicals*, **2009**.
[2] J. Pfeiffer, H. Günther, P. Fuzon, F. Weigend, F. Tambornino, "Thiocarbonyl Pseudohalides – The Curious Case of Thiocarbonyl Dithiocyanate" *Chem. – Eur. J.* **2024**, *30*, e202401508.
[3] G. M. Sheldrick, "SHELXT – Integrated space-group and crystal-structure determination" *Acta Crystallogr. Sect. Found. Adv.* **2015**, *71*, 3–8.
[4] G. M. Sheldrick, "A short history of *SHELX*" *Acta Crystallogr. A* **2008**, *64*, 112–122.
[5] O. V. Dolomanov, L. J. Bourhis, R. J. Gildea, J. A. K. Howard, H. Puschmann, "*OLEX2* : a complete structure solution, refinement and analysis program" *J. Appl. Crystallogr.* **2009**, *42*, 339–341.
[6] Brandenburg, K., Putz, H., *DIAMOND, Program for X-Ray Structure Analysis*, Crystal Impact GbR, Bonn, Germany, **1999**.
